# Uncovering the potential of Yiqi Huoxue Jiedu formula: a promising adjunct in sepsis and septic shock management

**DOI:** 10.3389/fcimb.2025.1655393

**Published:** 2025-10-15

**Authors:** Peiying Huang, Bingrui Chen, Xiaorong Yang, Houguang Chen, Jiaqi Tang, Rui Chen, Jun Li, Ye Ye

**Affiliations:** ^1^ Guangdong Provincial Second Hospital of Traditional Chinese Medicine, Guangdong Provincial Engineering Technology Research Institute of Traditional Chinese Medicine, The Fifth Clinical College of Guangzhou University of Chinese Medicine, Guangdong Provincial Key Laboratory of Research and Development in Traditional Chinese Medicine, Guangzhou, China; ^2^ Guangdong Provincial Hospital of Chinese Medicine, The Second Affiliated Hospital of Guangzhou University of Chinese Medicine, Guangzhou, China; ^3^ The Second Clinical Medical College of Guangzhou University of Chinese Medicine, Guangzhou, China; ^4^ The First Affiliated Hospital of Guangzhou University of Chinese Medicine, Guangzhou, China; ^5^ Chinese Medicine Guangdong Laboratory, Zhuhai, China

**Keywords:** Yiqi Huoxue Jiedu formula, sepsis, septic shock, absorbed components, clinical efficacy

## Abstract

**Background:**

Sepsis and septic shock represent severe infectious conditions with challenging treatments. Preliminary animal studies indicate that Yiqi Huoxue Jiedu Formula (YHJF) may enhance sepsis outcomes, but no clinical trials have been conducted. This research aims to substantiate the efficacy of YHJF in treating sepsis and septic shock.

**Materials and methods:**

Mass spectrometry was used to identify the absorbed constituents of YHJF. A retrospective cohort study included sepsis patients from four sub-centers (Feb 24, 2016 - Oct 31, 2024), divided into Western treatment and YHJF plus Western care groups. Propensity score matching ensured baseline comparability. The primary outcome was 28-day mortality, with secondary outcomes including intensive care units (ICU) and hospital stay duration, mechanical ventilation and renal replacement therapy duration, fever duration, and changes in SOFA and APACHE II scores, blood procalcitonin, white blood cell count, and lactate levels after 5 days of treatment. Sepsis shock was analyzed as a subgroup.

**Results:**

YHJF absorption mainly includes Shikimates and Phenylpropanoids (33%), Terpenoids (21%), Alkaloids (14%), and Polyketides (11%). A total of 389 sepsis patients were enrolled, with 82 patients being identified as septic shock in the clinical study. The clinical results indicated that YHJF may shorten hospital stay duration, reduce ventilator duration, and improve SOFA score in sepsis. For septic shock, YHJF administration could reduce the duration of non-invasive ventilator use. Notably, YHJF has no effect on improving 28-day mortality for sepsis and septic shock.

**Conclusion:**

YHJF can reduce the SOFA score, ventilator use duration, and hospital stay length in sepsis cases and improve non-invasive ventilator use time in septic shock patients, which might attribute to the absorbed components after YHJF administration. Further high-quality trials are needed for confirmation.

## Introduction

Sepsis, as defined by Sepsis 3.0, is a life-threatening condition caused by infection-induced organ dysfunction. Septic shock is a more severe form, characterized by persistent low blood pressure despite fluid treatment (Singer et al., 2016). Globally, there are approximately 20 to 30 million individuals affected by sepsis annually and an estimated 8 million fatalities attributed to the condition (Van Looveren et al., 2020). Research conducted in Australia indicates that annually, 5,000 adults are treated for sepsis in intensive care units (ICU), with the majority experiencing long-term physical, cognitive, and psychological impairments post-recovery (Thompson et al., 2019). Septic shock has notably higher mortality rates (Rech et al., 2019), with a Japanese study reporting a 28.5% ICU mortality rate for such cases (Takauji et al., 2020).

The current management of sepsis and septic shock focuses on early identification, appropriate antibiotic administration, source control, fluid resuscitation, and organ function support (Moss and Prescott, 2019). Ongoing research is focusing on novel strategies. For example, precision medicine is develop to identify specific patient subgroups and devise personalized treatment plans to improve prognosis (Moss and Prescott, 2019). Additionally, immunotherapy is considered another promising approach. Although specific immunomodulatory drugs for sepsis are not yet available, modulating the body’s immune response may enhance long-term prognosis (Marques et al., 2023). Despite these efforts, sepsis and septic shock remain complex and challenging, with high mortality, especially in ICU, highlighting the need for further treatment innovations (McEvoy and Kollef, 2013).

Herbal medicines serve as effective adjuvant therapeutic agents for numerous infectious diseases (Liang et al., 2022; Yuan, 2022). Yiqi Huoxue Jiedu Formula (YHJF, Patent Number: ZL202211547052.1) is an herbal compound consisting of Ginseng Root (*Ginseng Radix et Rhizoma*), Sanchi (*Notoginseng Radix et Rhizoma*), and Rhubarb (*Rhei Radix et Rhizoma*). Initial clinical observations suggested that this formula might possess potential benefits in treating sepsis and septic shock. Subsequent study demonstrated that YHJF can enhance the 7-day survival rate in septic mice (Chen et al.). Nonetheless, these results were derived from clinical observations or animal research. There remains a paucity of evidence-based data regarding the efficacy of YHJF in clinical settings for these conditions. Therefore, we conducted a retrospective cohort study comparing sepsis and septic shock patients treated with YHJF in 4 sub-centers to those who did not receive herbal treatment, to evaluate YHJF’s clinical efficacy.

## Method

### Preclinical chemical analysis of YHJF

#### UHPLC-HRMS analysis for YHJF

We analyzed the chemical components in YHJF’s aqueous extract and rat serum after YHJF administration using Ultra High-Performance Liquid Chromatography - High Resolution Mass Spectrometry (UHPLC-HRMS) on a Q-Exactive HFX system. Chemical identification was done by comparing the primary accurate mass (with less than 25 ppm error) and secondary fragmentation spectra to a local standard compound database. The identified compounds were then classified using NPClassifier, a deep neural network-based tool for organizing natural product structures into Pathway, Superclass, and Class (Kim et al., 2021).

The UHPLC-HRMS analysis included the YHJF aqueous extract, YHJF drug-containing serum, control serum, and control serum plus YHJF aqueous extract. One dose of YHJF formulation consists of Ginseng Root (15 g), Sanchi (9 g), and Rhubarb (3 g). The preparation involves soaking the herbs in water (1:8 w/v) for two hours, simmering for 30 minutes, adding more distilled water (4 times the herb weight), and simmering again for 30 minutes. After that, the extracts are combined, filtered, and concentrated to 1.0 g·mL^-^¹ to yield the YHJF aqueous extract.

Ten 7-week-old male Sprague-Dawley rats were divided into experimental and control groups (5 each). The experimental group received YHJF aqueous extract orally at 1.0 mL·100 g^-^¹ body weight for four times, with doses given 20 hours apart initially and then every 4 hours. The control group received the same schedule with saline. Blood samples were collected 3 hours after the final dose for both groups.

### Retrospective cohort study

#### Inclusion and exclusion criteria

We collected patients diagnosed with sepsis or septic shock in the electronic medical record system from 4 ICUs of Guangdong Provincial Hospital of Traditional Chinese Medicine, including the Dade Road Headquarters, Ersha Island Branch Center, Fangcun Branch Center, and Guangzhou Higher Education Mega Center Branch Center, during the period from February 24, 2016, to October 31, 2024. Most patients originate from Guangdong Province, China, with a smaller proportion coming from adjacent provinces and cities. The service area encompasses approximately 450,000 square kilometers.

The inclusion criteria are as follows:

1. Patient aged ≥ 18 years;
2. Sepsis and septic shock were diagnosed according to Sepsis 3.0 criteria and the intervention is initiated within 72 hours of the diagnosis(1);
3. Following a confirmed diagnosis, patients must have either received YHJF for a minimum of two days or not have taken any herbal preparations;
4. Baseline characteristics must be available at the time of admission or sepsis onset, including age, gender, Body Mass Index (BMI), heart rate, systolic and diastolic blood pressure, body temperature, respiratory rate, Peripheral Capillary Oxygen Saturation (SPO_2_), initial infection source, underlying conditions, Sequential Organ Failure Assessment (SOFA) score, Acute Physiology and Chronic Health Evaluation II (APACHE II) score, anti-infection strategies, blood culture status, procalcitonin levels, white blood cell count, and blood lactate levels;
5. Outcome data can be collected, including 28-day mortality, duration of ICU stays, total length of hospital stays, duration of non-invasive and invasive ventilator use, length of continuous renal replacement therapy (CRRT), number of febrile days, SOFA and APACHE II scores, procalcitonin levels, white blood cell count, and blood lactate in the fifth days post-treatment.

The exclusion criteria are as follows:

1. Patient received other herbal extract preparations;
2. Patient had severe underlying conditions, including coagulation disorders, advanced malignant tumors, acquired immunodeficiency syndrome, post-cardiopulmonary resuscitation, and severe cardiovascular and cerebrovascular diseases such as acute coronary syndrome, as well as pre-existing organ dysfunctions like chronic renal failure;
3. YHJF-taking patient had the condition of gastric retention or intestinal malabsorption;
4. Patient was either pregnant or lactating;
5. Patient had undergone organ transplantation or had used immunosuppressants within 6 months before disease onset;
6. Patient had a history of mental disorders;
7. Patient was concurrently enrolled in other clinical studies.

### Routine treatment protocol

All patients received treatment systematically in alignment with the protocols outlined in the Western medicine guidelines(1). Patients in the YHJF group received only YHJF alongside the standard treatment protocols either orally or via a nasogastric tube. The YHJF herbal decoction was prepared by the hospital’s preparation center. One dose of crude drug (Ginseng Root 15 g, Sanchi 9 g, and Rhubarb 3 g) was boiled into two doses of 50–100 ml each, with one dose administered in the morning and the other in the evening daily. Inclusion in the study required patients to have received YHJF for a minimum of two days. In contrast, patients in the control group were treated solely according to the Western medicine guidelines and did not receive any herbal preparations during the efficacy evaluation period.

### Exposure

The exposure variable is the administration of YHJF.

### Outcomes

The primary outcome is the 28-day mortality. Secondary outcomes encompass the length of stay in the ICU, the duration of hospitalization, the number of febrile days, the duration of both invasive and non-invasive ventilation, the duration of CRRT usage, and white blood cell count, procalcitonin levels, blood lactate levels, and the SOFA and APACHE II scores recorded on the fifth day post-treatment.

### Ethics

The animal experimentation has received approval from the Ethics Committee of Guangdong Provincial Hospital of Traditional Chinese Medicine with approval number 2024085. The clinical research is designed as a retrospective cohort study and following deliberations with the Ethics Committee, it was unnecessary to obtain informed consent from the patients. Notably, the clinical data were anonymized or de-identified in compliance with ethical standards. This study adheres to the ethical principles outlined in the World Medical Association’s Declaration of Helsinki.

### Data analysis

In this study, an imbalance was observed in the patient quantity ratio and baseline characteristics between the YHJF group and the control group. To reduce confounding, 1:1 propensity score matching (PSM) was performed based on key baseline covariates. The analysis used the MatchIt package in R project. Optimal matching was chosen based on standardized mean difference (SMD), variance ratio (Var.Ratio), and empirical cumulative distribution function (eCDF), aiming for an SMD and eCDF near 0 and a Var.Ratio near 1 for better baseline balance. Love plot was used to visualize data distribution changes pre- and post-matching.

For inter-group comparisons of continuous variables, the Shapiro-Wilk test checked normality. Normally distributed data were shown as mean ± standard deviation and compared using the t-test. Non-normally distributed data were presented with rank averages and distribution intervals, using the Mann-Whitney U test for comparisons. Categorical variables were expressed as frequency and percentage, with the chi-square test used for comparisons. Furthermore, to evaluate the impact of YHJF administration and its duration on the outcomes, Cox regression and linear regression models were constructed with the primary outcome and statistically significant secondary outcomes as the dependent variables, respectively. Covariates included in the model were those variables that demonstrated statistical significance and clinical rationality in the inter-group comparison. Inter-group comparisons were conducted using SPSS 18.0, while regression analyses were performed utilizing R project. The statistical significance threshold was *p* < 0.05.

To assess the specific therapeutic effects of YHJF on septic shock, a subgroup analysis focusing on septic shock was performed. This subgroup comprised exclusively of patients diagnosed with septic shock in both the YHJF and control groups. The statistical analysis methods employed were consistent with those previously described.

## Result

### Preclinical chemical analysis of YHJF

#### UHPLC-HRMS analysis

**Figure 1:**
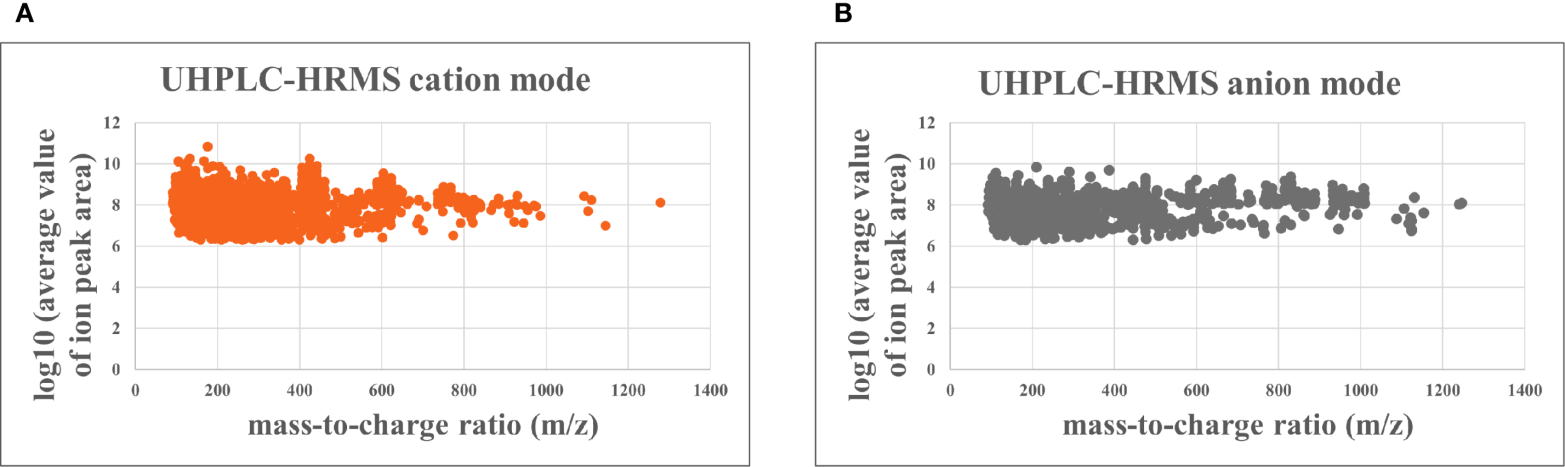
The mass-to-charge ratio (m/z) of parent ion (abscissa) and the log10 (average value of ion peak area) (ordinate) for the identified compounds. The mass-to-charge ratio is used to determine the exact molecular weight of the compound, while the log10 (average value of ion peak area) reflects its relative abundance. **(A)** cation analysis; **(B)** anion analysis. UHPLC-HRMS, Ultra High-Performance Liquid Chromatography - High Resolution Mass Spectrometry.

UHPLC-HRMS analysis revealed 1,378compounds in the YHJF aqueous extract and 158 compounds in the YHJF-containing serum. Utilizing the NPClassifier classification, all identified compounds were categorized into 17 Pathways and 88 Superclasses (**Supplementary Figure 1**; **Table 1**). The compounds detected in the serum were associated with 8 Pathways and 35 Superclasses (**Supplementary Figure 2**; **Table 2**). The mass-to-charge ratio of parent ion and the log10 (average value of ion peak area) are illustrated in **Figure 1**. The cation and anion base peak chromatograms (BPC) for all samples, along with the comprehensive list of the identified compounds, are available in **Supplementary Figures 3**, **4**; **Tables 3**, **4**.

**Table 1:** Baseline characteristics of the YHJF and control groups before and after PSM.

Characteristics	Before propensity score matching			After propensity score matching		
	YHJF group *n* = (159)	Control group *n* = (230)	*p* valve	YHJF group *n* = (159)	Control group *n* = (159)	*p* valve
Sex (Men)	90 (56.60)	133 (57.80)	0.811	90 (56.60)	90 (56.60)	1.000
Age	77 (62.0-84.0)	76.0 (68.25-85.0)	0.618	77 (62.00-84.00)	75 (69.00-85.00)	0.506
BMI	21.39 (19.48-23.8)	20.65 (18.3-22.98)	0.009^*^	21.39 (19.48-23.80)	21.20 (18.60-23.10)	0.286
Heart rate	97 (83.75-109.25)	96.5 (8125-109.0)	0.843	97 (83.75-109.25)	96 (81.00-110.00)	0.808
Systolic blood pressure	119 (99.75-142.25)	124 (111.0-147.0)	0.010^*^	119 (99.75-142.25)	124 (111.00-144.00)	0.057
Diastolic blood pressure	70.5 (61.0-81.25)	70.5 (62.0-82.75)	0.916	70.50 (61.00-81.25)	71.00 (62.00-84.00)	0.740
Body temperature	37.2 (36.6-37.92)	36.8 (36.5-37.88)	0.013^*^	37.20 (36.60-37.93)	36.90 (36.50-38.00)	0.129
Respiratory rate	20 (20.0-25.0)	21 (20.0-25.0)	0.575	20 (20.00-25.00)	21 (20.00-25.00)	0.348
SPO_2_	98 (96.0-100.0)	99 (96.0-100.0)	0.052	98.0 (96.00-100.00)	98.6 (96.00-100.00)	0.160
Using antibiotics	159 (100.00)	229 (99.60)	0.405	159 (100.00)	159 (100.00)	1.000
Using antiviral drug	21 (13.21)	27 (11.70)	0.665	21 (13.21)	18 (11.32)	0.608
Using antifungal drug	23 (14.47)	55 (23.90)	0.022^*^	23 (14.47)	28 (17.61)	0.445
Present as septic shock	45 (28.30)	36 (15.70)	0.003^*^	45 (28.30)	33 (20.75)	0.118
Positive blood culture	48 (30.19)	50 (21.70)	0.059	48 (30.19)	40 (25.16)	0.316
Source of infection						
Lung infection	125 (78.62)	174 (75.70)	0.496	125 (78.62)	122 (76.73)	0.686
Digestive tract infection	6 (3.77)	15 (6.50)	0.238	6 (3.77)	7 (4.40)	0.777
Urinary infection	34 (21.38)	49 (21.30)	0.985	34 (21.38)	36 (22.64)	0.787
Skin or soft tissue infection	3 (1.89)	5 (2.20)	0.844	3 (1.89)	4 (2.52)	0.702
Underlying comorbidities						
Hypertension	85 (53.46)	145 (63.00)	0.059	85 (53.46)	100 (62.89)	0.088
Diabetes	55 (34.59)	75 (32.60)	0.684	55 (34.59)	59 (37.11)	0.640
Coronary heart disease	39 (24.53)	46 (20.00)	0.288	39 (24.53)	36 (22.64)	0.692
Stroke	29 (18.24)	57 (24.80)	0.126	29 (18.24)	37 (23.27)	0.269
Chronic obstructive pulmonary disease	15 (9.43)	23 (10.00)	0.853	15 (9.43)	14 (8.81)	0.846
Liver diseases	41 (25.79)	55 (23.90)	0.674	41 (25.79)	39 (24.53)	0.796
Kidney diseases	52 (32.70)	95 (41.30)	0.085	52 (32.70)	56 (35.22)	0.636
Before-treatment SOFA scores	4.0 (2.0-6.0)	4.5 (2.0-8.0)	0.083	4.00 (2.00-6.00)	4.00 (2.00-6.00)	0.754
Before-treatment APACHE II scores	22 (12.0-37.0)	22 (12.0-37.0)	0.211	22 (12.00-37.00)	22 (12.00-37.00)	0.734
Before-treatment procalcitonin	6.32 (1.23-20.42)	1.58 (0.42-8.36)	0.000^*^	6.32 (1.23-20.42)	1.53 (0.55-8.37)	0.054
Before-treatment white blood cell count	13.34 (9.3-18.54)	11.28 (8.02-15.67)	0.013^*^	13.34 (9.30-18.55)	11.73 (8.73-17.11)	0.196
Before-treatment blood lactic acid	2.1 (1.5-3.6)	1.7 (1.1-2.8)	0.000^*^	2.10 (1.50-3.60)	1.80 (1.20-2.90)	0.209
Duration of taking YHJF	4 (2.0-6.25)	0 (0.0-0.0)	0.000^*^	4 (2.00-6.25)	0 (0.00-0.00)	0.000^*^

^*^ *p* < 0.05. Normally distributed data were shown as mean ± standard deviation (mean ± SD), non-normally distributed data were presented with rank averages and distribution intervals [RA (minimum-maximum), and categorical data were expressed as frequency and percentage (n (%)]. YHJF, Yiqi Huoxue Jiedu Formula; PSM, Propensity score matching; BMI, Body Mass Index; SPO_2_, Peripheral Capillary Oxygen Saturation; SOFA, Sequential Organ Failure Assessment; APACHE II, Acute Physiology and Chronic Health Evaluation II.

**Table 2:** Comparative outcomes between YHJF and control groups in the sepsis cohort.

Outcomes	YHJF group *n* = (159)	Control group *n* = (159)	*p* valve
28-day mortality	24 (15.09)	18 (11.32)	0.320
Duration of ICU stays	0 (0.00-6.00)	2 (0.00-13.00)	0.009^*^
Duration of hospitalization	10 (6.00-17.00)	16 (11.00-25.00)	0.000^*^
Length of noninvasive ventilation	0 (0.00-12.13)	0 (0.00-46.00)	0.028^*^
Length of invasive ventilation	0 (0.00-0.00)	0 (0.00-21.78)	0.029^*^
Length of CRRT	0 (0.00-0.00)	0 (0.00-0.00)	0.530
Duration of fever	3 (1.00-6.00)	4 (1.00-9.00)	0.285
Day 5 SOFA scores	2 (1.00-3.00)	2 (2.00-3.00)	0.007^*^
Day 5 APACHE II scores	17.00 (14.00-20.00)	18.00 (16.00-21.00)	0.017^*^
Day 5 procalcitonin	0.97 (0.34-3.65)	1.08 (0.28-4.93)	0.660
Day 5 white blood cell count	8.25 (5.90-11.60)	9.10 (6.49-11.80)	0.153
Day 5 blood lactic acid	1.4 (1.10-1.90)	1.5 (1.20-2.00)	0.181

^*^ *p*<0.05. Normally distributed data were shown as mean ± standard deviation (mean ± SD), non-normally distributed data were presented with rank averages and distribution intervals (RA (minimum-maximum), and categorical data were expressed as frequency and percentage (n (%)). YHJF, Yiqi Huoxue Jiedu Formula; ICU, Intensive care unit; CRRT, Continuous renal replacement therapy; SOFA, Sequential Organ Failure Assessment; APACHE II, Acute Physiology and Chronic Health Evaluation II.

**Table 3:** Baseline characteristics of the YHJF and control groups before and after PSM in the septic shock cohort.

Characteristics	Before propensity score matching			After propensity score matching		
	YHJF group *n* = (45)	Control group *n* = (37)	*p* valve	YHJF group *n* = (37)	Control group *n* = (37)	*p* valve
Sex (Men)	30 (66.70)	23 (62.20)	0.671	23 (62.20)	23 (62.20)	1.000
Age	72.00 (60.50-83.00)	75.00 (69.00-83.00)	0.685	72.00 (61.50-83.00)	75.00 (69.00-83.00)	0.778
BMI	20.60 (18.45-23.35)	21.80 (18.82-23.15)	0.618	20.80 (18.25-23.35)	21.80 (18.81-23.15)	0.630
Heart rate	103.44 ± 20.78	106.19 ± 20.22	0.549	104.03 ± 19.37	106.19 ± 20.22	0.640
Systolic blood pressure	98.00 (88.50-118.00)	102.00 (90.00-143.00)	0.261	97.00 (86.00-118.00)	102.00 (90.00-143.00)	0.152
Diastolic blood pressure	65.76 ± 16.77	68.24 ± 21.00	0.553	65.30 ± 15.94	68.24 ± 21.00	0.499
Body temperature	37.30 (36.50-37.90)	36.90 (36.50-38.20)	0.511	37.30 (36.50-37.90)	36.90 (36.50-38.20)	0.537
Respiratory rate	21.00 (18.00-26.50)	22.00 (20.00-26.50)	0.321	22.00 (18.00-26.50)	22.00 (20.00-26.50)	0.410
SPO_2_	98.00 (94.50-100.00)	98.00 (96.50-100.00)	0.536	97.00 (94.50-100.00)	98.00 (96.50-100.00)	0.470
Using antibiotics	45 (100.00)	37 (100.00)	1.000	37 (100.00)	37 (100.00)	1.000
Using antiviral drug	4 (8.90)	5 (13.50)	0.505	3 (8.10)	5 (13.50)	0.454
Using antifungal drug	5 (11.10)	14 (37.80)	0.004^*^	5 (13.50)	14 (37.80)	0.017^*^
Positive blood culture	16 (35.60)	7 (18.90)	0.095	10 (27.00)	7 (18.90)	0.407
Source of infection						
Lung infection	36 (80.00)	31 (83.80)	0.659	29 (78.40)	31 (83.80)	0.553
Digestive tract infection	3 (6.70)	1 (2.70)	0.407	2 (5.40)	1 (2.70)	0.556
Urinary infection	7 (15.60)	5 (13.50)	0.795	6 (16.20)	5 (13.50)	0.744
Skin or soft tissue infection	1 (2.20)	1 (2.70)	0.888	1 (2.70)	1 (2.70)	1.000
Underlying comorbidities						
Hypertension	18 (40.00)	20 (54.10)	0.204	17 (45.90)	20 (54.10)	0.485
Diabetes	13 (28.90)	14 (37.80)	0.391	11 (29.70)	14 (37.80)	0.461
Coronary heart disease	7 (15.60)	7 (18.90)	0.687	6 (16.20)	7 (18.90)	0.760
Stroke	5 (11.10)	9 (24.30)	0.114	5 (13.50)	9 (24.30)	0.235
Chronic obstructive pulmonary disease	5 (11.10)	6 (16.20)	0.500	4 (10.80)	6 (16.20)	0.496
Liver diseases	14 (31.10)	8 (21.60)	0.334	10 (27.00)	8 (21.60)	0.588
Kidney diseases	16 (35.60)	15 (40.50)	0.643	16 (43.20)	15 (40.50)	0.814
Before-treatment SOFA scores	6.00 (4.00-9.00)	6.00 (3.50-8.50)	0.826	6.00 (4.00-9.00)	6.00 (3.50-8.50)	0.896
Before-treatment APACHE II scores	37.00 (22.00-37.00)	37.00 (22.00-37.00)	0.509	37.00 (22.00-37.00)	37.00 (22.00-37.00)	0.685
Before-treatment procalcitonin	13.01 (3.01-35.48)	2.46 (0.77-19.01)	0.016^*^	13.01 (3.11-35.48)	2.46 (0.76-19.01)	0.022^*^
Before-treatment white blood cell count	16.35 (10.33-19.66)	11.76 (7.97-22.06)	0.476	16.87 (12.32-20.44)	11.76 (7.96-22.06)	0.202
Before-treatment blood lactic acid	2.80 (2.00-4.90)	2.60 (1.70-4.20)	0.404	3.00 (2.00-4.90)	2.60 (1.70-4.20)	0.333
Duration of taking YHJF	4.00 (2.00-6.50)	0.00 (0.00-0.00)	0.000^*^	4.00 (2.00-7.50)	0.00 (0.00-0.00)	0.000^*^

^*^ *p*<0.05. Normally distributed data were shown as mean ± standard deviation (mean ± SD), non-normally distributed data were presented with rank averages and distribution intervals [RA (minimum-maximum), and categorical data were expressed as frequency and percentage (n (%)]. YHJF, Yiqi Huoxue Jiedu Formula; PSM, Propensity score matching; BMI, Body Mass Index; SPO_2_, Peripheral Capillary Oxygen Saturation; SOFA, Sequential Organ Failure Assessment; APACHE II, Acute Physiology and Chronic Health Evaluation II.

**Table 4:** Comparative outcomes between YHJF and control groups in the septic shock cohort.

Outcomes	YHJF group *n* = (37)	Control group *n* = (37)	*p* valve
28-day mortality	6 (16.20)	10 (27.00)	0.259
Duration of ICU stays	4.00 (0.00-8.00)	10.00 (0.00-21.00)	0.071
Duration of hospitalization	9.00 (5.00-16.00)	18.00 (12.50-32.00)	0.000^*^
Length of noninvasive ventilation	0.00 (0.00-3.12)	0.58 (0.00-180.00)	0.016^*^
Length of invasive ventilation	0.00 (0.00-5.06)	5.00 (0.00-221.00)	0.024^*^
Length of CRRT	0.00 (0.00-0.00)	0.00 (0.00-0.00)	0.434
Duration of fever	3.00 (1.50-5.50)	4.00 (0.00-11.50)	0.828
Day 5 SOFA scores	2.00 (1.00-3.00)	2.00 (1.00-3.00)	0.361
Day 5 APACHE II scores	17.97 ± 3.55	17.00 ± 4.08	0.277
Day 5 procalcitonin	1.38 (0.84-9.06)	1.42 (0.55-5.81)	0.443
Day 5 white blood cell count	9.08 (5.39-13.75)	9.50 (7.59-12.06)	0.791
Day 5 blood lactic acid	1.60 (1.15-1.95)	1.90 (1.45-2.55)	0.043^*^

^*^ *p*<0.05. Normally distributed data were shown as mean ± standard deviation (mean ± SD), non-normally distributed data were presented with rank averages and distribution intervals [RA (minimum-maximum), and categorical data were expressed as frequency and percentage (n (%)]. YHJF, Yiqi Huoxue Jiedu Formula; ICU, Intensive care unit; CRRT, Continuous renal replacement therapy; SOFA, Sequential Organ Failure Assessment; APACHE II, Acute Physiology and Chronic Health Evaluation II.

### Retrospective cohort study

**Figure 2:**
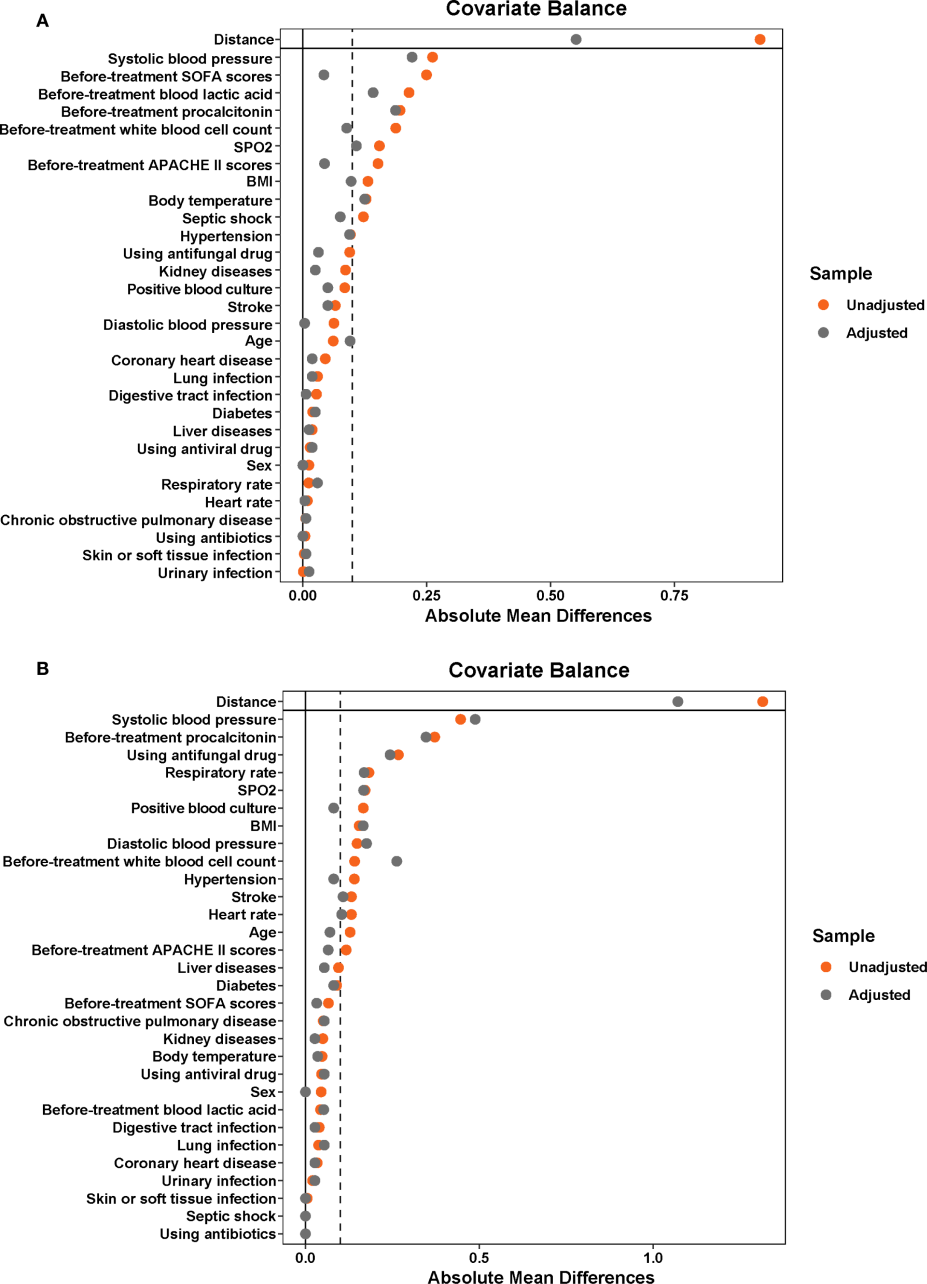
The distribution of baseline characteristics before and after PSM in the sepsis and septic shock cohorts. Before PSM, the YHJF group included 159 patients, while the control group comprised 230 patients. Following PSM, both groups were balanced to include 159 patients each. The closer the data point is to the zero line, the more balanced the corresponding baseline data becomes. **(A)** sepsis cohorts; **(B)** septic shock cohorts. YHJF, Yiqi Huoxue Jiedu Formula; PSM, Propensity score matching; BMI, Body Mass Index; SPO2, Peripheral Capillary Oxygen Saturation; SOFA, Sequential Organ Failure Assessment; APACHE II, Acute Physiology and Chronic Health Evaluation II.

**Figure 3:**
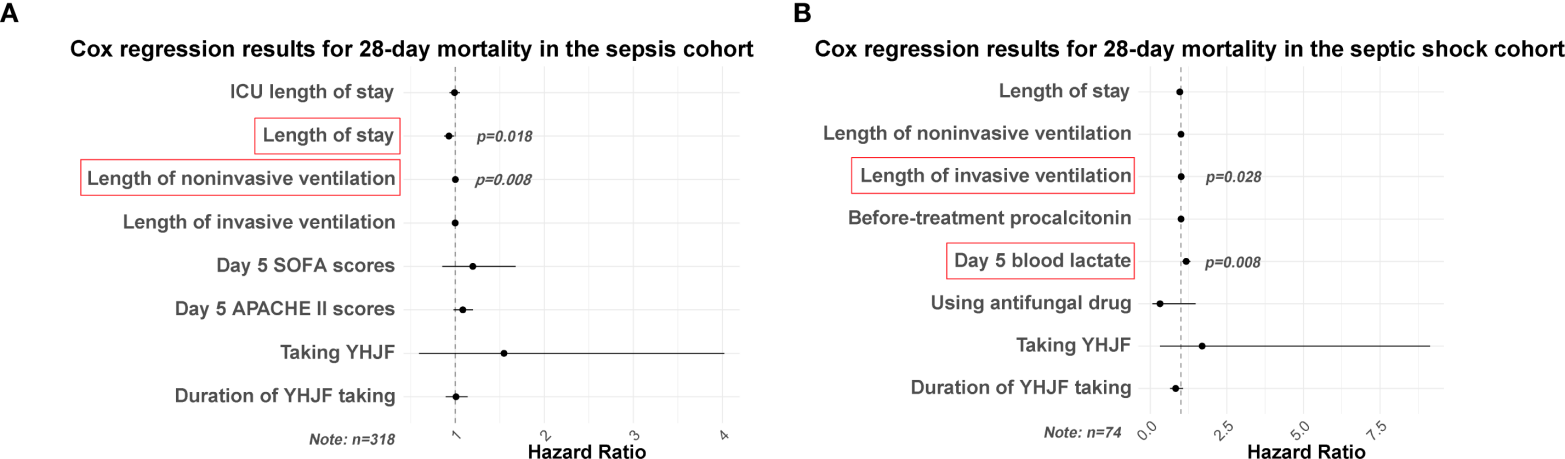
Cox regression results for 28-day mortality in the sepsis and septic shock cohorts. Statistically significant results highlighted in red. **(A)** Cox regression results for 28-day mortality in the sepsis cohort (YHJF group: n=159; control group: n=159); **(B)** Cox regression results for 28-day mortality in the septic shock cohort (YHJF group: n=37; control group: n=37). YHJF, Yiqi Huoxue Jiedu Formula; ICU, Intensive care unit; SOFA, Sequential Organ Failure Assessment; APACHE II, Acute Physiology and Chronic Health Evaluation II.

After screening, 159 patients were allocated to the YHJF group and 230 patients were assigned to the control group (**Figure 2**). The retrospective results revealed statistically significant in baseline data including BMI, systolic blood pressure, and initial body temperature between the two groups. (**Table 1**). To address these disparities, a 1:1 nearest neighbor matching procedure was employed, resulting in 159 matched patients in each group. Post-matching, the comparability between the two groups was improved (**Table 1**; **Figure 3**).

**Figure 4:**
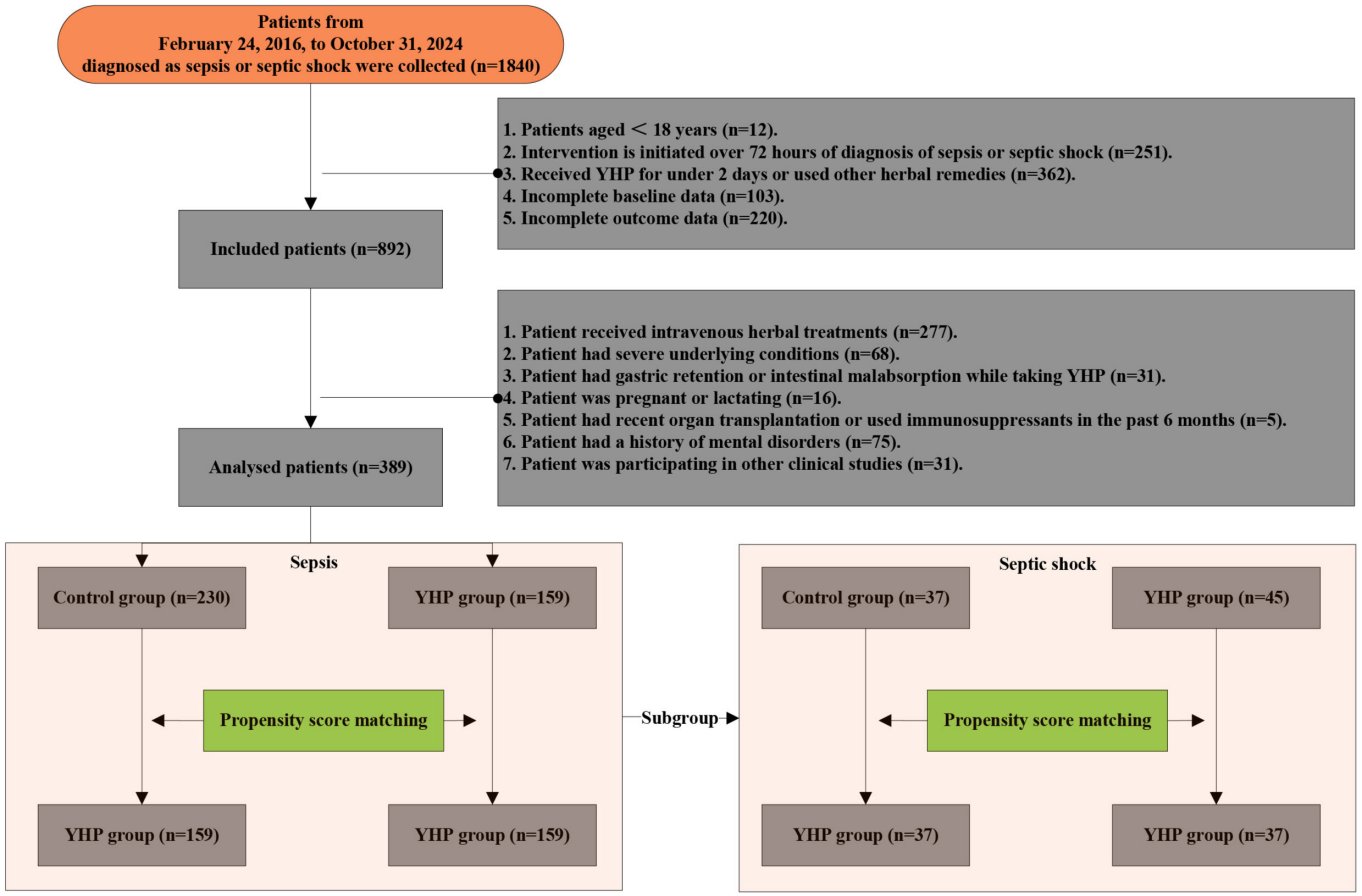
Flowchart for the retrospective cohort study.

The intergroup comparisons demonstrated no significant difference (*p* > 0.05) for 28-day mortality. Furthermore, in the Cox regression analysis with 28-day mortality as the dependent variable, neither the utilization of YHJF nor the duration of its use exhibited statistical significance after adjusting for confounding variables (*p* > 0.05). Shorter hospital stays and a prolonged duration of invasive ventilation were identified as risk factors for 28-day mortality in sepsis patients (**Figure 4**).

**Figure 5:**
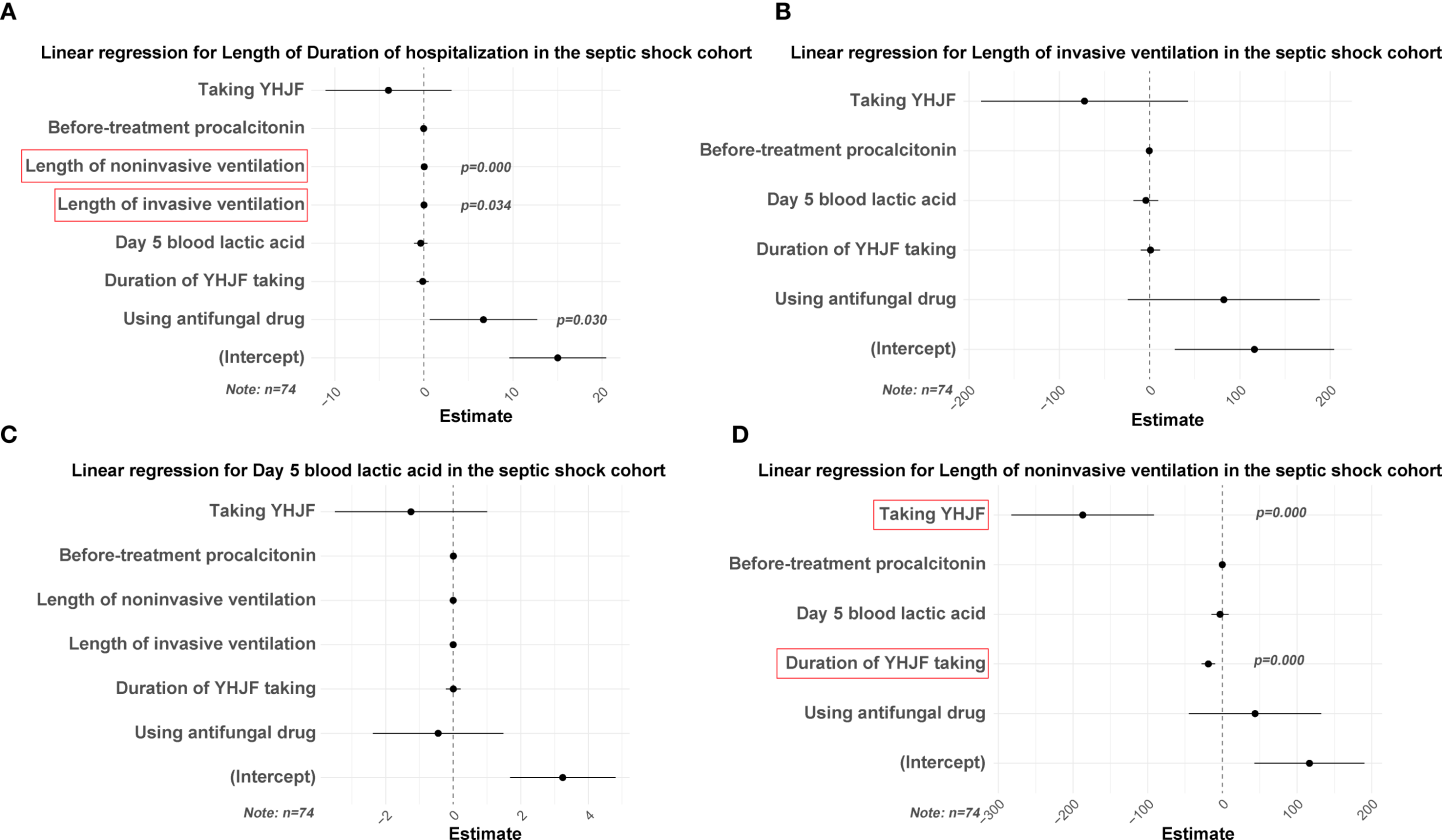
Linear regression results for the YHJF administration in the septic shock cohort. Statistically significant secondary outcomes from the univariate analysis were used as dependent variables. After adjusting for confounders, the effect of YHJF on these variables was assessed, with significant results highlighted in red. YHJF group: n=37; control group: n=37. **(A)** linear regression results for duration of hospitalization; **(B)** linear regression results for length of invasive ventilation; **(C)** linear regression results for day 5 blood lactic acid; **(D)** linear regression results for length of noninvasive ventilation. YHJF, Yiqi Huoxue Jiedu Formula.

Intergroup comparisons of secondary outcomes indicated that YHJF significantly reduced ICU and overall hospital stays for sepsis patients, decreased the duration of both invasive and non-invasive ventilator use, and resulted in lower SOFA and APACHE II scores (**Table 2**). Furthermore, these statistically significant outcomes were considered as the dependent variable in the linear regression model. Independent variables potentially influencing their changes were incorporated to assess the impact of YHJF application on each outcome. The findings indicated that YHJF administration significantly reduced hospital stay duration, decreased the duration of both non-invasive and invasive ventilator use, and improved the SOFA score in sepsis patients (**Figure 5**).

**Figure 6:**
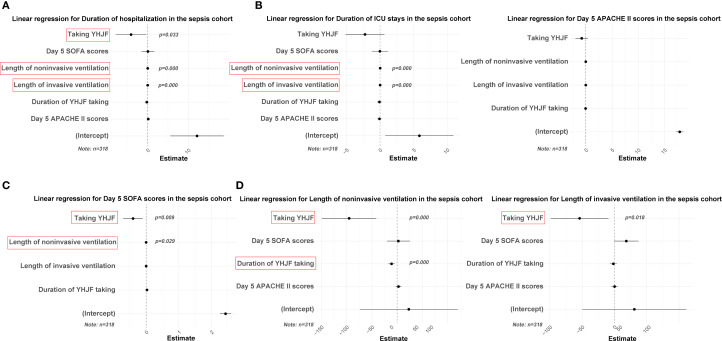
Linear regression results for the YHJF administration in the sepsis cohort. Statistically significant secondary outcomes from the univariate analysis were used as dependent variables. After adjusting for confounders, the effect of YHJF on these variables was assessed, with significant results highlighted in red. YHJF group: n=159; control group: n=159. **(A)** linear regression results for duration of hospitalization; **(B)** linear regression results for duration of lCU stays; **(C)** linear regression results for APACHE II scores; **(D)** linear regression results for SOFA scores; **(E)** linear regression results for length of noninvasive ventilation; **(F)** linear regression results for length of invasive ventilation. YHJF, Yiqi Huoxue Jiedu Formula; ICU, Intensive care unit; SOFA, Sequential Organ Failure Assessment; APACHE II, Acute Physiology and Chronic Health Evaluation II.

To further assess the therapeutic efficacy of YHJF in septic shock, we performed a subgroup analysis. The analysis included 45 patients in the YHJF group and 37 patients in the control group. Utilizing 1:1 nearest neighbor matching, 37 patients were successfully matched in each group (**Table 3**; **Figure 1**), resulting in a more balanced baseline between the two groups (**Table 3**). A univariate analysis indicated that the YHJF administration may reduce the length of hospital stay, decrease the duration of both invasive and non-invasive ventilator use, and lower blood lactate levels (**Table 4**). However, after controlling confounding variables, the analysis revealed that YHJF was significantly associated only with an improvement in the duration of non-invasive ventilator use among septic shock patients (**Figure 6**). Notably, both intergroup comparisons and Cox regression analyses indicated that YHJF administration did not statistically influence the 28-day mortality (*p* > 0.05) (**Table 4**; **Figure 4**). Instead, prolonged duration of non-invasive ventilation and elevated lactate levels after 5 days of treatment emerged as risk factors for 28-day mortality in septic shock patients (**Figure 4**).

## Discussion

YHJF was initially formulated as an herbal compound grounded in the theory of traditional Chinese medicine (TCM). Within the TCM theoretical framework, sepsis is understood to encompass multiple syndromes, including “qi stagnation and blood stasis”, “heat-toxin congestion”, “phlegm-heat entrapment”, and “deficiency of vital energy”. We conducted two large-scale retrospective studies to summary the core pathogenesis of sepsis according to TCM theory, identifying it as “acute deficiency syndrome (Xiao et al., 2016; Zhou et al., 2021).” This concept suggests that during the early stages of sepsis, pathogenic factors such as “toxin” and “blood stasis” interact with the body, subsequently leading to manifestations of “vital energy deficiency”. Based on these findings, it is crucial to not only eliminate pathogenic factors like “toxin” and “blood stasis” but also to initiate the protection of vital energy as early as possible. Guided by the central theory of “strengthening the body and eliminating toxins,” YHJF was developed as a specialized and efficacious herbal compound in treating sepsis through the careful selection of various herbal medicines. In the compound, Rhubarb is a bowel-clearing and detoxifying Chinese herb, Sanchi can promote blood circulation, remove blood stasis and replenish deficiency, and Ginseng Root can protect vital energy.

Our previous research has demonstrated that YHJF can enhance the 7-day survival rate in septic mice, mitigate apoptosis in splenic cells, increase the spleen index, and decrease the levels of IL-4 and IL-10 in the peripheral blood (Chen et al.). The present study indicates that YHJF does not confer therapeutic benefits for the 28-day mortality in sepsis and septic shock. Nonetheless, The current research demonstrates that YHJF confers improvements in some secondary outcomes associated with sepsis and septic shock, highlighting its potential as an adjunctive therapeutic strategy. This raises the question: what mechanisms underlie the therapeutic effects of YHJF?

Dissecting the mechanism of action of each herbal constituent in YHJF is the primary task in explaining its clinical efficacy. Based on published works, the herbal constituents of YHJF exhibit multifaceted roles in managing sepsis. For example, Rhubarb contributes to the amelioration of various pathological processes linked to sepsis, including the modulation of intestinal flora imbalance, enhancement of intestinal mucosal barrier function, inhibition of oxidative stress-induced damage, promotion of microcirculatory blood flow in the small intestine, suppression of excessive platelet activation, and inhibition of apoptosis (Yang et al., 2010; Wei and Xie, 2011; Dai et al., 2023; Ou et al., 2023). Ginseng Root has been shown to enhance the immune function in sepsis patients by modulating cytokine production and promoting immune cell activity (Liu et al., 2021). It also neutralizes reactive oxygen species (ROS), thereby protecting cellular components from oxidative damage and maintaining cell integrity under septic conditions (Park et al., 2021). Additionally, Ginseng Root inhibits inflammatory pathways, such as the MAPKs/NF-κB pathway, which reduces the production of pro-inflammatory cytokines and mitigates the systemic inflammatory response characteristic of sepsis (Lee et al., 2018). As for Sanchi, it has been shown to inhibit inflammatory pathways and reduce the production of inflammatory cytokines (Jiao et al., 2021). By enhancing lymphatic function, Sanchi facilitates the clearance of inflammatory mediators, thereby mitigating the systemic inflammatory response (Jiao et al., 2021). In addition, components extracted from Sanchi have been observed to influence the actin cytoskeleton in endothelial cells, potentially stabilizing the vascular endothelium and preventing leakage (Wang et al., 2020). Notably, Xuebijing injection, prepared using extracts from Sanchi and other herbal medicines, has been demonstrated to improve sepsis prognosis through mechanisms involving multiple targets and pathways (Li et al., 2021; Liu et al., 2023).

However, rigorously speaking, the constituents of each herb do not precisely correspond to those of the combined formula. This discrepancy arises because the processing involved in combined drug manufacture can alter certain components (Jin et al., 2016; Yang et al., 2018). On the other hand, the process of biological absorption may selectively filter out many components (Liu et al., 2014). Consequently, greater attention should be directed towards the absorbed components. To gain a clearer understanding of the components absorbed into the living organism following YHJF administration, we analyzed the drug-containing serum in rats using UHPLC-HRMS. The findings indicate that the primary absorbed components of YHJF include Shikimates and Phenylpropanoids, Terpenoids, Alkaloids, and Polyketides. Pioneering studies show that Gallic acid and Protocatechuic acid, representative compounds within the Shikimates, along with Flavonoids, Coumarins, and Lignans found in Phenylpropanoids, exhibit notable antioxidant and anti-inflammatory properties (Cheng et al., 2024; Li et al., 2024; Slim et al., 2024). Hederagenin derivatives and Oleanane triterpenoid saponins, categorized under Terpenoids, demonstrate their efficacy by modulating key pathological processes in sepsis, including inflammation, oxidative stress, and immune dysregulation (Xie et al., 2024; Xie et al., 2025). Berberine and Matrine, representative alkaloids, are known for their anti-inflammatory, immune-regulatory, and intestinal barrier protective effects (Wang and Ma, 2020; Sun et al., 2023). Additionally, Polyketides such as Sparassis latifolia polysaccharide and Hydroxysafflor Yellow A can mitigate oxidative stress, safeguarding mitochondrial function, and ameliorating microcirculation disturbances (Hu et al., 2016; Lu et al., 2024; Wu et al., 2024).

Apart from the mechanism inferred based on these identified chemical substances, our prior research elucidated a precise mechanism by which YHJF ameliorates sepsis based on transcriptomics and metabolomics. Specifically, the intervention with YHJF resulted in the downregulation of the negative co-stimulatory molecule CD160 on natural killer T (NKT) cells within the spleen of septic mice, thereby mitigating NKT cell exhaustion associated with sepsis (Chen et al.). Previous research has demonstrated that terpenoids, alkaloids, and polyketides encompass various compounds capable of modulating the activity of NKT cells, including sesquiterpene lactones such as parthenolide and estafiatin, pentacyclic triterpenoids, and piperlongumine (Markov et al., 2017; Schepetkin et al., 2018; Khan et al., 2019; Afolabi et al., 2021). However, the precise compounds within YHJF that target CD160 on NKT cells to prevent their exhaustion and improve immune dysregulation in sepsis have yet to be identified. This represents a critical avenue for future experimental investigation.

While we have enumerated a series of studies investigating the ameliorative effects of YHJF on certain secondary outcomes, the lack of impact on 28-day mortality remains a critical consideration in interpreting the findings of this study. Typically, pharmacological agents that influence disease mortality are characterized by potent pharmacological actions or require long-term intervention to produce cumulative effects. The YHJF administration in this study predominantly spanned a duration of one week. The limited efficacy observed may be attributable to the short-term nature of this pharmacological intervention. Furthermore, as previously discussed, the components of YHJF are primarily associated with the modulation of inflammation and immune responses. Although short-term alterations in these pathological mechanisms may contribute to the improvements in clinical symptoms and patient physiological functions, they are insufficient to affect a reversal in mortality. Finally, in severe pathological conditions, mortality is influenced by a multitude of factors, such as pathogen type, the patient’s pre-existing health status, and the accuracy of anti-infective treatments. Even after conducting a correction analysis, these factors exert a substantial impact on mortality, rendering the effects of YHJF comparatively negligible. Consequently, until these concerns are addressed, the potential impact of YHJF on reducing 28-day mortality in these patients remains unsubstantiated. The clinical application of YHJF as an adjunctive therapy for sepsis or septic shock should be approached with heightened caution.

Overall, this study substantiates the beneficial impact of YHJF on certain outcomes associated with sepsis and septic shock. However, due to the partial limitations, these findings should be interpreted with caution. Firstly, the study’s design as a retrospective cohort with a limited sample size inherently results in a lower level of evidence. Secondly, in the septic shock subgroup, even after performing PSM, significant differences persisted in baseline data, such as “use of antifungal drugs” and “procalcitonin levels before treatment,” which could compromise the stability of subsequent analyses. Furthermore, the conclusion that CD160 alleviates NKT cell exhaustion and its association with inflammation regulation for the treatment of YHJF in sepsis is currently supported only by animal experimental evidence and requires further validation through clinical trials. Additionally, while drug-containing serum from animals has been employed in preliminary pharmacological and pharmacokinetic studies of various Chinese herbal medicines (Zhou et al., 2018; Gu et al., 2022), discrepancies in pharmacokinetics, dose-response relationships, and other factors between rodents and humans necessitate further confirmation of human pharmacokinetic trials. Finally, although the absorbed compounds identified in this study may have therapeutic effects in these conditions, the proposed “components - pharmacological mechanism - clinical efficacy” framework remains speculative. Future research should focus on selecting representative chemical monomers from YHJF for mechanistic studies and efficacy correlation to elucidate the biological mechanisms of YHJF more clearly. Overall, it is important to acknowledge the inherent limitations of this study when interpreting its findings. Nonetheless, the results possess clinical significance and, crucially, provide a foundation for future research directions.

## Conclusion

As an adjunctive therapeutic strategy, YHJF could reduce the SOFA score, ventilator use duration, and hospital stay length in sepsis cases. Additionally, it could enhance the duration of non-invasive ventilator use in septic shock patients. These clinical effects may be attributed to the presence of absorbed components following YHJF administration. Nevertheless, additional high-quality clinical and fundamental research is required to validate and elucidate the current findings.

## Data Availability

The original contributions presented in the study are included in the article/**Supplementary Material**. Further inquiries can be directed to the corresponding authors.
