# Supplementary material for: Uncovering the potential of Yiqi Huoxue Jiedu formula: a promising adjunct in sepsis and septic shock management
Source: Front Cell Infect Microbiol. 2025 Oct 15;15:1655393. doi: 10.3389/fcimb.2025.1655393 (PMC12568585; doi:10.3389/fcimb.2025.1655393)
Supplement: Supplementary file 1 [file Table1.docx]

**Content**

[**Supplementary Figure 1. The NPClassifier classification results for all the identified compounds. 2**](#_Toc194248758)

[**Supplementary Table 1. The NPClassifier classification results for all the identified compounds. 2**](#_Toc194248759)

[**Supplementary Figure 2. The NPClassifier classification results for the compounds detected in the serum. 12**](#_Toc194248760)

[**Supplementary Table 2. The NPClassifier classification results for the compounds detected in the serum. 12**](#_Toc194248761)

[**upplementary Figure 3. The BPC results in cation analysis for the following groups: the aqueous extract group of YHP, the blank serum group combined with the aqueous extract group of YHP, the blank serum group, and the YHP drug-containing serum group. These groups are presented sequentially from top to bottom in the order listed. 15**](#_Toc194248762)

[**Supplementary Figure 4. The BPC results in anion analysis for the following groups: the aqueous extract group of YHP, the blank serum group combined with the aqueous extract group of YHP, the blank serum group, and the YHP drug-containing serum group. These groups are presented sequentially from top to bottom in the order listed. 16**](#_Toc194248763)

[**Supplementary Table 3. Chemical components identified in YHP aqueous extract and YHP-medicated serum using UHPLC-HRMS in cation analysis. 17**](#_Toc194248764)

[**Supplementary Table 4. Chemical components identified in YHP aqueous extract and YHP-medicated serum using UHPLC-HRMS in anion analysis. 52**](#_Toc194248765)

# Supplementary Figure 1. The NPClassifier classification results for all the identified compounds.


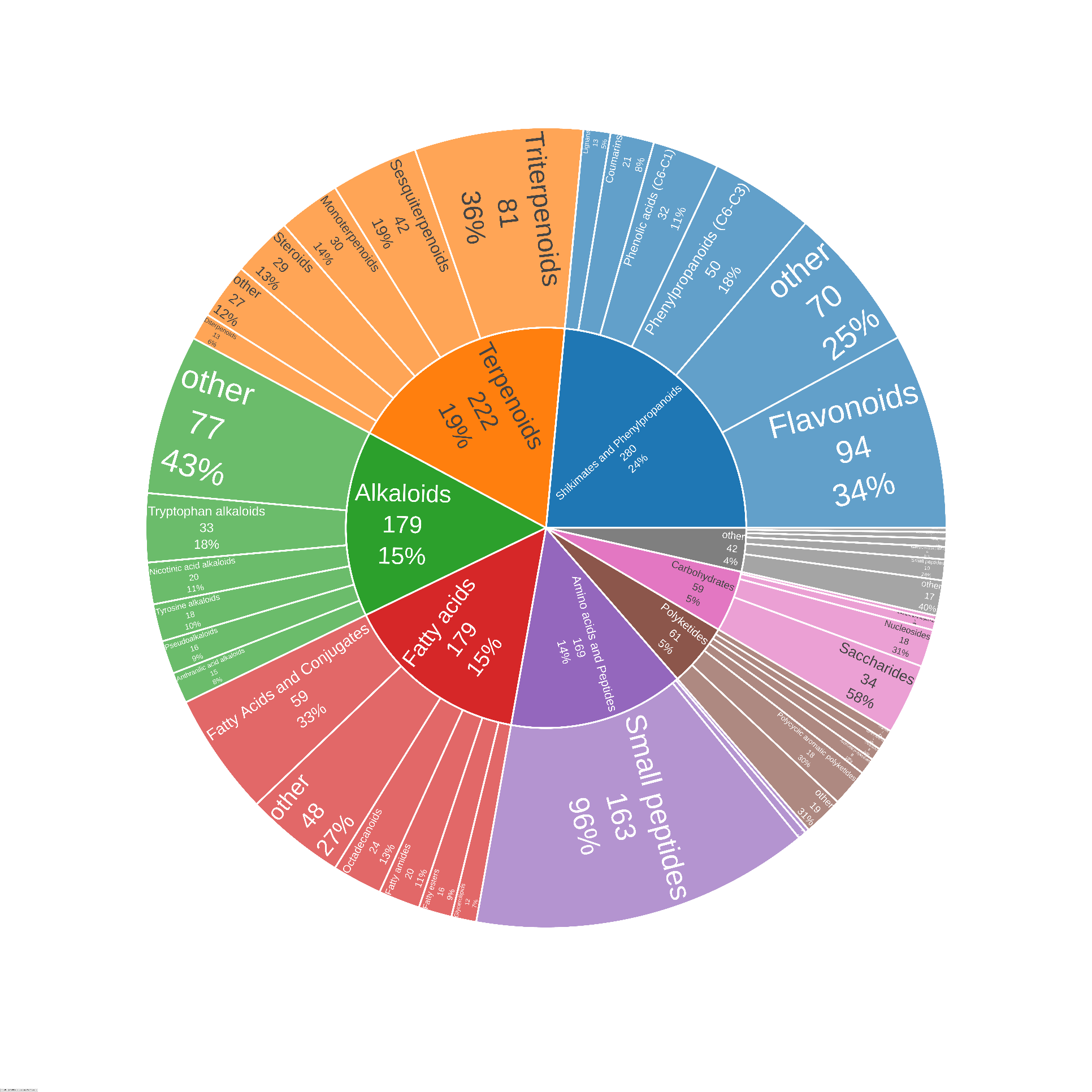


# Supplementary Table 1. The NPClassifier classification results for all the identified compounds.

| Pathway | SuperClass | Class | count |
| --- | --- | --- | --- |
| Alkaloids | Anthranilic acid alkaloids | Acridone alkaloids | 4 |
| Alkaloids | Anthranilic acid alkaloids | Anthranillic acid derivatives | 2 |
| Alkaloids | Anthranilic acid alkaloids | Benzodiazepine alkaloids | 1 |
| Alkaloids | Anthranilic acid alkaloids | Phenazine alkaloids | 1 |
| Alkaloids | Anthranilic acid alkaloids | Phenoxazine alkaloids | 1 |
| Alkaloids | Anthranilic acid alkaloids | Quinazoline alkaloids | 1 |
| Alkaloids | Anthranilic acid alkaloids | Quinoline alkaloids | 3 |
| Alkaloids | Anthranilic acid alkaloids | other | 2 |
| Alkaloids | Histidine alkaloids | Imidazole alkaloids | 2 |
| Alkaloids | Lysine alkaloids | Piperidine alkaloids | 7 |
| Alkaloids | Lysine alkaloids | other | 3 |
| Alkaloids | Lysine alkaloids+Nicotinic acid alkaloids | Pyridine alkaloids+Quinolizidine alkaloids | 1 |
| Alkaloids | Nicotinic acid alkaloids | Pyridine alkaloids | 20 |
| Alkaloids | Ornithine alkaloids | Polyamines | 2 |
| Alkaloids | Ornithine alkaloids | Pyrrolidine alkaloids | 1 |
| Alkaloids | Ornithine alkaloids | Pyrrolizidine alkaloids | 1 |
| Alkaloids | Ornithine alkaloids | Stemona alkaloids | 1 |
| Alkaloids | Ornithine alkaloids | Tropane alkaloids | 2 |
| Alkaloids | Ornithine alkaloids | other | 1 |
| Alkaloids | Pseudoalkaloids | Acetate-derived alkaloids | 1 |
| Alkaloids | Pseudoalkaloids | Phenylalanine-derived alkaloids | 7 |
| Alkaloids | Pseudoalkaloids | Purine alkaloids | 5 |
| Alkaloids | Pseudoalkaloids | other | 2 |
| Alkaloids | Pseudoalkaloids | pteridine alkaloids | 1 |
| Alkaloids | Tryptophan alkaloids | Aspidosperma type+Aspidosperma-Iboga hybrid type (Vinca alkaloids) | 1 |
| Alkaloids | Tryptophan alkaloids | Carbazole alkaloids | 1 |
| Alkaloids | Tryptophan alkaloids | Carboline alkaloids | 6 |
| Alkaloids | Tryptophan alkaloids | Simple indole alkaloids | 19 |
| Alkaloids | Tryptophan alkaloids | Simple indole alkaloids+Simple oxindole alkaloids | 1 |
| Alkaloids | Tryptophan alkaloids | Simple oxindole alkaloids | 1 |
| Alkaloids | Tryptophan alkaloids | other | 4 |
| Alkaloids | Tryptophan alkaloids+Anthranilic acid alkaloids | Quinoline alkaloids | 2 |
| Alkaloids | Tyrosine alkaloids | Amarylidaceae alkaloids | 2 |
| Alkaloids | Tyrosine alkaloids | Aporphine alkaloids+Isoquinoline alkaloids | 1 |
| Alkaloids | Tyrosine alkaloids | Isoquinoline alkaloids | 1 |
| Alkaloids | Tyrosine alkaloids | Isoquinoline alkaloids+Morphinan alkaloids | 1 |
| Alkaloids | Tyrosine alkaloids | Isoquinoline alkaloids+Protoberberine alkaloids | 3 |
| Alkaloids | Tyrosine alkaloids | Phenylethylamines | 6 |
| Alkaloids | Tyrosine alkaloids | Protoberberine alkaloids | 2 |
| Alkaloids | Tyrosine alkaloids | other | 2 |
| Alkaloids | other | other | 54 |
| Alkaloids+Amino acids and Peptides | Lysine alkaloids+Small peptides | other | 1 |
| Alkaloids+Amino acids and Peptides | Peptide alkaloids+Small peptides | Dipeptides+Indole diketopiperazine alkaloids (L-Trp, L-Pro) | 1 |
| Alkaloids+Amino acids and Peptides | Peptide alkaloids+Small peptides | Dipeptides+Simple diketopiperazine alkaloids | 1 |
| Alkaloids+Amino acids and Peptides | Tetramate alkaloids+Peptide alkaloids | Pyrazine and Piperazine alkaloids | 1 |
| Alkaloids+Amino acids and Peptides | other | other | 2 |
| Alkaloids+Fatty acids | other | other | 1 |
| Alkaloids+Shikimates and Phenylpropanoids | Tryptophan alkaloids+Phenylpropanoids (C6-C3) | Cinnamic acids and derivatives+Simple indole alkaloids | 1 |
| Alkaloids+Shikimates and Phenylpropanoids | other | other | 4 |
| Alkaloids+Terpenoids | Pseudoalkaloids | Steroidal alkaloids | 1 |
| Amino acids and Peptides | Oligopeptides | Cyclic peptides | 2 |
| Amino acids and Peptides | Small peptides | Aminoacids | 52 |
| Amino acids and Peptides | Small peptides | Aminoacids+Dipeptides | 6 |
| Amino acids and Peptides | Small peptides | Dipeptides | 87 |
| Amino acids and Peptides | Small peptides | Dipeptides+Tripeptides | 6 |
| Amino acids and Peptides | Small peptides | Tripeptides | 10 |
| Amino acids and Peptides | Small peptides | other | 2 |
| Amino acids and Peptides | other | other | 4 |
| Amino acids and Peptides+Fatty acids | Fatty Acids and Conjugates | Amino fatty acids | 1 |
| Amino acids and Peptides+Fatty acids | Fatty Acids and Conjugates+Small peptides | Amino fatty acids+Aminoacids | 2 |
| Amino acids and Peptides+Fatty acids | Fatty Acids and Conjugates+Small peptides | Aminoacids+Oxo fatty acids | 1 |
| Amino acids and Peptides+Polyketides | Macrolides+Cyclic polyketides | 3-Spirotetramic acids+Erythromycins | 1 |
| Amino acids and Peptides+Polyketides | Oligopeptides | Cyclic peptides+Depsipeptides | 1 |
| Amino acids and Peptides+Shikimates and Phenylpropanoids | Phenylpropanoids (C6-C3) | Cinnamic acid amides | 6 |
| Amino acids and Peptides+Shikimates and Phenylpropanoids | Small peptides | Aminoacids | 10 |
| Carbohydrates | Aminosugars and aminoglycosides | Aminosugars | 5 |
| Carbohydrates | Nucleosides | Purine nucleos(t)ides | 9 |
| Carbohydrates | Nucleosides | Pyrimidine nucleos(t)ides | 9 |
| Carbohydrates | Polyols | Cyclitols | 2 |
| Carbohydrates | Saccharides | Disaccharides | 10 |
| Carbohydrates | Saccharides | Disaccharides+Polysaccharides | 7 |
| Carbohydrates | Saccharides | Monosaccharides | 14 |
| Carbohydrates | Saccharides | Polysaccharides | 3 |
| Carbohydrates+Shikimates and Phenylpropanoids | Saccharides+Flavonoids | Disaccharides+Flavones+Monosaccharides+Polysaccharides | 1 |
| Fatty acids | Docosanoids | Other Docosanoids | 3 |
| Fatty acids | Eicosanoids | Other Eicosanoids | 2 |
| Fatty acids | Eicosanoids+Fatty Acids and Conjugates | Hydroxy fatty acids+Hydroxy-hydroperoxyeicosatetraenoic acids+Hydroxy-hydroperoxyeicosatrienoic acids | 1 |
| Fatty acids | Fatty Acids and Conjugates | Branched fatty acids | 3 |
| Fatty acids | Fatty Acids and Conjugates | Branched fatty acids+Hydroxy fatty acids | 1 |
| Fatty acids | Fatty Acids and Conjugates | Branched fatty acids+Oxo fatty acids | 1 |
| Fatty acids | Fatty Acids and Conjugates | Dicarboxylic acids | 22 |
| Fatty acids | Fatty Acids and Conjugates | Dicarboxylic acids+Hydroxy fatty acids | 3 |
| Fatty acids | Fatty Acids and Conjugates | Dicarboxylic acids+Oxo fatty acids | 1 |
| Fatty acids | Fatty Acids and Conjugates | Heterocyclic fatty acids | 1 |
| Fatty acids | Fatty Acids and Conjugates | Hydroxy fatty acids | 10 |
| Fatty acids | Fatty Acids and Conjugates | Hydroxy fatty acids+Unsaturated fatty acids | 1 |
| Fatty acids | Fatty Acids and Conjugates | Oxo fatty acids | 4 |
| Fatty acids | Fatty Acids and Conjugates | Unsaturated fatty acids | 11 |
| Fatty acids | Fatty Acids and Conjugates | other | 1 |
| Fatty acids | Fatty Acids and Conjugates+Octadecanoids | Hydroxy fatty acids+Other Octadecanoids | 1 |
| Fatty acids | Fatty Acids and Conjugates+Octadecanoids | Other Octadecanoids+Oxo fatty acids | 1 |
| Fatty acids | Fatty Acids and Conjugates+Octadecanoids | Other Octadecanoids+Oxo fatty acids+Unsaturated fatty acids | 2 |
| Fatty acids | Fatty acyl glycosides | Fatty acyl glycosides of mono- and disaccharides | 4 |
| Fatty acids | Fatty acyls | Fatty alcohols | 1 |
| Fatty acids | Fatty acyls | Fatty aldehydes | 2 |
| Fatty acids | Fatty acyls | Hydrocarbons | 6 |
| Fatty acids | Fatty acyls | Paraconic acids and derivatives | 1 |
| Fatty acids | Fatty amides | N-acyl amines | 12 |
| Fatty acids | Fatty amides | N-acyl ethanolamines (endocannabinoids) | 5 |
| Fatty acids | Fatty amides | Primary amides | 3 |
| Fatty acids | Fatty esters | Fatty acyl carnitines | 6 |
| Fatty acids | Fatty esters | Lactones | 4 |
| Fatty acids | Fatty esters | Wax monoesters | 6 |
| Fatty acids | Glycerolipids | Diacylglycerols | 2 |
| Fatty acids | Glycerolipids | Glycosylmonoacylglycerols | 3 |
| Fatty acids | Glycerolipids | Monoacylglycerols | 6 |
| Fatty acids | Glycerolipids | Triacylglycerols | 1 |
| Fatty acids | Glycerolipids+Fatty esters | Monoacylglycerols+Wax monoesters | 1 |
| Fatty acids | Glycerophospholipids | Glycerophosphocholines | 6 |
| Fatty acids | Glycerophospholipids | Glycerophosphoglycerols | 1 |
| Fatty acids | Glycerophospholipids | other | 2 |
| Fatty acids | Octadecanoids | Jasmonic acids | 5 |
| Fatty acids | Octadecanoids | Other Octadecanoids | 17 |
| Fatty acids | Octadecanoids | other | 2 |
| Fatty acids | Sphingolipids | Ceramides | 4 |
| Fatty acids | Sphingolipids | Neutral glycosphingolipids+Sphingoid bases | 1 |
| Fatty acids | Sphingolipids | Sphingoid bases | 3 |
| Fatty acids | other | other | 6 |
| Polyketides | Aromatic polyketides | Aromatic polyketides with side chains | 1 |
| Polyketides | Aromatic polyketides | Catechols with side chains | 3 |
| Polyketides | Aromatic polyketides | Depsides | 2 |
| Polyketides | Aromatic polyketides | Depsidones | 2 |
| Polyketides | Chromanes | Azaphilones | 1 |
| Polyketides | Chromanes | Chromones | 5 |
| Polyketides | Cyclic polyketides | 4-pyrone derivatives | 1 |
| Polyketides | Cyclic polyketides | Furans | 1 |
| Polyketides | Cyclic polyketides | Monacolins and Monacolin derivatives | 1 |
| Polyketides | Cyclic polyketides | Phthalide derivatives | 2 |
| Polyketides | Diphenyl ethers (DPEs) | Fungal DPEs | 1 |
| Polyketides | Linear polyketides | Open-chain polyketides | 3 |
| Polyketides | Linear polyketides | Polyesters | 1 |
| Polyketides | Macrolides | Avermectins | 1 |
| Polyketides | Macrolides | Erythromycins | 1 |
| Polyketides | Macrolides | Macrolide lactones | 2 |
| Polyketides | Macrolides | Zearalenones | 1 |
| Polyketides | Naphthalenes | Naphthalenes and derivatives | 2 |
| Polyketides | Naphthalenes | Naphthalenes and derivatives+Naphthoquinones | 1 |
| Polyketides | Naphthalenes | Naphthalenones | 1 |
| Polyketides | Phloroglucinols | Acyl phloroglucinols | 1 |
| Polyketides | Phloroglucinols | Dimeric phloroglucinols | 1 |
| Polyketides | Polycyclic aromatic polyketides | Angucyclines | 1 |
| Polyketides | Polycyclic aromatic polyketides | Anthraquinones and anthrones | 17 |
| Polyketides | Xanthones | Methyl xanthones | 1 |
| Polyketides | other | other | 7 |
| Polyketides+Terpenoids | Meroterpenoids | Cannabinoids | 2 |
| Polyketides+Terpenoids | Meroterpenoids | Tetraketide meroterpenoids | 1 |
| Polyketides+Terpenoids | Meroterpenoids | other | 1 |
| Polyketides+Terpenoids | Naphthalenes+Diterpenoids | Abietane diterpenoids+Furanoabietane diterpenoids+Naphthoquinones | 1 |
| Shikimates and Phenylpropanoids | Coumarins | Isocoumarins | 6 |
| Shikimates and Phenylpropanoids | Coumarins | Simple coumarins | 14 |
| Shikimates and Phenylpropanoids | Coumarins | other | 1 |
| Shikimates and Phenylpropanoids | Diarylheptanoids | Linear diarylheptanoids | 2 |
| Shikimates and Phenylpropanoids | Flavonoids | Anthocyanidins | 2 |
| Shikimates and Phenylpropanoids | Flavonoids | Chalcones | 9 |
| Shikimates and Phenylpropanoids | Flavonoids | Dihydroflavonols | 1 |
| Shikimates and Phenylpropanoids | Flavonoids | Dihydroflavonols+Flavanones | 1 |
| Shikimates and Phenylpropanoids | Flavonoids | Flavan-3-ols | 11 |
| Shikimates and Phenylpropanoids | Flavonoids | Flavanones | 12 |
| Shikimates and Phenylpropanoids | Flavonoids | Flavones | 23 |
| Shikimates and Phenylpropanoids | Flavonoids | Flavonols | 34 |
| Shikimates and Phenylpropanoids | Flavonoids | Proanthocyanins | 1 |
| Shikimates and Phenylpropanoids | Isoflavonoids | Isoflavanones | 1 |
| Shikimates and Phenylpropanoids | Isoflavonoids | Isoflavones | 7 |
| Shikimates and Phenylpropanoids | Isoflavonoids | Pterocarpan | 1 |
| Shikimates and Phenylpropanoids | Isoflavonoids | Rotenoids | 1 |
| Shikimates and Phenylpropanoids | Lignans | Arylnaphthalene and aryltetralin lignans | 2 |
| Shikimates and Phenylpropanoids | Lignans | Dibenzylbutane lignans | 2 |
| Shikimates and Phenylpropanoids | Lignans | Furanoid lignans | 1 |
| Shikimates and Phenylpropanoids | Lignans | Furofuranoid lignans | 4 |
| Shikimates and Phenylpropanoids | Lignans | Neolignans | 4 |
| Shikimates and Phenylpropanoids | Phenolic acids (C6-C1) | Gallotannins | 3 |
| Shikimates and Phenylpropanoids | Phenolic acids (C6-C1) | Gallotannins+Simple phenolic acids | 1 |
| Shikimates and Phenylpropanoids | Phenolic acids (C6-C1) | Shikimic acids and derivatives+Simple phenolic acids | 5 |
| Shikimates and Phenylpropanoids | Phenolic acids (C6-C1) | Simple phenolic acids | 23 |
| Shikimates and Phenylpropanoids | Phenolic acids (C6-C1)+Phenylpropanoids (C6-C3) | Cinnamic acids and derivatives+Gallotannins+Simple phenolic acids | 1 |
| Shikimates and Phenylpropanoids | Phenolic acids (C6-C1)+Phenylpropanoids (C6-C3) | Cinnamic acids and derivatives+Shikimic acids and derivatives | 1 |
| Shikimates and Phenylpropanoids | Phenolic acids (C6-C1)+Phenylpropanoids (C6-C3) | Cinnamic acids and derivatives+Simple phenolic acids | 5 |
| Shikimates and Phenylpropanoids | Phenylethanoids (C6-C2) | Phenylethanoids | 6 |
| Shikimates and Phenylpropanoids | Phenylethanoids (C6-C2)+Phenylpropanoids (C6-C3) | Cinnamic acids and derivatives+Phenylethanoids | 2 |
| Shikimates and Phenylpropanoids | Phenylpropanoids (C6-C3) | Cinnamic acids and derivatives | 49 |
| Shikimates and Phenylpropanoids | Phenylpropanoids (C6-C3) | other | 1 |
| Shikimates and Phenylpropanoids | Stilbenoids | Monomeric stilbenes | 6 |
| Shikimates and Phenylpropanoids | Styrylpyrones | Kavalactones and derivatives | 1 |
| Shikimates and Phenylpropanoids | Styrylpyrones+Diazotetronic acids and derivatives | Kavalactones and derivatives+Pulvinones | 1 |
| Shikimates and Phenylpropanoids | Xanthones | Methyl xanthones+Plant xanthones | 1 |
| Shikimates and Phenylpropanoids | other | other | 34 |
| Shikimates and Phenylpropanoids+Terpenoids | other | other | 1 |
| Terpenoids | Apocarotenoids | Apocarotenoids(ε-) | 5 |
| Terpenoids | Apocarotenoids | Apocarotenoids(ε-)+Megastigmanes | 1 |
| Terpenoids | Apocarotenoids | Megastigmanes | 2 |
| Terpenoids | Diterpenoids | Abietane diterpenoids+Podocarpane diterpenoids | 1 |
| Terpenoids | Diterpenoids | Beyerane diterpenoids+Kaurane and Phyllocladane diterpenoids+Norkaurane diterpenoids+Tetracyclic diterpenoids | 1 |
| Terpenoids | Diterpenoids | Briarane diterpenoids | 1 |
| Terpenoids | Diterpenoids | Cembrane diterpenoids | 2 |
| Terpenoids | Diterpenoids | Colensane and Clerodane diterpenoids | 1 |
| Terpenoids | Diterpenoids | Gibberellins | 2 |
| Terpenoids | Diterpenoids | Labdane diterpenoids | 2 |
| Terpenoids | Diterpenoids | Norlabdane diterpenoids | 1 |
| Terpenoids | Diterpenoids | Phytane diterpenoids | 1 |
| Terpenoids | Diterpenoids | Tetracyclic diterpenoids+Tigliane diterpenoids | 1 |
| Terpenoids | Meroterpenoids | Miscellaneous meroterpenoids | 1 |
| Terpenoids | Meroterpenoids | Prenyl quinone meroterpenoids | 4 |
| Terpenoids | Meroterpenoids | Tetraketide meroterpenoids | 1 |
| Terpenoids | Meroterpenoids+Apocarotenoids+Diterpenoids | Apocarotenoids (β-)+Cyclophytane diterpenoids+Prenyl quinone meroterpenoids | 1 |
| Terpenoids | Meroterpenoids+Sesquiterpenoids | Drimane sesquiterpenoids+Spriromeroterpenoids | 1 |
| Terpenoids | Monoterpenoids | Acyclic monoterpenoids | 5 |
| Terpenoids | Monoterpenoids | Camphane monoterpenoids | 2 |
| Terpenoids | Monoterpenoids | Carane monoterpenoids | 1 |
| Terpenoids | Monoterpenoids | Iridoids monoterpenoids | 8 |
| Terpenoids | Monoterpenoids | Iridoids monoterpenoids+Secoiridoid monoterpenoids | 1 |
| Terpenoids | Monoterpenoids | Irregular monoterpenoids | 2 |
| Terpenoids | Monoterpenoids | Menthane monoterpenoids | 5 |
| Terpenoids | Monoterpenoids | Pinane monoterpenoids | 2 |
| Terpenoids | Monoterpenoids | Secoiridoid monoterpenoids | 1 |
| Terpenoids | Monoterpenoids | Thujane monoterpenoids | 1 |
| Terpenoids | Monoterpenoids | other | 2 |
| Terpenoids | Sesquiterpenoids | Aromadendrane sesquiterpenoids | 1 |
| Terpenoids | Sesquiterpenoids | Bergamotane sesquiterpenoids | 1 |
| Terpenoids | Sesquiterpenoids | Bisabolane sesquiterpenoids | 1 |
| Terpenoids | Sesquiterpenoids | Cadinane sesquiterpenoids | 1 |
| Terpenoids | Sesquiterpenoids | Caryophyllane sesquiterpenoids | 1 |
| Terpenoids | Sesquiterpenoids | Cedrane and Isocedrane sesquiterpenoids | 1 |
| Terpenoids | Sesquiterpenoids | Cyclonerane sesquiterpenoids | 1 |
| Terpenoids | Sesquiterpenoids | Daucane sesquiterpenoids | 2 |
| Terpenoids | Sesquiterpenoids | Drimane sesquiterpenoids | 3 |
| Terpenoids | Sesquiterpenoids | Elemane sesquiterpenoids | 2 |
| Terpenoids | Sesquiterpenoids | Eremophilane sesquiterpenoids | 3 |
| Terpenoids | Sesquiterpenoids | Eudesmane sesquiterpenoids | 5 |
| Terpenoids | Sesquiterpenoids | Germacrane sesquiterpenoids | 7 |
| Terpenoids | Sesquiterpenoids | Guaiane sesquiterpenoids | 4 |
| Terpenoids | Sesquiterpenoids | Lactarane sesquiterpenoids | 1 |
| Terpenoids | Sesquiterpenoids | Nardosinane sesquiterpenoids | 1 |
| Terpenoids | Sesquiterpenoids | Patchoulane sesquiterpenoids | 1 |
| Terpenoids | Sesquiterpenoids | Pentalenane sesquiterpenoids | 2 |
| Terpenoids | Sesquiterpenoids | Santalane sesquiterpenoids | 1 |
| Terpenoids | Sesquiterpenoids | Thujopsane sesquiterpenoids | 1 |
| Terpenoids | Sesquiterpenoids | other | 2 |
| Terpenoids | Sesquiterpenoids+Monoterpenoids | Acyclic monoterpenoids+Farnesane sesquiterpenoids | 1 |
| Terpenoids | Steroids | Androstane steroids | 6 |
| Terpenoids | Steroids | Androstane steroids+Pregnane steroids | 1 |
| Terpenoids | Steroids | Bufadienolides | 1 |
| Terpenoids | Steroids | Cardenolides | 1 |
| Terpenoids | Steroids | Cholane steroids | 3 |
| Terpenoids | Steroids | Cholane steroids+Cholestane steroids | 1 |
| Terpenoids | Steroids | Cholestane steroids | 3 |
| Terpenoids | Steroids | Cholestane steroids+Ergostane steroids | 1 |
| Terpenoids | Steroids | Estrane steroids | 2 |
| Terpenoids | Steroids | Pregnane steroids | 7 |
| Terpenoids | Steroids | Spirostane steroids | 2 |
| Terpenoids | Steroids | Vitamin D3 and derivatives | 1 |
| Terpenoids | Triterpenoids | Abeolupane triterpenoids+Lupane triterpenoids | 1 |
| Terpenoids | Triterpenoids | Cucurbitane triterpenoids | 1 |
| Terpenoids | Triterpenoids | Cycloartane triterpenoids | 3 |
| Terpenoids | Triterpenoids | Dammarane and Protostane triterpenoids | 34 |
| Terpenoids | Triterpenoids | Dammarane and Protostane triterpenoids+Fusidane triterpenoids | 1 |
| Terpenoids | Triterpenoids | Dammarane and Protostane triterpenoids+Lanostane, Tirucallane and Euphane triterpenoids | 1 |
| Terpenoids | Triterpenoids | Hopane and Moretane triterpenoids | 3 |
| Terpenoids | Triterpenoids | Lanostane, Tirucallane and Euphane triterpenoids | 4 |
| Terpenoids | Triterpenoids | Limonoids | 2 |
| Terpenoids | Triterpenoids | Lupane triterpenoids | 1 |
| Terpenoids | Triterpenoids | Oleanane triterpenoids | 22 |
| Terpenoids | Triterpenoids | Oleanane triterpenoids+Ursane and Taraxastane triterpenoids | 1 |
| Terpenoids | Triterpenoids | Serratane triterpenoids+Ursane and Taraxastane triterpenoids | 1 |
| Terpenoids | Triterpenoids | Ursane and Taraxastane triterpenoids | 6 |
| Terpenoids | other | other | 10 |

# Supplementary Figure 2. The NPClassifier classification results for the compounds detected in the serum.


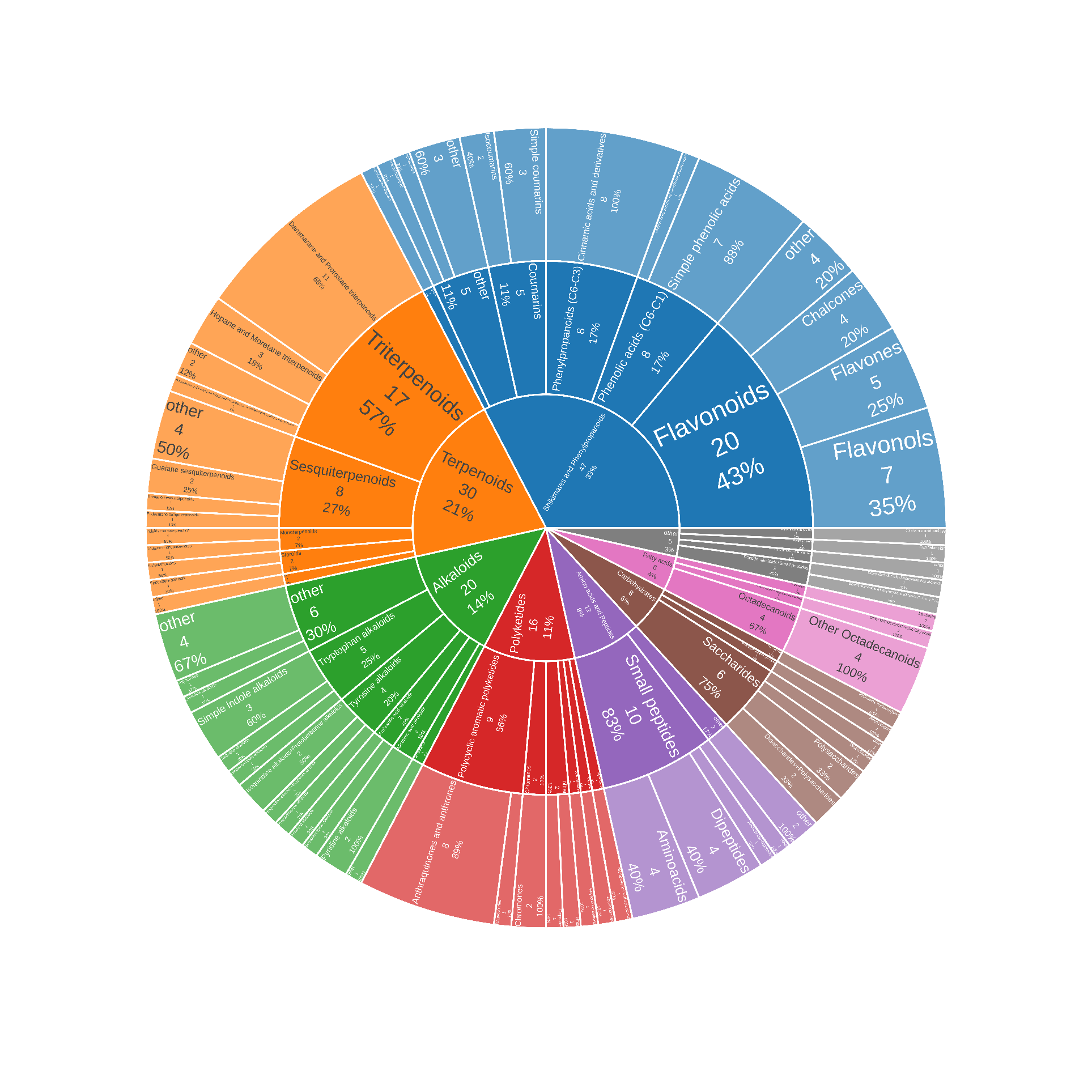


# Supplementary Table 2. The NPClassifier classification results for the compounds detected in the serum.

| Pathway | SuperClass | Class | count |
| --- | --- | --- | --- |
| Alkaloids | Anthranilic acid alkaloids | Acridone alkaloids | 1 |
| Alkaloids | Anthranilic acid alkaloids | Benzodiazepine alkaloids | 1 |
| Alkaloids | Nicotinic acid alkaloids | Pyridine alkaloids | 2 |
| Alkaloids | Pseudoalkaloids | other | 1 |
| Alkaloids | Tryptophan alkaloids | Carboline alkaloids | 1 |
| Alkaloids | Tryptophan alkaloids | Simple indole alkaloids | 3 |
| Alkaloids | Tryptophan alkaloids | Simple oxindole alkaloids | 1 |
| Alkaloids | Tyrosine alkaloids | Isoquinoline alkaloids+Morphinan alkaloids | 1 |
| Alkaloids | Tyrosine alkaloids | Isoquinoline alkaloids+Protoberberine alkaloids | 2 |
| Alkaloids | Tyrosine alkaloids | Protoberberine alkaloids | 1 |
| Alkaloids | other | Polyamines | 1 |
| Alkaloids | other | Quinoline alkaloids | 1 |
| Alkaloids | other | other | 4 |
| Amino acids and Peptides | Small peptides | Aminoacids | 4 |
| Amino acids and Peptides | Small peptides | Aminoacids+Dipeptides | 1 |
| Amino acids and Peptides | Small peptides | Dipeptides | 4 |
| Amino acids and Peptides | Small peptides | other | 1 |
| Amino acids and Peptides | other | other | 2 |
| Carbohydrates | Aminosugars and aminoglycosides | Aminosugars | 1 |
| Carbohydrates | Nucleosides | Pyrimidine nucleos(t)ides | 1 |
| Carbohydrates | Saccharides | Disaccharides | 1 |
| Carbohydrates | Saccharides | Disaccharides+Polysaccharides | 2 |
| Carbohydrates | Saccharides | Polysaccharides | 2 |
| Carbohydrates | Saccharides | other | 1 |
| Fatty acids | Fatty Acids and Conjugates+Octadecanoids | Other Octadecanoids+Oxo fatty acids | 1 |
| Fatty acids | Fatty esters | Lactones | 1 |
| Fatty acids | Octadecanoids | Other Octadecanoids | 4 |
| Polyketides | Chromanes | Chromones | 2 |
| Polyketides | Cyclic polyketides | 4-pyrone derivatives | 1 |
| Polyketides | Macrolides | Zearalenones | 1 |
| Polyketides | Naphthalenes | Naphthalenes and derivatives | 1 |
| Polyketides | Polycyclic aromatic polyketides | Angucyclines | 1 |
| Polyketides | Polycyclic aromatic polyketides | Anthraquinones and anthrones | 8 |
| Polyketides | other | Depsides | 1 |
| Polyketides | other | other | 1 |
| Shikimates and Phenylpropanoids | Coumarins | Isocoumarins | 2 |
| Shikimates and Phenylpropanoids | Coumarins | Simple coumarins | 3 |
| Shikimates and Phenylpropanoids | Flavonoids | Chalcones | 4 |
| Shikimates and Phenylpropanoids | Flavonoids | Flavones | 5 |
| Shikimates and Phenylpropanoids | Flavonoids | Flavonols | 7 |
| Shikimates and Phenylpropanoids | Flavonoids | other | 4 |
| Shikimates and Phenylpropanoids | Lignans | Furofuranoid lignans | 1 |
| Shikimates and Phenylpropanoids | Phenolic acids (C6-C1) | Shikimic acids and derivatives+Simple phenolic acids | 1 |
| Shikimates and Phenylpropanoids | Phenolic acids (C6-C1) | Simple phenolic acids | 7 |
| Shikimates and Phenylpropanoids | Phenylpropanoids (C6-C3) | Cinnamic acids and derivatives | 8 |
| Shikimates and Phenylpropanoids | other | Isoflavones | 1 |
| Shikimates and Phenylpropanoids | other | Phenylethanoids | 1 |
| Shikimates and Phenylpropanoids | other | other | 3 |
| Terpenoids | Monoterpenoids | Iridoids monoterpenoids | 1 |
| Terpenoids | Monoterpenoids | Thujane monoterpenoids | 1 |
| Terpenoids | Sesquiterpenoids | Drimane sesquiterpenoids | 1 |
| Terpenoids | Sesquiterpenoids | Eudesmane sesquiterpenoids | 1 |
| Terpenoids | Sesquiterpenoids | Guaiane sesquiterpenoids | 2 |
| Terpenoids | Sesquiterpenoids | other | 4 |
| Terpenoids | Steroids | Bufadienolides | 1 |
| Terpenoids | Steroids | Spirostane steroids | 1 |
| Terpenoids | Triterpenoids | Dammarane and Protostane triterpenoids | 11 |
| Terpenoids | Triterpenoids | Dammarane and Protostane triterpenoids+Lanostane, Tirucallane and Euphane triterpenoids | 1 |
| Terpenoids | Triterpenoids | Hopane and Moretane triterpenoids | 3 |
| Terpenoids | Triterpenoids | other | 2 |
| Terpenoids | other | other | 1 |
| other | Lysine alkaloids+Small peptides | other | 1 |
| other | Meroterpenoids | Cannabinoids | 1 |
| other | Peptide alkaloids+Small peptides | Dipeptides+Indole diketopiperazine alkaloids (L-Trp, L-Pro) | 1 |
| other | Peptide alkaloids+Small peptides | Dipeptides+Simple diketopiperazine alkaloids | 1 |
| other | Phenylpropanoids (C6-C3) | Cinnamic acid amides | 1 |

# upplementary Figure 3. The BPC results in cation analysis for the following groups: the aqueous extract group of YHP, the blank serum group combined with the aqueous extract group of YHP, the blank serum group, and the YHP drug-containing serum group. These groups are presented sequentially from top to bottom in the order listed.


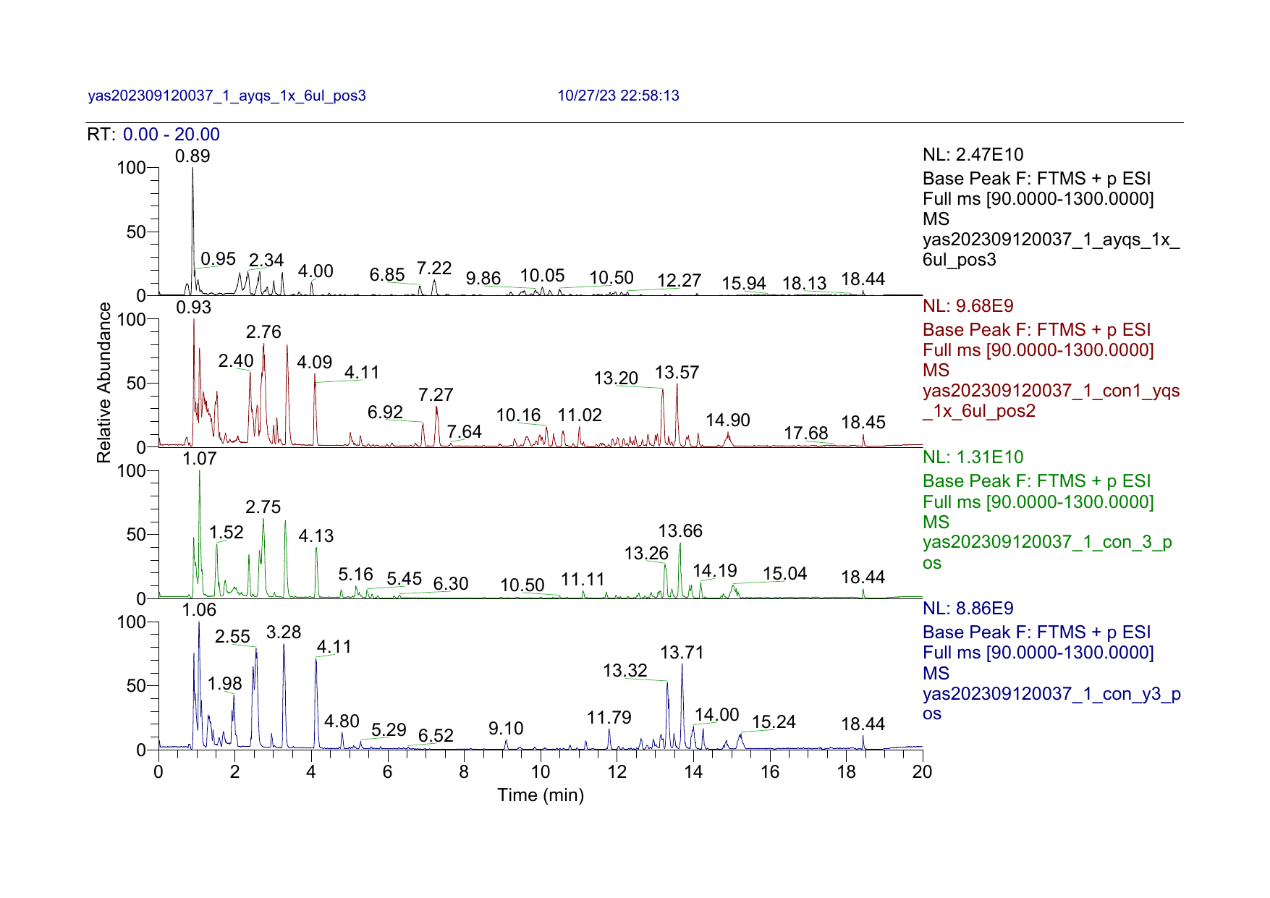


# Supplementary Figure 4. The BPC results in anion analysis for the following groups: the aqueous extract group of YHP, the blank serum group combined with the aqueous extract group of YHP, the blank serum group, and the YHP drug-containing serum group. These groups are presented sequentially from top to bottom in the order listed.


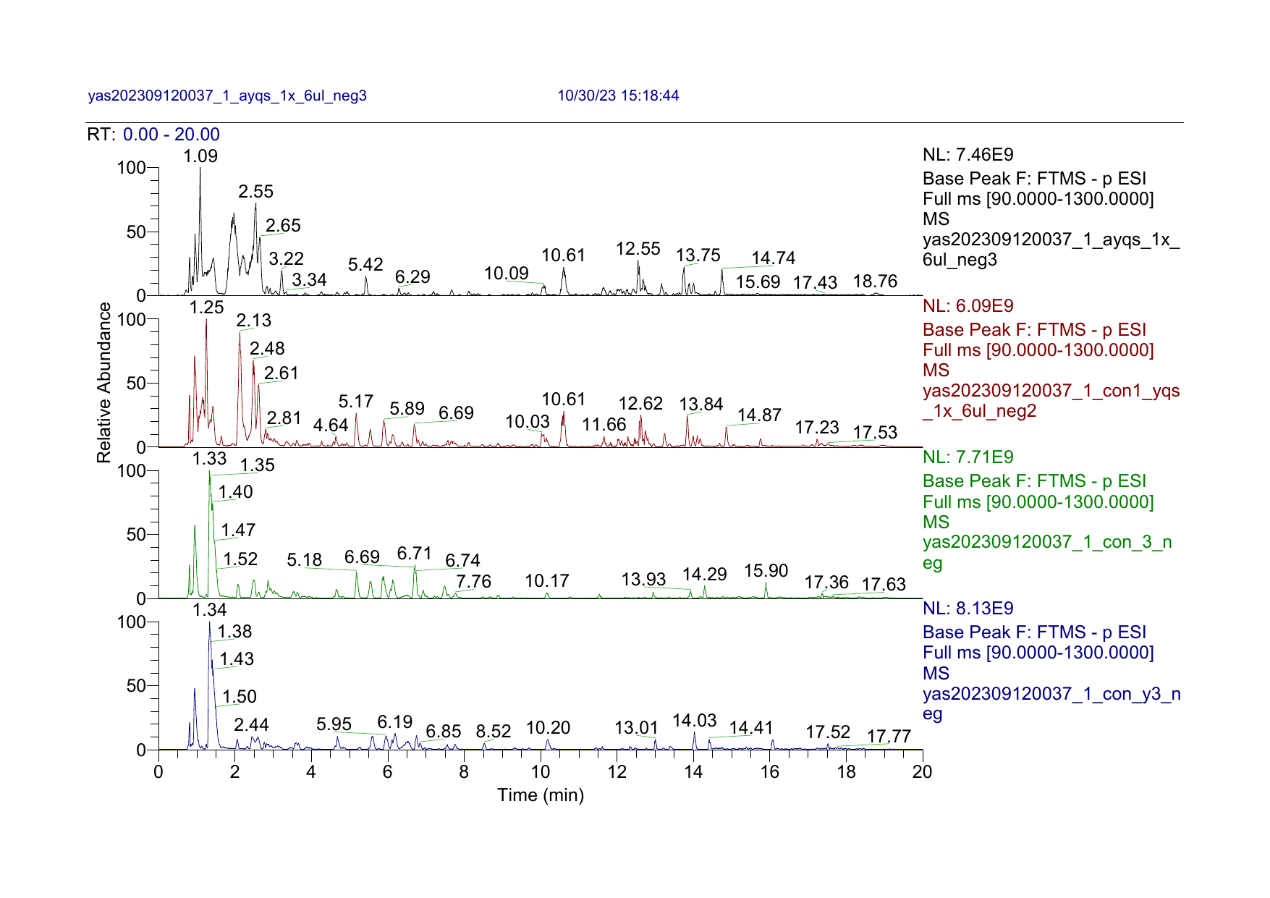


# Supplementary Table 3. Chemical components identified in YHP aqueous extract and YHP-medicated serum using UHPLC-HRMS in cation analysis.

| No. | m/z | RT min | Formula | compound name | adduct | score | Into Blood or Only In Blood or None |
| --- | --- | --- | --- | --- | --- | --- | --- |
| M132T156_2 | 132.1019 | 2.61 | C8H15NO3 | N-Acetyl-D-norleucine | [M+H-C2H2O]+ | 0.9994 | None |
| M188T242_3 | 188.0703 | 4.04 | C11H11NO3 | 3-Indolyllactic acid | [M+H-H2O]+ | 0.982 | None |
| M291T272_2 | 291.0862 | 4.53 | C15H14O6 | Epicatechin | [M+H]+ | 0.9993 | None |
| M403T310 | 403.1386 | 5.16 | C21H22O8 | Flavanone 7-O-.beta.-D-glucoside | [M+H]+ | 0.9528 | None |
| M293T369 | 293.1002 | 6.14 | C23H30O13 | 4-O-Acetyl-.beta.-D-fructofuranosyl 6-O-((2E)-3-phenylprop-2-enoyl)-.alpha.-D-glucopyranoside | [M+H-H2O]+ | 0.9959 | None |
| M801T403 | 801.4977 | 6.72 | C42H72O14 | Ginsenoside A1 | [M+H]+ | 0.9068 | None |
| M405T413_2 | 405.3513 | 6.89 | C42H72O13 | Panaxoside Rg2 | [M+H-C12H28O13]+ | 0.9693 | Into_Blood |
| M823T453 | 823.4809 | 7.55 | C42H72O14 | Ginsenoside A2 | [M+Na]+ | 0.9551 | None |
| M749T577 | 749.4828 | 9.61 | C42H72O13 | Ginsenoside Rg3 | [M+H-2H2O]+ | 0.892 | Into_Blood |
| M407T634_2 | 407.367 | 10.57 | C30H52O3 | Protopanaxadiol | [M+H-3H2O]+ | 0.9733 | Into_Blood |
| M295T761 | 295.2262 | 12.68 | C18H34O5 | 15-Octadecenoic acid, 9,12,13-trihydroxy- | [M+Na]+ | 0.9997 | None |
| M407T799 | 407.3671 | 13.31 | C30H50O2 | Erythrodiol | [M+H-2H2O]+ | 0.9561 | None |
| M789T799 | 789.4755 | 13.32 | C42H70O12 | Ginsenoside Rk1 | [M+Na]+ | 0.9979 | None |
| M284T849_1 | 284.199 | 14.15 | C19H25NO | Levallorphan | [M+H]+ | 0.8711 | Into_Blood |
| M91T436 | 91.0547 | 7.26 | C7H8O | Phenylcarbinol | [M+H-H2O]+ | 0.9996 | None |
| M91T634 | 91.0547 | 10.56 | C7H9N | m-Toluidine | [M+H-NH3]+ | 0.9969 | None |
| M95T101 | 95.0166 | 1.68 | C2H6O2S | Methyl sulfone | [M+H]+ | 0.9945 | None |
| M95T198 | 95.0496 | 3.29 | C6H8O2 | Sorbic acid | [M+H-H2O]+ | 0.9842 | None |
| M95T730 | 95.0859 | 12.16 | C7H12O | exo-Norborneol | [M+H-H2O]+ | 0.9783 | None |
| M95T634 | 95.0859 | 10.57 | C7H12O | 3-Cyclohexene-1-methanol | [M+H-H2O]+ | 0.9783 | None |
| M99T577 | 99.0444 | 9.61 | C5H8O3 | Levulinic acid | [M+H-H2O]+ | 0.9233 | None |
| M100T75 | 100.022 | 1.25 | C5H5NO2S | 2-Methylthiazole-5-carboxylic acid | [M+H-CO2]+ | 0.9278 | None |
| M100T200 | 100.0762 | 3.34 | C6H12N2O3 | 5-Ureidovaleric acid | [M+H-CH3NO2]+ | 1 | Into_Blood |
| M100T184 | 100.1125 | 3.07 | C8H18N2O2S | 2-Amino-N-cyclohexylethanesulfonamide | [M+H-C2H5NO2S]+ | 0.9077 | None |
| M101T185 | 101.0601 | 3.08 | C5H11NO2 | 4-Aminopentanoic acid | [M+H-NH3]+ | 0.9609 | None |
| M101T215 | 101.0601 | 3.58 | C5H8O2 | 3,3-Dimethylacrylic acid | [M+H]+ | 0.9535 | None |
| M102T57 | 102.0553 | 0.95 | C4H9NO3 | O-Methyl-DL-serine | [M+H-H2O]+ | 0.9951 | None |
| M102T275 | 102.0917 | 4.58 | C11H15Cl2NO2 | 2,3-DCPE | [M+H-C6H4Cl2O]+ | 0.9819 | Into_Blood |
| M102T143 | 102.1281 | 2.38 | C12H28N | Triethylhexyammonium cation | [Cat-C6H12]+ | 0.9949 | None |
| M103T1156 | 103.0396 | 19.27 | C4H9NO3 | (S)-4-Amino-2-hydroxybutyric acid | [M+H-NH3]+ | 0.9969 | None |
| M103T198 | 103.0547 | 3.29 | C9H10O3 | (+)-2-Phenyllactic acid | [M+H-CH4O3]+ | 0.987 | None |
| M104T146 | 104.0532 | 2.44 | C13H23NO3S | N-[(4-Methylcyclohexyl)carbonyl]methionine | [M+H-C9H14O3]+ | 0.9771 | None |
| M104T235 | 104.071 | 3.92 | C4H9NO2 | Butanoic acid, 4-amino- | [M+H]+ | 0.8828 | None |
| M104T768 | 104.1073 | 12.8 | [C5H14NO]+ | Choline cation | [M]+ | 0.9802 | None |
| M105T321 | 105.0338 | 5.35 | C22H21NO | N-(3,3-Diphenylpropyl)benzamide | [M+H-C15H17N]+ | 0.9527 | None |
| M105T732 | 105.0338 | 12.21 | C13H13N3OS | Ketone, 2-(allylamino)-4-amino-5-thiazolyl phenyl | [M+H-C6H9N3S]+ | 0.9486 | None |
| M105T797 | 105.0338 | 13.29 | C17H15N3OS | N-(5-Phenethyl-[1,3,4]thiadiazol-2-yl)benzamide | [M+H-C10H11N3S]+ | 0.9268 | None |
| M105T228 | 105.0702 | 3.8 | C17H25NO | 4'-Methyl-.alpha.-pyrrolidinohexanophenone | [M+H-C9H17NO]+ | 0.9806 | None |
| M105T578 | 105.0702 | 9.63 | C8H10O | 4-Methylbenzenemethanol | [M+H-H2O]+ | 0.9969 | None |
| M105T799 | 105.0704 | 13.31 | C15H22N2O | 1-Cyclohexyl-3-phenethylurea | [M+H-C7H14N2O]+ | 0.9958 | None |
| M105T720 | 105.0703 | 12 | C22H25NO | JWH 251 3-methylphenyl isomer | [M+H-C14H17NO]+ | 0.983 | None |
| M106T56_1 | 106.0502 | 0.93 | C3H7NO3 | Serine | [M+H]+ | 0.9871 | None |
| M107T421 | 107.0494 | 7.02 | C8H11NO | (Benzyloxy)(methyl)amine | [M+H-CH5N]+ | 0.9912 | None |
| M107T393 | 107.0494 | 6.55 | C13H16O7 | Helicin | [M+H-C6H10O6]+ | 0.9898 | None |
| M107T295 | 107.0495 | 4.92 | C12H11NO4 | 5-Methyl-4-(phenoxymethyl)-3-isoxazolecarboxylic acid | [M+H-C5H5NO3]+ | 0.9943 | None |
| M107T198 | 107.0495 | 3.29 | C11H14O | Isovalerophenone | [M+H-C4H8]+ | 0.9991 | None |
| M107T436 | 107.0857 | 7.26 | C8H14O2 | Cyclohexanecarboxylic acid, 3-methyl- | [M+H-2H2O]+ | 0.9811 | None |
| M108T161 | 108.0447 | 2.69 | C7H9NO2 | 2-Methoxycarbonyl-N-methylpyrrole | [M+H-CH4O]+ | 0.9944 | None |
| M108T86 | 108.0447 | 1.44 | C6H7NO2 | 6-(Hydroxymethyl)pyridin-3-ol | [M+H-H2O]+ | 0.9519 | None |
| M108T1103 | 108.0685 | 18.39 | C7H10N2 | 2,4-Diaminotoluene | [M+H-CH3]+ | 0.9968 | None |
| M109T612 | 109.0651 | 10.19 | C7H10O2 | 6-Heptynoic acid | [M+H-H2O]+ | 0.7258 | None |
| M109T212 | 109.0287 | 3.54 | C6H6O3 | 2-Hydroxymethyl-5-furfural | [M+H-H2O]+ | 0.9192 | None |
| M109T760 | 109.1016 | 12.67 | C9H17NO3 | 8-Amino-7-oxononanoic acid | [M+H-CH5NO3]+ | 0.9755 | None |
| M109T712 | 109.1016 | 11.87 | C9H14O2 | 4-Oxo-2-nonenal | [M+H-CH2O2]+ | 0.9946 | None |
| M110T336 | 110.0603 | 5.6 | C6H13NO4 | Moranolin | [M+H-3H2O]+ | 0.8737 | None |
| M110T56 | 110.0716 | 0.93 | C11H17N3O4 | N-.alpha.-(tert-Butoxycarbonyl)-L-histidine | [M+H-C6H10O4]+ | 0.9801 | None |
| M111T65 | 111.0444 | 1.08 | C6H12O5 | L-Rhamnose | [M+H-3H2O]+ | 0.9782 | None |
| M112T125 | 112.0505 | 2.09 | C4H5N3O | Cytosine | [M+H]+ | 0.9998 | Into_Blood |
| M112T196 | 112.0507 | 3.27 | C10H14FN3O4 | 2'-Deoxy-2'-fluoro-2'-C-methylcytidine | [M+H-C6H9FO3]+ | 0.9929 | None |
| M112T65 | 112.076 | 1.08 | C6H11NO2 | Nipecotic acid | [M+H-H2O]+ | 0.9659 | None |
| M113T1156_2 | 113.0348 | 19.27 | C9H11FN2O4 | 3'-Fluoro-2',3'-dideoxyuridin | [M+H-C5H7FO2]+ | 0.9817 | None |
| M113T264 | 113.06 | 4.4 | C6H8O2 | 1,4-Cyclohexanedione | [M+H]+ | 0.8424 | None |
| M114T177_1 | 113.9689 | 2.95 | C4H7Cl2NO2 | N-(2,2-Dichloro-1-hydroxyethyl)acetamide | [M+H-C2H4O2+2i]+ | 0.8707 | None |
| M115T318 | 115.0752 | 5.3 | C6H10O2 | (Z)-2-Ethylbut-2-enoic acid | [M+H]+ | 0.8357 | None |
| M116T279 | 116.0708 | 4.65 | C5H9NO2 | L-Proline | [M+H]+ | 0.9988 | None |
| M116T572 | 116.0719 | 9.53 | C5H12N2O2 | Ornithine | [M+H-NH3]+ | 0.9994 | None |
| M117T1178 | 117.0546 | 19.64 | C5H12O5 | L-Lyxitol | [M+H-2H2O]+ | 0.8263 | None |
| M117T847 | 117.0701 | 14.12 | C19H20ClNO | Ecopipam | [M+H-C10H12ClNO]+ | 0.9826 | None |
| M117T223 | 117.0914 | 3.72 | C6H12O2 | Methyl isovalerate | [M+H]+ | 0.7471 | None |
| M118T847 | 118.0653 | 14.12 | C8H7N | 4-Ethynylaniline | [M+H]+ | 0.9576 | None |
| M118T198 | 118.0654 | 3.29 | C8H7N | Indole | [M+H]+ | 0.9775 | None |
| M118T84_2 | 118.0864 | 1.4 | C5H11NO2 | Valine | [M+H]+ | 0.9998 | None |
| M119T300 | 119.0493 | 5 | C10H10O3 | Methyl 4-coumarate | [M+H-C2H4O2]+ | 0.9874 | Into_Blood |
| M119T545 | 119.0494 | 9.08 | C9H10OS | Phenacyl methyl sulfide | [M+H-CH4S]+ | 0.9685 | None |
| M119T534 | 119.0856 | 8.91 | C10H15N | (-)-Deoxyephedrine | [M+H-CH5N]+ | 0.9995 | None |
| M119T476 | 119.0857 | 7.93 | C20H28O | Retinal | [M+H-C11H18O]+ | 0.9437 | None |
| M119T608_2 | 119.0857 | 10.13 | C16H16F3NO2S | 2,4,6-Trimethyl-N-[3-(trifluoromethyl)phenyl]benzenesulfonamide | [M+H-C7H6F3NO2S]+ | 0.9969 | None |
| M119T497 | 119.0857 | 8.29 | C11H17NO2S | N-Mesitylethanamine | [M+H-C2H7NO2S]+ | 0.9943 | None |
| M119T578_2 | 119.0857 | 9.64 | C13H17N | L-Deprenyl | [M+H-C4H7N]+ | 0.9992 | None |
| M119T568 | 119.0857 | 9.47 | C9H13N | .beta.-Methylphenethylamine | [M+H-NH3]+ | 0.9993 | None |
| M120T375 | 120.0446 | 6.25 | C10H12N2O3 | N-(4-Aminobenzoyl)-.beta.-alanine | [M+H-C3H7NO2]+ | 0.9837 | None |
| M120T460 | 120.0444 | 7.67 | C7H7NO2 | 3-Pyridylacetic acid | [M+H-H2O]+ | 0.9893 | None |
| M120T69 | 120.0657 | 1.16 | C4H9NO3 | Threonine | [M+H]+ | 0.992 | None |
| M120T197_2 | 120.0809 | 3.29 | C14H20N2O3S | Phe-Met | [M+H-C2H4O2]+ | 0.9997 | None |
| M120T364 | 120.0809 | 6.07 | C16H14N2OS | Phenylalanyl phenylthiohydantoin | [M+H-C8H5NOS]+ | 0.9982 | None |
| M120T739 | 120.0811 | 12.32 | C20H26N2 | o-Toluidine, 4,4'-cyclohexylidenedi- | [M+H-C12H17N]+ | 0.9822 | None |
| M121T238 | 121.0286 | 3.97 | C13H11NO3 | 4,4'-Dihydroxybenzanilide | [M+H-C6H7NO]+ | 0.9873 | None |
| M121T854 | 121.065 | 14.23 | C15H16FNO | (4-Fluorobenzyl)-p-anisylamine | [M+H-C7H8FN]+ | 0.9975 | None |
| M121T719 | 121.065 | 11.98 | C16H18FNO | 2-(4-Fluorophenyl)ethyl-m-anisylamine | [M+H-C8H10FN]+ | 0.9976 | None |
| M121T156 | 121.065 | 2.61 | C8H11NO | 1-Amino-2-ethoxybenzene | [M+H-NH3]+ | 0.9917 | None |
| M121T874 | 121.065 | 14.57 | C8H8N2O4 | 4,5-Dinitro-o-xylene | [M+H-N2O3]+ | 0.9803 | None |
| M121T506 | 121.065 | 8.43 | C8H11NO | (R)-(-)-2-Phenylglycinol | [M+H-NH3]+ | 0.8976 | None |
| M121T619 | 121.1012 | 10.32 | C15H26O | Cedrol | [M+H-C6H14O]+ | 0.9802 | None |
| M121T436 | 121.1012 | 7.26 | C9H16O2 | .gamma.-Nonalactone | [M+H-2H2O]+ | 0.8556 | None |
| M121T578 | 121.1013 | 9.64 | C9H18O3 | 9-Hydroxynonanoic acid | [M+H-3H2O]+ | 0.848 | None |
| M122T294 | 122.0602 | 4.9 | C16H21N3O8 | 4-(Methylnitrosamino)-1-(3-pyridyl)-1-butanone N-.beta.-D-Glucuronide | [M+H-C9H14N2O7]+ | 0.9977 | None |
| M122T309 | 122.0966 | 5.15 | C15H16N2O | N-(4-Ethylphenyl)-N'-phenylurea | [M+H-C7H5NO]+ | 0.9747 | None |
| M122T227 | 122.0966 | 3.78 | C15H15ClN2O | N-(3-Chlorophenyl)-N'-(2-phenylethyl)urea | [M+H-C7H4ClNO]+ | 0.8785 | None |
| M123T272 | 123.0437 | 4.53 | C21H34O2 | Bilobol | [M+H-C14H28]+ | 0.9775 | None |
| M123T330 | 123.0442 | 5.51 | C12H16O2 | 4-Caproylphenol | [M+H-C5H10]+ | 0.9723 | None |
| M123T131 | 123.0554 | 2.19 | C6H6N2O | Niacinamide | [M+H]+ | 0.9986 | None |
| M123T511 | 123.117 | 8.51 | C19H30O8 | 3-Hydroxy-3,5,5-trimethyl-4-(3-oxobut-1-en-1-ylidene)cyclohexyl .beta.-D-glucopyranoside | [M+H-C10H16O8]+ | 0.9764 | None |
| M123T562 | 123.117 | 9.37 | C19H24O3 | Prallethrin | [M+H-C10H10O3]+ | 0.9965 | None |
| M123T634 | 123.1171 | 10.57 | C15H24O | (+)-Cycloisolongifol-5-ol | [M+H-C6H10O]+ | 0.9923 | None |
| M123T619 | 123.1171 | 10.32 | C15H26O | Patchouli alcohol | [M+H-C6H12O]+ | 0.9925 | None |
| M124T279 | 124.0758 | 4.66 | C7H9NO | 2-Amino-m-cresol | [M+H]+ | 0.8492 | None |
| M124T1070 | 124.0866 | 17.83 | C7H11N3O2 | 1-Methyl-L-histidine | [M+H-CH2O2]+ | 0.9995 | None |
| M125T452 | 125.0598 | 7.53 | C8H8O3 | cis-1,2,3,6-Tetrahydrophthalic anhydride | [M+H-CO]+ | 0.924 | None |
| M126T166 | 126.0664 | 2.76 | C10H15N3O4 | 5-Methyldeoxycytidine | [M+H-C5H8O3]+ | 0.9998 | None |
| M128T246 | 128.0431 | 4.1 | C5H5N3O4 | 5-Aminoorotic acid | [M+H-CO2]+ | 0.9617 | None |
| M128T323 | 128.1071 | 5.38 | C10H21NOS | Tillam | [M+H-C3H8S]+ | 0.9912 | None |
| M129T46 | 129.1387 | 0.77 | C7H16N2 | 3-Dimethylaminopiperidine | [M+H]+ | 0.9266 | None |
| M131T623 | 131.0489 | 10.38 | C19H20O2 | Yashabushiketol | [M+H-C10H14O]+ | 0.9969 | None |
| M131T369 | 131.0491 | 6.15 | C11H11NO3 | Cinnamoylglycine | [M+H-C2H5NO2]+ | 0.9995 | None |
| M131T855 | 131.0492 | 14.25 | C10H7IO2 | 3-Iodo-4-(2-propynyloxy)benzaldehyde | [M+H-CHIO]+ | 0.9118 | None |
| M131T436 | 131.0855 | 7.26 | C10H16O3 | cis-Pinonic acid | [M+H-3H2O]+ | 0.9936 | None |
| M131T847 | 131.0856 | 14.12 | C12H15NO2 | 2-Hydroxy-N-tetralin-1-yl-acetamide | [M+H-C2H5NO2]+ | 0.9981 | None |
| M131T54_2 | 131.1292 | 0.9 | C5H14N4 | Agmatine | [M+H]+ | 0.9903 | None |
| M132T128 | 132.0768 | 2.14 | C4H9N3O2 | Creatine | [M+H]+ | 0.9966 | None |
| M132T242 | 132.0808 | 4.04 | C10H14N2 | N-(2,4-Dimethylphenyl)-N'-methylformamidine | [M+H-CH5N]+ | 0.9973 | None |
| M132T217 | 132.1015 | 3.62 | C6H13NO2 | Isoleucine | [M+H]+ | 0.9967 | None |
| M133T98 | 133.0319 | 1.63 | C11H13N3O6S | N-(2,4-Dinitrophenyl)-L-methionine | [M+H-C6H5N3O4]+ | 0.9911 | None |
| M133T56 | 133.0608 | 0.93 | C4H8N2O3 | Asparagine | [M+H]+ | 0.9605 | None |
| M133T730 | 133.1012 | 12.16 | C10H16O2 | (S)-8-Hydroxy-p-menth-1-en-6-one | [M+H-2H2O]+ | 0.9866 | None |
| M133T436 | 133.1012 | 7.26 | C13H20O | 4-(2,4,6-Trimethyl-3-cyclohexen-1-yl)-3-buten-2-one | [M+H-C3H8O]+ | 0.9985 | None |
| M133T670 | 133.1013 | 11.17 | C10H14O | (-)-Myrtenal | [M+H-H2O]+ | 0.9965 | None |
| M134T67 | 134.0271 | 1.11 | C4H7NO2S | Thioproline | [M+H]+ | 0.9945 | None |
| M134T311 | 134.0601 | 5.18 | C8H7NO | Oxindol | [M+H]+ | 0.9998 | None |
| M134T355 | 134.0604 | 5.91 | C9H7F3N2O3 | 2,2,2-Trifluoro-N-(2-methyl-5-nitrophenyl)acetamide | [M+H-CF3NO2]+ | 0.9984 | None |
| M135T47 | 135.0026 | 0.78 | C6H2N2S | 3,4-Dicyanothiophene | [M+H]+ | 0.9648 | None |
| M135T392 | 135.0441 | 6.54 | C8H6O2 | Phthalide | [M+H]+ | 0.9968 | None |
| M135T755 | 135.08 | 12.58 | C9H10O | 3,4-Dimethylbenzaldehyde | [M+H]+ | 0.9632 | None |
| M135T619 | 135.1168 | 10.32 | C15H26O | Farnesol | [M+H-C5H12O]+ | 0.9031 | None |
| M135T436 | 135.1168 | 7.26 | C12H18O2 | Sabinyl acetate | [M+H-C2H4O2]+ | 0.9729 | Into_Blood |
| M135T578 | 135.1168 | 9.64 | C15H26O | (-)-Globulol | [M+H-C5H12O]+ | 0.9671 | None |
| M136T212 | 136.0757 | 3.54 | C8H9NO | Phenacylamine | [M+H]+ | 0.9872 | None |
| M136T252 | 136.0757 | 4.2 | C15H22N2O4 | Tyr-Leu | [M+H-C7H13NO3]+ | 0.9946 | None |
| M136T382 | 136.0758 | 6.37 | C16H13NO4 | 2-(Phenacylcarbamoyl)benzoic acid | [M+H-C8H4O3]+ | 0.9837 | None |
| M137T162 | 137.0456 | 2.71 | C5H4N4O | Hypoxanthine | [M+H]+ | 0.9985 | None |
| M137T354 | 137.0597 | 5.91 | C28H41NO3 | Arvanil | [M+H-C20H33NO]+ | 0.993 | None |
| M137T202 | 137.0597 | 3.37 | C17H26O3 | [6]-Paradol | [M+H-C9H18O]+ | 0.9973 | None |
| M138T62 | 138.0541 | 1.04 | C7H7NO2.HCl | Trigonelline hcl | [M+H]+ | 0.9991 | None |
| M138T375 | 138.0549 | 6.25 | C11H15NO2 | 4-(Butylamino)benzoic acid | [M+H-C4H8]+ | 0.9934 | None |
| M138T279 | 138.0549 | 4.65 | C7H7NO2 | 4-Pyridineacetic acid | [M+H]+ | 0.9936 | None |
| M138T301 | 138.0548 | 5.02 | C7H7NO2 | 3-Acetoxypyridine | [M+H]+ | 0.9824 | None |
| M138T233_1 | 138.055 | 3.89 | C10H12N2O3 | Benzoic acid, p-(3-ethylureido)- | [M+H-C3H5NO]+ | 0.9962 | None |
| M139T113_1 | 139.0026 | 1.89 | C6H6O6 | trans-Aconitic acid | [M+H-2H2O]+ | 0.9991 | None |
| M139T296_1 | 139.0386 | 4.93 | C15H14O6 | (-)-Catechin | [M+H-C8H8O3]+ | 0.9923 | None |
| M139T296_2 | 139.0754 | 4.93 | C8H12O3 | Dihydroterrein | [M+H-H2O]+ | 0.8718 | None |
| M139T717_2 | 139.1117 | 11.95 | C14H26O4 | Diethyl sebacate | [M+H-C5H12O3]+ | 0.8177 | None |
| M140T86 | 140.032 | 1.43 | C9H9NO5 | 2-(4-Nitrophenoxy)propionic acid | [M+H-C3H4O2]+ | 0.8804 | None |
| M140T61 | 140.0691 | 1.01 | C5H11NO2 | GABA methyl ester | [M+Na]+ | 0.9642 | None |
| M140T313_2 | 140.0703 | 5.22 | C7H9NO2 | 3-Amino-2,3-dihydrobenzoic acid | [M+H]+ | 0.9726 | None |
| M140T217 | 140.0706 | 3.62 | C7H13NO4 | Spermidic acid | [M+H-2H2O]+ | 0.9626 | None |
| M141T601 | 141.0181 | 10.01 | C6H10O7 | Glucuronic acid | [M+H-3H2O]+ | 0.9892 | None |
| M141T677 | 141.0544 | 11.28 | C7H12O5 | 3-Isopropylmalic acid | [M+H-2H2O]+ | 0.7602 | None |
| M142T142 | 142.0499 | 2.37 | C9H5BrN2 | 6-Bromo-3-cyanoindole | [M+H-Br]+ | 0.9879 | None |
| M142T242 | 142.0651 | 4.04 | C11H12N2 | 3-[(Methylamino)methyl]quinoline | [M+H-CH5N]+ | 0.9858 | None |
| M143T142 | 143.0344 | 2.37 | C6H7ClN2 | 2-Chloro-3-ethylpyrazine | [M+H]+ | 0.9945 | None |
| M143T86 | 143.1179 | 1.44 | C7H14N2O | 3-Acetamidopiperidine | [M+H]+ | 0.9119 | None |
| M144T279 | 144.0808 | 4.66 | C11H11NO2 | N-Methylindoleacetic acid | [M+H-CH2O2]+ | 0.9994 | Into_Blood |
| M144T242_2 | 144.0809 | 4.04 | C11H14N2 | N-Methyltryptamine | [M+H-CH5N]+ | 0.9983 | None |
| M144T66_2 | 144.1019 | 1.1 | C7H13NO2 | Stachydrine | [M+H]+ | 0.9994 | None |
| M144T140_1 | 144.1019 | 2.34 | C7H16N2O2 | L-.beta.-Homolysine | [M+H-NH3]+ | 0.9785 | None |
| M144T97 | 144.1019 | 1.62 | C7H13NO2 | Cyclobutanealanine | [M+H]+ | 0.9112 | None |
| M145T505 | 145.0492 | 8.42 | C6H10O5 | Levoglucosan | [M+H-H2O]+ | 0.7295 | None |
| M145T436 | 145.1011 | 7.26 | C11H14O | 4'-Methylbutyrophenone | [M+H-H2O]+ | 0.7116 | None |
| M145T847 | 145.1012 | 14.12 | C14H16N2 | Atipamezole | [M+H-C3H4N2]+ | 0.9513 | None |
| M146T242 | 146.0599 | 4.04 | C9H7NO | Indole-3-carboxaldehyde | [M+H]+ | 0.9968 | None |
| M146T64 | 146.0929 | 1.06 | C5H11N3O2 | 4-Guanidinobutyric acid | [M+H]+ | 0.9989 | None |
| M146T63 | 146.1175 | 1.06 | C7H16NO2 | N-trimethyl-GABA | [Cat]+ | 0.9865 | None |
| M146T49 | 146.1652 | 0.81 | C7H19N3 | Spermidine | [M+H]+ | 0.9948 | Into_Blood |
| M147T300 | 147.0439 | 5 | C9H8O3 | trans-3-Coumaric acid | [M+H-H2O]+ | 0.9992 | None |
| M147T534 | 147.0798 | 8.9 | C10H12O2 | Thymoquinone | [M+H-H2O]+ | 0.8842 | None |
| M147T907 | 147.0804 | 15.11 | C11H12O3 | (2-Allylphenoxy)acetic acid | [M+H-CH2O2]+ | 0.9782 | Into_Blood |
| M147T497 | 147.116 | 8.29 | C13H22O4 | 2-Pentenedioic acid, 2-octyl- | [M+H-C2H8O4]+ | 0.7906 | None |
| M147T436 | 147.1172 | 7.26 | C11H16O | cis-Jasmone | [M+H-H2O]+ | 0.9926 | None |
| M148T403 | 148.0756 | 6.71 | C19H18N2O4S | Ketopioglitazone (M-III) | [M+H-C10H9NO3S]+ | 0.9878 | None |
| M148T86_1 | 148.0969 | 1.43 | C6H13NO3 | 2,2-Diethoxyacetamide | [M+H]+ | 0.9001 | None |
| M149T872 | 149.0232 | 14.53 | C8H8O5 | 3,4-Dihydroxymandelic acid | [M+H-2H2O]+ | 0.9965 | None |
| M149T360 | 149.0597 | 6 | C9H8O2 | trans-Cinnamic acid | [M+H]+ | 0.9952 | None |
| M149T541 | 149.0598 | 9.02 | C9H11NO2 | 3-Phenyl-.beta.-alanine | [M+H-NH3]+ | 0.9971 | Into_Blood |
| M149T495 | 149.0598 | 8.25 | C12H19NO2S | 2,5-Dimethoxy-4-ethylthiophenethylamine | [M+H-C3H11NS]+ | 0.8293 | None |
| M149T919 | 149.0961 | 15.31 | C10H12O | Cuminaldehyde | [M+H]+ | 0.8744 | None |
| M149T292_2 | 149.1174 | 4.86 | C7H16O3 | 1-(2-Methoxyethoxy)-2-methyl-2-propanol | [M+H]+ | 0.861 | None |
| M149T667 | 149.1325 | 11.12 | C16H30O2 | 2-Carene-4-methanol | [M+H-H2O]+ | 0.9171 | None |
| M150T98 | 150.0579 | 1.63 | C9H15NO5S | Methionine, N-(3-carboxy-1-oxopropyl)- | [M+H-C4H4O3]+ | 0.996 | None |
| M150T326 | 150.0901 | 5.43 | C9H13NO2 | 5-Acetyl-2,6-dimethyl-2,3-dihydropyridin-4(1H)-one | [M+H-H2O]+ | 0.9682 | None |
| M150T315 | 150.1277 | 5.25 | C10H15N | 1-Methyl-3-phenylpropylamine | [M+H]+ | 0.989 | None |
| M151T638 | 151.0753 | 10.63 | C15H20O4 | .gamma.-CEHC | [M+H-C6H10O2]+ | 0.9864 | None |
| M151T672 | 151.1117 | 11.21 | C10H14O | (+)-Carvone | [M+H]+ | 0.8062 | None |
| M152T412 | 152.0705 | 6.87 | C8H9NO2 | Methyl 3-aminobenzoate | [M+H]+ | 0.995 | None |
| M153T187_1 | 153.0182 | 3.11 | C20H20O14 | Hamamelitannin | [M+H-C13H16O10]+ | 0.9787 | None |
| M153T358 | 153.0545 | 5.96 | C8H8O3 | Vanillin | [M+H]+ | 0.9857 | None |
| M153T684 | 153.1273 | 11.4 | C10H20O3 | 4-Hydroxycapric acid | [M+H-2H2O]+ | 0.943 | None |
| M153T717 | 153.1274 | 11.95 | C12H20O3 | (-)-Dihydrojasmonic acid | [M+H-C2H4O2]+ | 0.9189 | None |
| M154T163 | 154.0499 | 2.71 | C8H9NO3 | Methyl 5-cyclopropylisoxazole-3-carboxylate | [M+H-CH2]+ | 0.9621 | None |
| M154T382 | 154.0866 | 6.37 | C15H17NO4 | Benzoylnorecgonine | [M+H-C7H6O2]+ | 0.901 | None |
| M154T65 | 154.0975 | 1.09 | C17H25N3O2 | Vildagliptin | [M+H-C10H14O]+ | 0.9943 | None |
| M155T87_2 | 155.0436 | 1.45 | C4H8N2O3 | 3-Ureidopropionic acid | [M+Na]+ | 0.9001 | None |
| M155T170 | 155.0815 | 2.84 | C7H10N2O2 | Pyrrolopiperazine-2,5-dione | [M+H]+ | 0.9624 | Into_Blood |
| M155T418 | 155.1064 | 6.97 | C9H16O3 | (2E)-4-Hydroxynon-2-enoic acid | [M+H-H2O]+ | 0.8864 | None |
| M155T661 | 155.1072 | 11.02 | C9H16O3 | 8-Oxononanoic acid | [M+H-H2O]+ | 0.9787 | None |
| M156T56 | 156.0768 | 0.93 | C6H9N3O2 | Histidine | [M+H]+ | 0.9989 | None |
| M156T207 | 156.1019 | 3.44 | C17H21NO4 | Scopolamine | [M+H-C9H8O2]+ | 0.9378 | None |
| M156T271 | 156.102 | 4.51 | C9H15NO3 | PRIMA-1 | [M+H-CH2O]+ | 0.8988 | None |
| M157T113 | 157.0132 | 1.89 | C6H6O6 | cis-Aconitic acid | [M+H-H2O]+ | 0.9848 | None |
| M157T659 | 157.1223 | 10.98 | C9H16O2 | 4-Hydroxynonenal | [M+H]+ | 0.9682 | None |
| M158T350_2 | 158.0459 | 5.83 | C6H9NO5 | N-Acetyl-L-aspartic acid | [M+H-H2O]+ | 0.9875 | None |
| M158T219 | 158.0813 | 3.65 | C7H11NO3 | N-Acetyl-D-proline | [M+H]+ | 0.9962 | None |
| M158T267 | 158.0816 | 4.46 | C9H17NO4 | Diethyl L-glutamate | [M+H-C2H6O]+ | 0.9972 | None |
| M158T290 | 158.0953 | 4.84 | C13H14N2O2 | Tetrahydroharman-3-carboxylic acid | [M+H-C2H3NO2]+ | 0.9918 | None |
| M158T88_2 | 158.1175 | 1.46 | C8H15NO2 | 1-(Aminomethyl)cyclopentaneacetic acid | [M+H]+ | 0.9994 | None |
| M158T680 | 158.154 | 11.33 | C24H40O5 | Hyocholic acid | [M+H]+ | 0.9876 | None |
| M158T661 | 158.154 | 11.02 | C9H19NO | N-Isobutyl-3-methylbutanamide | [M+H]+ | 0.9221 | None |
| M159T242_2 | 159.0911 | 4.04 | C18H15ClN2O3 | Benzotript | [M+H-C8H5ClO3]+ | 0.9974 | None |
| M159T160 | 159.1128 | 2.67 | C14H28N4O4 | Val-Ala-Lys | [M+2H]2+ | 0.7827 | None |
| M159T471 | 159.116 | 7.85 | C13H18O3 | 2-Hydroxyibuprofen | [M+H-CH4O3]+ | 0.8967 | None |
| M159T579 | 159.1167 | 9.64 | C19H30O2 | Stanolone | [M+H-C7H16O2]+ | 0.9981 | None |
| M160T739 | 160.0757 | 12.32 | C10H9NO | 1-(1H-Indol-5-yl)ethanone | [M+H]+ | 0.9399 | None |
| M160T283_1 | 160.0758 | 4.71 | C10H11NO2 | 5-Hydroxytryptophol | [M+H-H2O]+ | 0.9992 | None |
| M160T197 | 160.0764 | 3.28 | C12H16N2O | Bufotenine | [M+H-C2H7N]+ | 0.9975 | None |
| M160T262 | 160.0969 | 4.36 | C7H13NO3 | N-Acetylvaline | [M+H]+ | 0.9507 | None |
| M161T849 | 161.0961 | 14.14 | C11H14O2 | 3-tert-Butylbenzoic acid | [M+H-H2O]+ | 0.855 | None |
| M161T824 | 161.0965 | 13.73 | C15H21NO | 3-Methyl-.alpha.-pyrrolidinobutiophenone | [M+H-C4H9N]+ | 0.9148 | None |
| M161T807 | 161.1324 | 13.45 | C15H22O2 | a-Santal-10-en-12-oic acid | [M+H-C3H6O2]+ | 0.9898 | None |
| M162T158 | 161.5973 | 2.63 | C15H23N5O3 | Phe-Arg | [M+2H]2+ | 0.9893 | None |
| M162T795 | 162.0913 | 13.26 | C11H13NO3 | N-Acetyl-D-phenylalanine | [M+H-CH2O2]+ | 0.9673 | None |
| M162T59 | 162.1125 | 0.98 | C7H15NO3 | L-Carnitine | [M+H]+ | 0.9991 | None |
| M163T266 | 163.039 | 4.43 | C15H12O5 | Butein | [M+H-C6H6O2]+ | 0.9849 | Into_Blood |
| M163T79 | 163.0601 | 1.32 | C6H12O6 | Levulose | [M+H-H2O]+ | 0.9731 | None |
| M163T854 | 163.0755 | 14.23 | C17H16O4 | 1,2-Propanediol, 1,2-dibenzoate | [M+H-C7H6O2]+ | 0.9666 | None |
| M163T878 | 163.1117 | 14.63 | C11H16O2 | 4-tert-Butyl-2-(hydroxymethyl)phenol | [M+H-H2O]+ | 0.9863 | None |
| M163T667 | 163.148 | 11.11 | C12H22O2 | .delta.-Dodecalactone | [M+H-2H2O]+ | 0.9533 | None |
| M164T724 | 164.1071 | 12.06 | C10H13NO | N-Methyl-N-benzylacetamide | [M+H]+ | 0.8877 | None |
| M165T349 | 165.0546 | 5.82 | C9H8O3 | p-Coumaric acid | [M+H]+ | 0.9994 | Into_Blood |
| M165T491 | 165.0544 | 8.19 | C14H15ClN2O3 | 3-[(4-Chloro-3,5-dimethyl-1H-pyrazol-1-yl)methyl]-4-methoxybenzoic acid | [M+H-C5H7ClN2]+ | 0.8007 | None |
| M165T657 | 165.0909 | 10.94 | C14H20O2 | Chroman C1 | [M+H-C4H8]+ | 0.9958 | None |
| M165T534 | 165.0909 | 8.9 | C10H12O2 | 2-Phenylbutyric acid | [M+H]+ | 0.968 | None |
| M166T165 | 166.05 | 2.75 | C8H9NO4 | 4-Pyridoxic acid | [M+H-H2O]+ | 0.9895 | None |
| M166T59 | 166.0533 | 0.98 | C5H11NO3S | Methionine sulfoxide | [M+H]+ | 0.9906 | None |
| M166T198_2 | 166.086 | 3.29 | C9H11NO2 | Phenylalanine | [M+H]+ | 0.9998 | None |
| M167T216 | 167.0706 | 3.6 | C19H23ClN2O5S | 4-({4-[(4-Chlorophenyl)sulfonyl]-1-piperazinyl}methyl)-2,6-dimethoxyphenol | [M+H-C10H13ClN2O2S]+ | 0.9781 | None |
| M167T292 | 167.1069 | 4.87 | C10H14O2 | (S)-Perillic acid | [M+H]+ | 0.8416 | None |
| M168T191 | 168.0291 | 3.18 | C7H5NO4 | 2,5-Dicarboxypyridine | [M+H]+ | 0.9799 | None |
| M168T329 | 168.0655 | 5.48 | C16H16N2O4 | Phenmedipham | [M+H-C8H7NO]+ | 0.8889 | None |
| M168T181 | 168.0765 | 3.01 | C7H9N3O2 | 2-Nitro-4,6-diaminotoluene | [M+H]+ | 0.7439 | None |
| M168T177 | 168.1019 | 2.96 | C9H13NO2 | 3-Methoxytyramine | [M+H]+ | 0.9527 | None |
| M169T618 | 169.0494 | 10.3 | C14H20O10 | .beta.-D-Mannopyranose, 1,3,4,6-tetraacetate | [M+H-C6H12O6]+ | 0.9671 | None |
| M169T292 | 169.1223 | 4.87 | C10H14O | Perillaldehyde | [M+H+H2O]+ | 0.7567 | None |
| M170T242 | 170.0602 | 4.04 | C11H11N3O | 2-(1H-Pyrrol-1-yl)benzohydrazide | [M+H-N2H4]+ | 0.9926 | None |
| M170T58 | 170.0925 | 0.96 | C7H11N3O2 | N(pi)-Methyl-L-histidine | [M+H]+ | 0.9221 | None |
| M171T422 | 171.1016 | 7.04 | C9H16O4 | Azelaic acid | [M+H-H2O]+ | 0.8579 | None |
| M171T513 | 171.138 | 8.55 | C10H18O2 | .delta.-Decalactone | [M+H]+ | 0.9746 | None |
| M172T951 | 172.0718 | 15.85 | C11H12N2O | 1H-Indole-1-propanamide | [M+H-NH3]+ | 0.994 | None |
| M173T173 | 173.0785 | 2.89 | C8H12O4 | 1,4-cyclohexanedicarboxylic acid | [M+H]+ | 0.9975 | None |
| M173T436 | 173.1324 | 7.26 | C13H18O | (5S)-1,2,3,4,5,6,7,8,9,10-Decahydro-5,9-methanobenzo[8]annulen-11-one | [M+H-H2O]+ | 0.9881 | None |
| M174T270 | 174.0551 | 4.5 | C10H7NO2 | 3-Isoquinaldic acid | [M+H]+ | 0.9877 | None |
| M174T402 | 174.076 | 6.69 | C11H18N2O8 | 1,3-PDTA | [M+H-C4H7NO4]+ | 0.7844 | None |
| M175T578 | 175.1481 | 9.64 | C13H20O | (E)-.alpha.-Ionone | [M+H-H2O]+ | 0.9893 | None |
| M176T181 | 176.0902 | 3.01 | C6H13N3O3 | L-Citrulline | [M+H]+ | 0.9805 | None |
| M176T447 | 176.0705 | 7.44 | C16H19NO7 | 3-(Carboxymethyl)-1-.beta.-D-glucopyranosyl-1H-indole | [M+H-C6H10O5]+ | 0.9998 | None |
| M177T284 | 177.0529 | 4.74 | C16H14O6 | (-)-Homoeriodictyol | [M+H-H2O]+ | 0.9788 | None |
| M177T373 | 177.0531 | 6.22 | C10H10O4 | Ferulic acid | [M+H-H2O]+ | 0.9694 | None |
| M177T177 | 177.1023 | 2.96 | C10H12N2O | Serotonin | [M+H]+ | 0.9998 | None |
| M177T436_2 | 177.1637 | 7.26 | C13H22O | 7,8-Dihydro-.alpha.-ionone | [M+H-H2O]+ | 0.9592 | None |
| M178T834 | 178.0417 | 13.91 | C10H8ClN | 2-Chloro-6-methylquinoline | [M+H]+ | 0.9779 | None |
| M178T242 | 178.0533 | 4.03 | C6H11NO3S | N-Formylmethionine | [M+H]+ | 0.9533 | Into_Blood |
| M178T207 | 178.0879 | 3.45 | C10H11NO2 | 3-Carboxy-1,2,3,4-tetrahydroisoquinoline | [M+H]+ | 0.9669 | None |
| M179T412 | 179.0338 | 6.87 | C9H6O4 | 5,7-Dihydroxychromone | [M+H]+ | 0.9894 | None |
| M179T340 | 179.0702 | 5.66 | C10H10O3 | Mellein | [M+H]+ | 0.9699 | None |
| M179T610 | 179.143 | 10.17 | C12H20O2 | Dodeca-2(E),4(E)-dienoic acid | [M+H-H2O]+ | 0.7074 | None |
| M180T834_2 | 180.0574 | 13.91 | C11H10ClNO2 | Chlorbufam | [M+H-CO2]+ | 0.9338 | None |
| M180T216 | 180.0627 | 3.6 | C9H9NO3 | Adrenochrome | [M+H]+ | 0.9811 | None |
| M180T145 | 180.0768 | 2.42 | C11H16N4O2 | 2-Nitro-5-(4-methylpiperazino)phenylamine | [M+H-C3H7N]+ | 0.9924 | None |
| M180T207_2 | 180.088 | 3.45 | C7H9N5O | 2-Dimethylamino-6-hydroxypurine | [M+H]+ | 0.9968 | None |
| M180T111 | 180.0994 | 1.85 | C12H17NO4 | 5-(Ethoxycarbonyl)-2,4-dimethyl-1H-pyrrole-3-propanoic acid | [M+H-C2H4O2]+ | 0.9874 | None |
| M180T237_1 | 180.1019 | 3.94 | C10H13NO2 | DL-Homophenylalanine | [M+H]+ | 0.897 | None |
| M180T285 | 180.1019 | 4.75 | C10H13NO2 | N-Acetyltyramine | [M+H]+ | 0.9926 | None |
| M180T603 | 180.1746 | 10.05 | C12H21N | Rimantadine | [M+H]+ | 0.9467 | None |
| M181T239_1 | 181.0495 | 3.99 | C9H8O4 | Caffeic acid | [M+H]+ | 0.9797 | None |
| M181T295 | 181.086 | 4.92 | C11H12O4 | 6-Hydroxy-8-methoxy-3-methylisochroman-1-one | [M+H-CO]+ | 0.8113 | None |
| M181T670 | 181.1223 | 11.16 | C11H20O4 | 3-(1-Hydroxyhexyl)-4-(hydroxymethyl)oxolan-2-one | [M+H-2H2O]+ | 0.9681 | None |
| M181T265 | 181.1336 | 4.42 | C10H16N2O | Rilmenidene | [M+H]+ | 0.9608 | None |
| M182T185_2 | 182.0813 | 3.09 | C9H11NO3 | L-Tyrosine | [M+H]+ | 0.9658 | None |
| M183T370 | 183.0652 | 6.17 | C9H10O4 | Syringaldehyde | [M+H]+ | 0.9937 | None |
| M183T646 | 183.1017 | 10.76 | C13H20O6S | 3-[(2,7-Diketo-10-methyloxecan-5-yl)thio]-2-hydroxypropionic acid | [M+H-C3H6SO3]+ | 0.7784 | None |
| M183T847 | 183.1168 | 14.12 | C16H24O5 | Methyl 2-(4-ethenyl-2,6-dihydroxy-3-(3-hydroxyprop-1-en-2-yl)-4-methylcyclohexyl)prop-2-enoate | [M+H-C2H10O5]+ | 0.8172 | None |
| M183T315 | 183.1493 | 5.24 | C19H38N4O | 1,3-Bis(2,2,6,6-tetramethyl-4-piperidyl)urea | [M+H-C9H20N2]+ | 0.8111 | None |
| M184T801 | 184.0733 | 13.35 | C26H54NO7P | PAF C-16 | [M+H-C21H40O3]+ | 0.9979 | None |
| M184T830 | 184.0734 | 13.84 | C21H46NO4P | Miltefosine | [M+H-C16H32]+ | 0.9954 | None |
| M185T720 | 185.1324 | 12.01 | C15H18O2 | 3,8a-Dimethyl-5-methylene-4a,6,7,8-tetrahydro-4H-benzo[f]benzofuran-2-one | [M+H-CH2O2]+ | 0.9835 | None |
| M186T562 | 186.076 | 9.37 | C8H15NO6 | N-Acetyl-.beta.-D-mannosamine | [M+H-2H2O]+ | 0.7528 | None |
| M186T87_1 | 186.0874 | 1.44 | C12H13NO2 | 3-(Hydroxymethyl)-6,7-dimethylquinolin-2-ol | [M+H-H2O]+ | 0.9706 | None |
| M186T209 | 186.1237 | 3.49 | C8H18N4O2 | L-Arginine ethyl ester | [M+H-NH3]+ | 0.8724 | None |
| M187T608 | 187.1481 | 10.13 | C15H22O3 | Antibiotic JBIR 27 | [M+H-CH4O3]+ | 0.9902 | None |
| M188T198 | 188.0683 | 3.3 | C15H17NO5 | Obscurolide A1 | [M+H-C4H8O3]+ | 0.9263 | None |
| M188T312 | 188.0707 | 5.2 | C11H9NO2 | 3-Indoleacrylic acid | [M+H]+ | 0.8997 | None |
| M189T63 | 189.0871 | 1.06 | C7H12N2O4 | Aceglutamide | [M+H]+ | 0.9858 | None |
| M189T342 | 189.1023 | 5.7 | C12H12N2O2 | 3',4'-Dihydrospiro[imidazolidine-4,1'(2'H)-naphthalene]-2,5-dione | [M+H-CO]+ | 0.9571 | None |
| M189T173 | 189.1234 | 2.88 | C8H16N2O3 | Ile-Gly | [M+H]+ | 0.9982 | None |
| M189T52 | 189.1598 | 0.87 | C9H20N2O2 | N6,N6,N6-Trimethyl-L-lysine | [M+H]+ | 0.9783 | None |
| M189T577 | 189.1637 | 9.62 | C15H22O2 | Polygodial | [M+H-CH2O2]+ | 0.9892 | Into_Blood |
| M189T608 | 189.1637 | 10.13 | C22H34O6 | (1aR,2S,2aS,5R,5aS,6S,7aS)-2-(Acetyloxy)-5-hydroxy-2a,7a-dimethyl-5-(propan-2-yl)decahydroazuleno[5,6-b]oxiren-6-yl (2Z)-2-methylbut-2-enoate | [M+H-C8H14O6]+ | 0.9861 | Into_Blood |
| M190T145 | 190.0711 | 2.42 | C7H11NO5 | N-Acetyl-L-glutamic acid | [M+H]+ | 0.9944 | None |
| M190T541 | 190.0862 | 9.02 | C11H11NO2 | 3-Indolepropionic acid | [M+H]+ | 0.9982 | None |
| M190T951 | 190.0975 | 15.85 | C17H19N3O | Piberaline | [M+H-C7H8]+ | 0.8076 | None |
| M191T457_2 | 191.0702 | 7.62 | C11H10O3 | 4-Methylherniarin | [M+H]+ | 0.9991 | None |
| M191T376 | 191.0703 | 6.27 | C13H18O7 | Sakakin | [M+H-C2H8O4]+ | 0.9871 | None |
| M191T717 | 191.1066 | 11.95 | C12H18O4 | Tuberonic acid | [M+H-2H2O]+ | 0.9093 | None |
| M191T634 | 191.1793 | 10.57 | C14H26O2 | (S)-3-Methyloxacyclotetradecan-2-one | [M+H-2H2O]+ | 0.9578 | Into_Blood |
| M191T601 | 191.1793 | 10.01 | C16H26O2 | Sclareolide | [M+H-C2H4O2]+ | 0.9647 | None |
| M192T334 | 192.0652 | 5.57 | C10H9NO3 | 2-Oxoindole-3-acetate | [M+H]+ | 0.9987 | None |
| M192T277 | 192.0654 | 4.61 | C10H9NO3 | 5-Hydroxyindole-3-acetic acid | [M+H]+ | 0.9982 | None |
| M192T192 | 192.0656 | 3.21 | C10H12N2O3 | L-Kynurenine | [M+H-NH3]+ | 0.9915 | None |
| M192T261 | 192.0686 | 4.35 | C7H13NO3S | N-Acetyl-L-methionine | [M+H]+ | 0.9649 | None |
| M193T113 | 193.0344 | 1.88 | C6H8O7 | Citric acid | [M+H]+ | 0.9526 | None |
| M193T286 | 193.0496 | 4.77 | C12H14O5 | Curvulin | [M+H-C2H6O]+ | 0.9629 | None |
| M193T317 | 193.086 | 5.28 | C11H14O4 | Sinapyl alcohol | [M+H-H2O]+ | 0.9447 | None |
| M193T847 | 193.1224 | 14.12 | C12H18O3 | (.+/-.)7-epi-Jasmonic acid | [M+H-H2O]+ | 0.8824 | None |
| M194T169 | 194.0813 | 2.82 | C14H19NO3 | 3-(3-Methylbut-2-en-1-yl)tyrosine | [M+H-C4H8]+ | 0.8423 | None |
| M194T751 | 194.1177 | 12.51 | C11H15NO2 | Ethyl-4-dimethylaminobenzoate | [M+H]+ | 0.9938 | None |
| M194T241 | 194.1394 | 4.01 | C17H34N6O4 | Val-Ile-Arg | [M+2H]2+ | 0.9164 | None |
| M195T452 | 195.0653 | 7.53 | C10H10O4 | Caffeic acid methylester | [M+H]+ | 0.9233 | None |
| M195T169 | 195.1129 | 2.81 | C10H14N2O2 | (5aR,10aR)-Octahydrodipyrrolo[1,2-a:1',2'-d]pyrazine-5,10-dione | [M+H]+ | 0.966 | None |
| M195T953 | 195.1228 | 15.88 | C8H18O5 | Tetraethylene glycol | [M+H]+ | 0.9936 | None |
| M195T617 | 195.1376 | 10.28 | C12H22O4 | Dodecanedioic acid | [M+H-2H2O]+ | 0.8981 | None |
| M196T226 | 196.0606 | 3.77 | C9H9NO4 | O-Acetylsalicylhydroxamic acid | [M+H]+ | 0.9922 | None |
| M196T378 | 196.2061 | 6.3 | C13H25N | N-(Cyclohexylmethyl)cyclohexanamine | [M+H]+ | 0.9799 | None |
| M197T252 | 197.1286 | 4.21 | C10H19N3O2 | Propenyl-L-NIO | [M+H-NH3]+ | 0.8417 | None |
| M198T449 | 198.149 | 7.48 | C11H19NO2 | L-Proline, 4-cyclohexyl-, (4S)- | [M+H]+ | 0.9247 | None |
| M198T603 | 198.1853 | 10.05 | C19H36N2O3 | CUDA | [M+H-C7H13NO2]+ | 0.7952 | None |
| M199T201_1 | 199.0714 | 3.35 | C13H10O2 | Splitomycin | [M+H]+ | 0.8412 | None |
| M199T127 | 199.1316 | 2.12 | C18H32N6O4 | Ile-His-Lys | [M+2H]2+ | 0.8735 | None |
| M199T717_2 | 199.1326 | 11.96 | C11H20O4 | Homononactinic acid | [M+H-H2O]+ | 0.7397 | None |
| M201T354 | 201.1273 | 5.9 | C14H22O4 | trans-C 75 | [M+H-3H2O]+ | 0.7718 | None |
| M201T436 | 201.1637 | 7.26 | C24H32O4 | Cinnamoylechinadiol | [M+H-C9H12O4]+ | 0.9888 | None |
| M202T186 | 202.0864 | 3.11 | C12H13NO3 | 2-Hydroxy-3-(1H-indol-3-yl)butyric acid | [M+H-H2O]+ | 0.9725 | None |
| M202T236 | 202.0864 | 3.94 | C13H14N2O2 | 1-Methyl-1,2,3,4-tetrahydro-.beta.-carboline-1-carboxylic acid | [M+H-CH3N]+ | 0.9906 | None |
| M202T302 | 202.1801 | 5.03 | C11H23NO2 | 4-Hydroxy-2,2,6,6-tetramethyl-1-piperidineethanol | [M+H]+ | 0.8055 | None |
| M202T573 | 202.2166 | 9.54 | C12H27NO | Ddao | [M+H]+ | 0.9615 | None |
| M203T608 | 203.1794 | 10.13 | C15H26O2 | (1S,4R)-7-Isopropyl-1,4-dimethyl-2,3,3a,5,6,8a-hexahydroazulene-1,4-diol | [M+H-2H2O]+ | 0.992 | Into_Blood |
| M203T436_2 | 203.1794 | 7.26 | C22H30O4 | Ferutinin | [M+H-C7H8O4]+ | 0.9933 | Into_Blood |
| M204T119 | 204.1231 | 1.98 | C9H18NO4+ | O-Acetylcarnitine | [M]+ | 0.9962 | None |
| M204T837 | 204.1384 | 13.96 | C13H17NO | Crotamiton | [M+H]+ | 0.9841 | None |
| M205T62 | 205.082 | 1.03 | C7H12N2O5 | Gly-Glu | [M+H]+ | 0.8327 | None |
| M205T872 | 205.0874 | 14.53 | C12H14O4 | Terephthalic acid, butyl ester | [M+H-H2O]+ | 0.9916 | None |
| M205T242_2 | 205.0972 | 4.04 | C11H12N2O2 | L-Tryptophan | [M+H]+ | 0.9977 | None |
| M205T436_1 | 205.1587 | 7.26 | C15H22O3 | Nardosinon | [M+H-CH2O2]+ | 0.8075 | None |
| M206T220 | 206.0449 | 3.67 | C10H7NO4 | Xanthurenic acid | [M+H]+ | 0.9639 | None |
| M206T210 | 206.1388 | 3.5 | C9H19NO4 | Provitamin B | [M+H]+ | 0.9683 | None |
| M207T265_2 | 207.0653 | 4.41 | C11H12O5 | Sinapic acid | [M+H-H2O]+ | 0.9312 | None |
| M207T362 | 207.1011 | 6.04 | C12H18O5 | p-Methoxytodadiol | [M+H-2H2O]+ | 0.7945 | None |
| M207T436 | 207.1742 | 7.26 | C14H22O | 5-Tetradecynoic acid | [M+H]+ | 0.8501 | None |
| M207T578 | 207.1744 | 9.64 | C14H22O | (1S,5S)-2,4,9-Trimethyl-6-[(E)-prop-1-enyl]-7-oxabicyclo[3.3.1]non-3-ene | [M+H]+ | 0.8516 | Into_Blood |
| M207T807 | 207.1744 | 13.45 | C14H22O | (E)-5-((1S,4R)-3,3-Dimethylbicyclo[2.2.1]heptan-2-yl)pent-3-en-2-one | [M+H]+ | 0.7731 | None |
| M208T365 | 208.0969 | 6.08 | C11H13NO3 | N-Acetyl-L-phenylalanine | [M+H]+ | 0.9965 | None |
| M209T569 | 209.0808 | 9.48 | C11H12O4 | Methyl ferulate | [M+H]+ | 0.994 | None |
| M209T531 | 209.1174 | 8.85 | C12H18O4 | Longicaulenone | [M+H-H2O]+ | 0.9411 | None |
| M209T980_1 | 209.1536 | 16.34 | C13H20O2 | 4,4,7a-Trimethyl-3a,5,6,7-tetrahydro-3H-indene-1-carboxylic acid | [M+H]+ | 0.7328 | None |
| M210T185_1 | 210.0762 | 3.08 | C10H11NO4 | 2-[(2-Methoxy-2-oxoethyl)amino]benzoic acid | [M+H]+ | 0.7423 | None |
| M210T302 | 210.135 | 5.04 | C9H15N5O | Minoxidil | [M+H]+ | 0.8567 | None |
| M211T674 | 211.0866 | 11.23 | C13H10N2O | Oxindole I | [M+H]+ | 0.9821 | None |
| M211T193_2 | 211.1078 | 3.22 | C15H16O2 | 1,2-Diphenylpropane-1,2-diol | [M+H-H2O]+ | 0.9107 | None |
| M211T684 | 211.1329 | 11.4 | C12H20O4 | 4-(5,6-Dihydroxyheptyl)-3-methylfuran-2(5H)-one | [M+H-H2O]+ | 0.9766 | None |
| M211T347 | 211.1441 | 5.78 | C11H18N2O2 | L,L-Cyclo(leucylprolyl) | [M+H]+ | 0.9647 | Into_Blood |
| M211T329 | 211.1442 | 5.49 | C11H18N2O2 | Cyclo(isoleucylprolyl) | [M+H]+ | 0.9551 | Into_Blood |
| M213T324_2 | 213.0659 | 5.4 | C14H10BrFO | 2-Bromo-2-phenyl-1-(4-fluorophenyl)ethanone | [M+H-HBr]+ | 0.9253 | None |
| M213T242 | 213.0757 | 4.03 | C10H12O5 | 2,3-Dihydroxy-1-(4-hydroxy-3-methoxyphenyl)-1-propanone | [M+H]+ | 0.7452 | None |
| M213T218 | 213.1235 | 3.64 | C15H18O2 | Epiligulyl oxide | [M+H-H2O]+ | 0.825 | Into_Blood |
| M213T712 | 213.1638 | 11.87 | C19H28O3 | 16-Hydroxydehydroandrosterone | [M+H-C3H8O3]+ | 0.9857 | None |
| M215T276 | 215.0816 | 4.59 | C14H12N2O3 | N-[4-(4-Nitrophenyl)phenyl]acetamide | [M+H-C2H2O]+ | 0.9416 | None |
| M215T165 | 215.1391 | 2.75 | C10H18N2O3 | Pro-Val | [M+H]+ | 0.9988 | None |
| M216T147 | 216.0977 | 2.45 | C13H13NO2 | TC-E 5008 | [M+H]+ | 0.934 | None |
| M217T279 | 217.0973 | 4.66 | C12H12N2O2 | L-1,2,3,4-Tetrahydro-beta-carboline-3-carboxylic acid | [M+H]+ | 0.8275 | Into_Blood |
| M217T591 | 217.1181 | 9.84 | C9H16N2O4 | Pro-Thr | [M+H]+ | 0.9993 | None |
| M217T127 | 217.1296 | 2.11 | C8H16N4O3 | L-Arginine, N2-acetyl- | [M+H]+ | 0.9426 | None |
| M217T692 | 217.1435 | 11.53 | C11H20O4 | Diethyl pimelate | [M+H]+ | 0.8324 | None |
| M217T183 | 217.1547 | 3.06 | C10H20N2O3 | Val-Val | [M+H]+ | 0.9976 | None |
| M217T634 | 217.1947 | 10.57 | C16H26O | Amberonne | [M+H-H2O]+ | 0.9263 | Into_Blood |
| M217T436_2 | 217.195 | 7.26 | C16H26O | 1-[1,6-dimethyl-4-(4-methylpent-3-enyl)cyclohex-3-en-1-yl]ethanone | [M+H-H2O]+ | 0.8843 | Into_Blood |
| M218T1166 | 218.1388 | 19.43 | C14H27NO4 | tert-Butyl [(4R,6R)-6-(2-aminoethyl)-2,2-dimethyl-1,3-dioxan-4-yl]acetate | [M+H-C4H8]+ | 0.922 | None |
| M218T168 | 218.1388 | 2.81 | C10H19NO4 | L-Propionylcarnitine | [M+H]+ | 0.9956 | None |
| M219T376 | 219.0652 | 6.27 | C19H24O7 | (2E,5R,6R,11S)-5,6,15-Trihydroxy-17-methoxy-11-methyl-12-oxabicyclo[12.4.0]octadeca-1(14),2,15,17-tetraene-7,13-dione | [M+H-C7H14O3]+ | 0.9245 | Into_Blood |
| M219T70 | 219.0716 | 1.17 | C11H10N2O3 | 4-(5-Keto-3-methyl-3-pyrazolin-1-yl)benzoic acid | [M+H]+ | 0.9899 | None |
| M219T85 | 219.0976 | 1.42 | C8H14N2O5 | .gamma.-L-Glutamyl-L-alanine | [M+H]+ | 0.9616 | None |
| M219T326 | 219.1016 | 5.43 | C14H18O4 | Coenzyme Q1 | [M+H-CH4O]+ | 0.7075 | None |
| M219T307 | 219.1129 | 5.12 | C12H14N2O2 | N-Acetyl-5-hydroxytryptamine | [M+H]+ | 0.9964 | Into_Blood |
| M219T208 | 219.1336 | 3.46 | C9H18N2O4 | Ser-Leu | [M+H]+ | 0.9877 | None |
| M219T419 | 219.1382 | 6.99 | C32H39NO3 | Hydroxyebastine | [M+H-C18H21NO]+ | 0.8097 | None |
| M219T837_1 | 219.1744 | 13.95 | C15H22O | Germacrone | [M+H]+ | 0.9263 | None |
| M219T539 | 219.1744 | 8.98 | C15H24O2 | 2,6-Di-tert-butyl-4-hydroxymethylphenol | [M+H-H2O]+ | 0.9947 | None |
| M220T211 | 220.118 | 3.51 | C9H17NO5 | Pantothenate | [M+H]+ | 0.9896 | None |
| M221T513 | 221.0807 | 8.55 | C18H18O7 | 5,7-Dihydroxy-3',4',5'-trimethoxyflavanone | [M+H-C6H6O3]+ | 0.9425 | None |
| M221T172 | 221.0921 | 2.87 | C8H16N2O3S | Cys-Val | [M+H]+ | 0.7215 | None |
| M221T436 | 221.1899 | 7.26 | C15H26O2 | ((1S)-5-Methyl-1-(4-methylpent-3-en-1-yl)-2-oxabicyclo[2.2.2]octan-4-yl)methanol | [M+H-H2O]+ | 0.9742 | None |
| M221T661 | 221.19 | 11.02 | C15H28O3 | 2-(1-Hydroxy-1-methylethyl)-4a,8-dimethyldecalin-1,5-diol | [M+H-2H2O]+ | 0.9356 | None |
| M223T51 | 223.0242 | 0.85 | C10H8N4O3S | N-[5-(4-Nitrophenyl)-1,3,4-thiadiazol-2-yl]acetamide | [M+H-C2H2O]+ | 0.999 | None |
| M223T202 | 223.1078 | 3.37 | C11H14N2O3 | Phe-Gly | [M+H]+ | 0.998 | None |
| M223T573 | 223.1329 | 9.55 | C13H20O4 | 3-(Hepta-1,3-dienyl)adipic acid | [M+H-H2O]+ | 0.9032 | None |
| M223T814 | 223.1481 | 13.56 | C19H22O5 | Gibberellin A7 | [M+H-C2H4O5]+ | 0.9651 | None |
| M223T279 | 223.1541 | 4.65 | C10H22O5 | Tetraglyme | [M+H]+ | 0.9225 | None |
| M224T205 | 224.0918 | 3.41 | C11H13NO4 | Phenylalanine, 4-(carboxymethyl)- | [M+H]+ | 0.9493 | None |
| M224T255 | 224.0919 | 4.25 | C11H13NO4 | N-Acetyl-L-tyrosine | [M+H]+ | 0.9897 | None |
| M225T721 | 225.1487 | 12.01 | C13H20O3 | 3-Heptyl-3,6-dihydro-1H-furo[3,4-c]furan-4-one | [M+H]+ | 0.9938 | None |
| M225T849 | 225.1637 | 14.15 | C26H40O5 | Simvastatin 4'-methyl ether | [M+H-C9H20O5]+ | 0.9577 | None |
| M226T255 | 226.1368 | 4.25 | C21H34N6O5 | Tyr-Leu-Arg | [M+2H]2+ | 0.8317 | None |
| M226T181 | 226.137 | 3.02 | C21H34N6O5 | Leu-Tyr-Arg | [M+2H]2+ | 0.8472 | None |
| M227T624_1 | 227.0703 | 10.41 | C14H10O3 | NSC 6646 | [M+H]+ | 0.9965 | None |
| M227T206 | 227.1027 | 3.44 | C10H14N2O4 | PyroGlu-Pro | [M+H]+ | 0.7743 | None |
| M229T506 | 229.0859 | 8.43 | C17H19NO3 | Galanthaminone | [M+H-C3H7N]+ | 0.9636 | None |
| M229T311_1 | 229.0859 | 5.18 | C14H12O3 | Resveratrol | [M+H]+ | 0.9896 | None |
| M229T244 | 229.1183 | 4.07 | C10H16N2O4 | PyroGlu-Val | [M+H]+ | 0.8157 | None |
| M229T436 | 229.195 | 7.26 | C18H26O2 | Empenthrin | [M+H-CH2O2]+ | 0.9577 | None |
| M230T167_2 | 230.1136 | 2.78 | C9H15N3O4 | Pro-Asn | [M+H]+ | 0.9861 | None |
| M230T663_2 | 230.2477 | 11.05 | C14H31NO | N,N-Dimethyldodecylamine N-oxide | [M+H]+ | 0.9874 | None |
| M231T44 | 231.035 | 0.73 | C6H6N4O6 | 1H-Pyrazole-1-acetic acid, 5-methyl-3,4-dinitro- | [M+H]+ | 0.8052 | None |
| M232T188 | 232.021 | 3.13 | C11H7N5O2S | 8-Nitropyrimido[4,5-b][1,4]benzothiazepin-4-ylamine | [M+H-CH2N2]+ | 0.8885 | None |
| M232T454 | 232.1079 | 7.57 | C10H15N3O2 | 4-(Methylnitrosamino)-1-(3-pyridyl)-1-butanol | [M+Na]+ | 0.8917 | None |
| M233T394 | 233.0796 | 6.56 | C19H22O9 | 7-Acetyl-3,8-dihydroxy-6-methylnaphthalen-1-yl .beta.-D-glucopyranoside | [M+H-C6H10O5]+ | 0.9566 | Into_Blood |
| M233T418 | 233.0796 | 6.97 | C13H14O5 | 3-(2-Hydroxypropyl)-8-hydroxy-6-methoxy-2H-1-benzopyran-2-one | [M-H2O+H]+ | 0.8988 | Into_Blood |
| M233T206 | 233.1496 | 3.44 | C10H20N2O4 | Thr-Leu | [M+H]+ | 0.976 | None |
| M233T696 | 233.1536 | 11.6 | C15H22O3 | NCGC00385483-01 | [M+H-H2O]+ | 0.9339 | None |
| M233T681 | 233.1536 | 11.36 | C15H20O2 | Costunolide | [M+H]+ | 0.9063 | None |
| M234T298 | 234.0738 | 4.97 | C12H11NO4 | 6-Ethoxy-4-hydroxy-3-quinolinecarboxylic acid | [M+H]+ | 0.9974 | Into_Blood |
| M235T364 | 235.06 | 6.06 | C12H10O5 | 7-Methoxycoumarin-4-acetic acid | [M+H]+ | 0.9877 | None |
| M235T350_2 | 235.0601 | 5.83 | C12H10O5 | 7-Hydroxy-4-methylcoumarin-3-acetic acid | [M+H]+ | 0.9688 | Into_Blood |
| M235T394 | 235.0955 | 6.57 | C13H14O4 | 7-Hydroxy-3-(2-hydroxypropyl)-5-methylisocoumarin | [M+H]+ | 0.9898 | Into_Blood |
| M235T148 | 235.119 | 2.47 | C11H16N4O3 | His-Pro | [M+H-H2O]+ | 0.9947 | None |
| M235T839 | 235.1685 | 13.98 | C15H22O2 | 3,5-Di-tert-butylsalicylaldehyde | [M+H]+ | 0.9808 | None |
| M235T601 | 235.1692 | 10.01 | C15H22O2 | Lychnophoic acid | [M+H]+ | 0.9647 | None |
| M235T739 | 235.1693 | 12.32 | C24H32O5 | 10-Hydroxy-1,6-dimethyl-9-(propan-2-yl)-5,12-dioxatricyclo[9.1.0.04,6]dodecan-8-yl (2E)-3-phenylprop-2-enoate | [M+H-C9H10O3]+ | 0.9306 | None |
| M235T675 | 235.1693 | 11.24 | C15H24O3 | Ageratriol | [M+H-H2O]+ | 0.906 | None |
| M237T194_2 | 237.0871 | 3.24 | C17H17NO2 | Apomorphine | [M+H-CH5N]+ | 0.8272 | None |
| M237T244 | 237.1234 | 4.07 | C12H16N2O3 | Ala-Phe | [M+H]+ | 0.9865 | None |
| M237T213_2 | 237.1234 | 3.55 | C12H16N2O3 | Phe-Ala | [M+H]+ | 0.9979 | None |
| M237T675 | 237.1849 | 11.24 | C15H28O4 | 6-Hydroxy-6-(3-hydroxy-2,3-dimethylcyclopentyl)-2-methylenanthic acid | [M+H-2H2O]+ | 0.9463 | None |
| M237T770 | 237.185 | 12.83 | C15H26O3 | NCGC00381166-01 | [M-H2O+H]+ | 0.8021 | None |
| M237T809 | 237.2213 | 13.49 | C17H32O2 | Methyl palmitelaidate | [M+H-CH4O]+ | 0.9357 | None |
| M239T1045 | 239.149 | 17.42 | C10H22O6 | Pentaethylene glycol | [M+H]+ | 0.9667 | None |
| M241T413 | 241.0706 | 6.89 | C9H14O6 | Vanay | [M+Na]+ | 0.9813 | None |
| M241T310_1 | 241.0859 | 5.16 | C23H22O6 | Rotenone | [M+H-C8H10O3]+ | 0.9728 | None |
| M241T813 | 241.1586 | 13.56 | C17H22O2 | 3-Hexen-2-one, 5-[2-(3-hydroxy-1-propen-1-yl)-4-methylphenyl]-3-methyl- | [M+H-H2O]+ | 0.9233 | None |
| M241T712 | 241.195 | 11.87 | C21H32O2 | 3,20-Allopregnanedione | [M+H-C3H8O2]+ | 0.9747 | None |
| M242T772 | 242.2843 | 12.87 | C16H35N | 1-Hexadecanamine | [M+H]+ | 0.9955 | None |
| M243T193 | 243.0977 | 3.22 | C10H14N2O5 | Thymidine | [M+H]+ | 0.9211 | None |
| M243T308 | 243.134 | 5.14 | C11H18N2O4 | 2-Piperazinepropionic acid, 5-isobutyl-3,6-dioxo- | [M+H]+ | 0.9705 | Into_Blood |
| M243T848 | 243.1744 | 14.13 | C17H24O2 | Falcarindiol | [M+H-H2O]+ | 0.9187 | None |
| M243T436 | 243.2107 | 7.26 | C19H32O2 | Methyl linolenate | [M+H-CH6O2]+ | 0.9828 | None |
| M244T128_1 | 244.0928 | 2.13 | C9H13N3O5 | Cytidine | [M+H]+ | 0.9998 | None |
| M244T197 | 244.1293 | 3.28 | C10H17N3O4 | Pro-Gln | [M+H]+ | 0.9321 | None |
| M245T646 | 245.0763 | 10.77 | C15H12O5 | Microminutin | [M+H-CO]+ | 0.9715 | None |
| M245T202 | 245.1133 | 3.36 | C10H16N2O5 | Pro-Glu | [M+H]+ | 0.972 | None |
| M245T790 | 245.1167 | 13.17 | C15H16O3 | Osthole | [M+H]+ | 0.9938 | None |
| M245T374 | 245.1286 | 6.23 | C14H18N2O3 | DL-Prolylphenylalanine | [M+H-H2O]+ | 0.949 | None |
| M245T389 | 245.1384 | 6.49 | C12H22O6 | 9-Glyceryloxy-9-ketopelargonic acid | [M+H-H2O]+ | 0.7694 | None |
| M245T282 | 245.1859 | 4.69 | C12H24N2O3 | Ile-Ile | [M+H]+ | 0.9995 | None |
| M245T293 | 245.1861 | 4.89 | C12H24N2O3 | Ile-Leu | [M+H]+ | 0.9975 | None |
| M247T506 | 247.0963 | 8.43 | C20H24O9 | Torachrysone 8-glucoside | [M+H-C6H10O5]+ | 0.9323 | None |
| M247T384 | 247.1077 | 6.41 | C13H14N2O3 | N-acetyltryptophan | [M+H]+ | 0.9525 | None |
| M247T237 | 247.129 | 3.95 | C10H18N2O5 | Asp-Leu | [M+H]+ | 0.8287 | None |
| M247T222 | 247.129 | 3.7 | C10H18N2O5 | Asp-Ile | [M+H]+ | 0.9609 | None |
| M247T132 | 247.1402 | 2.19 | C9H18N4O4 | D-Octopine | [M+H]+ | 0.827 | Into_Blood |
| M247T601 | 247.1688 | 10.01 | C16H24O3 | Brefeldin C | [M+H-H2O]+ | 0.8528 | None |
| M248T223_2 | 248.1394 | 3.72 | C13H17N3O2 | 12-Cytisineacetamide | [M+H]+ | 0.9623 | None |
| M248T159_2 | 248.1493 | 2.64 | C11H21NO5 | 3-Hydroxybutyrylcarnitine | [M+H]+ | 0.979 | None |
| M249T461 | 249.1121 | 7.69 | C14H16O4 | 8-Hydroxy-3-(4-ketopentyl)isochroman-1-one | [M+H]+ | 0.8126 | None |
| M249T362_1 | 249.1122 | 6.03 | C14H16O4 | 7,8-Dihydrokawain-5-ol | [M+H]+ | 0.933 | None |
| M249T261 | 249.1235 | 4.35 | C22H25N3O3 | Dacinostat | [M+H-C9H9N]+ | 0.7388 | None |
| M249T615 | 249.1485 | 10.26 | C16H24O7 | 4-Dec-9-enyl-3-hydroxy-5-oxooxolane-2,3-dicarboxylic acid | [M+H-CH4O4]+ | 0.9285 | None |
| M249T840 | 249.1836 | 14.01 | C20H32O2 | Methyl 2,4,6-tri-tert-butylbenzoate | [M+H-C4H8]+ | 0.973 | None |
| M249T711 | 249.206 | 11.86 | C13H28O4 | Tri(propylene glycol) butyl ether | [M+H]+ | 0.9983 | None |
| M250T670 | 250.1776 | 11.17 | C15H23NO2 | Alprenolol | [M+H]+ | 0.9991 | None |
| M251T764 | 251.1642 | 12.73 | C15H22O3 | 3,5-Di-tert-butyl-4-hydroxybenzoic acid | [M+H]+ | 0.9311 | None |
| M251T819 | 251.1642 | 13.66 | C15H22O3 | (4aR,5S)-9-Hydroxy-3,4a,5-trimethyl-4,5,6,7,8,8a,9,9a-octahydrobenzo[f][1]benzofuran-2-one | [M+H]+ | 0.9769 | None |
| M251T675 | 251.1643 | 11.26 | C15H22O3 | Pentalenic acid | [M+H]+ | 0.9527 | None |
| M252T207 | 252.0728 | 3.45 | C14H18N5O11P | Adenylsuccinic acid | [M+H-C5H9O7P]+ | 0.9495 | None |
| M252T257 | 252.0844 | 4.28 | C11H13N3O2S | Protein Kinase Inhibitor H-9 | [M+H]+ | 0.9993 | None |
| M252T161 | 252.1092 | 2.68 | C10H13N5O3 | 2'-Deoxyadenosine | [M+H]+ | 0.9994 | None |
| M253T180 | 253.1183 | 2.99 | C12H16N2O4 | Phe-Ser | [M+H]+ | 0.9618 | None |
| M254T502 | 254.0813 | 8.36 | C15H11NO3 | Furegrelate | [M+H]+ | 0.9971 | None |
| M254T673 | 254.2478 | 11.22 | C16H31NO | 1-Lauryl-2-pyrrolidone | [M+H]+ | 0.9565 | None |
| M255T520 | 255.064 | 8.67 | C15H10O4 | Chrysophanol | [M+H]+ | 0.9967 | Into_Blood |
| M255T393 | 255.064 | 6.56 | C15H10O4 | 4'-Hydroxyflavonol | [M+H]+ | 0.9997 | Into_Blood |
| M255T436 | 255.2106 | 7.26 | C20H32O4 | 2,6,10-Dodecatrienoic acid, 12-hydroxy-10-(hydroxymethyl)-6-methyl-2-(4-methyl-3-pentenyl)-, (E,Z,E)- | [M+H-CH6O4]+ | 0.9851 | None |
| M255T720 | 255.2107 | 12.01 | C25H38O8 | Epiandrosterone glucuronide | [M+H-C6H12O8]+ | 0.9756 | None |
| M255T809 | 255.2318 | 13.49 | C16H30O2 | Palmitoleic acid | [M+H]+ | 0.9485 | None |
| M256T357 | 256.0737 | 5.95 | C13H9N3O3 | 5-(3-Indolylmethylene)barbituric acid | [M+H]+ | 0.9818 | None |
| M256T849 | 256.2051 | 14.15 | C18H25N | 5-(4-Pyridinyl)-5-(2-butenyl)-2,7-nonadiene | [M+H]+ | 0.9395 | None |
| M256T943 | 256.2633 | 15.71 | C16H33NO | Palmitamide | [M+H]+ | 0.9984 | None |
| M256T751 | 256.3 | 12.51 | C17H38N | Tetradonium cation | [Cat]+ | 0.993 | None |
| M257T350 | 257.0409 | 5.84 | C14H8O5 | Purpurin | [M+H]+ | 0.9983 | Into_Blood |
| M257T390_2 | 257.0811 | 6.49 | C15H12O4 | Dihydrodaidzein | [M+H]+ | 0.929 | None |
| M257T847_1 | 257.1901 | 14.12 | C18H26O2 | Epinandrolone | [M+H-H2O]+ | 0.9147 | None |
| M257T634 | 257.2262 | 10.57 | C20H30O2 | Neoabietic acid | [M+H-CH2O2]+ | 0.9692 | None |
| M258T56 | 258.1102 | 0.94 | C8H21NO6P | sn-Glycero-3-phosphocholine | [M]+ | 0.9811 | None |
| M258T301 | 258.1125 | 5.02 | C15H17NO4 | Dubinidine | [M+H-H2O]+ | 0.9297 | Into_Blood |
| M258T161 | 258.1338 | 2.68 | C11H19F2NO2 | tert-Butyl 4,4-difluorocyclohexylcarbamate | [M+Na]+ | 0.8246 | None |
| M258T839 | 258.2424 | 13.98 | C15H31NO2 | D-erythro-Sphingosine C-15 | [M+H]+ | 0.8525 | None |
| M258T716 | 258.2791 | 11.94 | C16H35NO | Myristamine oxide | [M+H]+ | 0.9914 | None |
| M259T84 | 259.0926 | 1.41 | C10H14N2O6 | Ribothymidine | [M+H]+ | 0.9137 | None |
| M259T813 | 259.1693 | 13.56 | C18H28O4 | Albocycline | [M+H-CH6O2]+ | 0.8849 | None |
| M259T698 | 259.1693 | 11.63 | C26H40O2 | L-759633 | [M+H-C9H18]+ | 0.7086 | Into_Blood |
| M259T743 | 259.1693 | 12.38 | C21H30O2 | (.+/-.)-.DELTA.9-THC | [M+H-C4H8]+ | 0.8326 | None |
| M260T378 | 260.1857 | 6.3 | C13H25NO4 | (R)-Caproylcarnitine | [M+H]+ | 0.9911 | None |
| M261T267 | 261.1227 | 4.45 | C14H16N2O3 | Cyclo(prolyltyrosyl) | [M+H]+ | 0.9622 | None |
| M261T250 | 261.1446 | 4.17 | C11H20N2O5 | L-.gamma.-Glutamyl-L-leucine | [M+H]+ | 0.9621 | None |
| M261T628 | 261.1485 | 10.46 | C16H22O4 | 7-Dehydrobrefeldin A | [M+H-H2O]+ | 0.8017 | None |
| M261T847 | 261.1848 | 14.12 | C15H26O2 | Jaeskeanadiol | [M+Na]+ | 0.7114 | None |
| M262T319 | 262.1544 | 5.32 | C9H19N5O4 | Arg-Ser | [M+H]+ | 0.9578 | None |
| M262T341 | 262.1538 | 5.68 | C19H19N | N-Desmethylcyclobenzaprine | [M+H]+ | 0.9824 | None |
| M263T863 | 263.2006 | 14.39 | C17H28O3 | 12(S)-HHTrE | [M+H-H2O]+ | 0.7732 | None |
| M263T912 | 263.237 | 15.2 | C37H68O5 | 1-Palmitoyl-2-linoleoyl-rac-glycerol | [M+H-C19H38O4]+ | 0.9784 | None |
| M263T839 | 263.237 | 13.99 | C20H35NO3 | Linoleoylglycine | [M+H-C2H5NO2]+ | 0.9779 | None |
| M264T64_2 | 264.1338 | 1.06 | C13H17N3O3 | Toluene-2,4,6-triamine, N,N',N''-triacetyl- | [M+H]+ | 0.8827 | None |
| M265T66 | 265.1118 | 1.1 | C12H17N4OS | vitamin B1 | [Cat]+ | 0.998 | None |
| M265T464 | 265.1433 | 7.74 | C15H22O5 | 3-((3aS,6S,8aR)-8-Hydroxy-6,8-dimethyl-3-methylidene-2-oxooctahydro-2H-cyclohepta[b]furan-7-yl)propanoic acid | [M+H-H2O]+ | 0.8339 | None |
| M265T252_2 | 265.1435 | 4.2 | C21H32O10 | 1-O-((2E,4E)-5-((1R,3S,5S,8S)-3,8-Dihydroxy-1,5-dimethyl-6-oxabicyclo[3.2.1]octan-8-yl)-3-methylpenta-2,4-dienoyl)-.beta.-D-glucopyranose | [M+H-C6H12O6]+ | 0.8 | None |
| M265T277 | 265.1547 | 4.61 | C14H20N2O3 | Valylphenylalanine | [M+H]+ | 0.9952 | None |
| M265T957 | 265.2526 | 15.94 | C20H37NO3 | N-Oleoylglycine | [M+H-C2H5NO2]+ | 0.9601 | None |
| M266T58 | 266.1598 | 0.97 | C17H19N3 | 1-Methyl-4-(1-methylpyrrolidin-2-yl)-9H-b-carboline | [M+H]+ | 0.9301 | None |
| M266T816 | 266.1903 | 13.6 | C19H23N | Diphenidine | [M+H]+ | 0.9606 | None |
| M267T105 | 267.0589 | 1.75 | C12H11FN2O2S | N-(3-Aminophenyl)-4-fluorobenzenesulfonamide | [M+H]+ | 0.9989 | None |
| M267T187 | 267.134 | 3.11 | C13H18N2O4 | Phe-Thr | [M+H]+ | 0.9969 | None |
| M267T249 | 267.134 | 4.14 | C13H18N2O4 | Thr-Phe | [M+H]+ | 0.9655 | Into_Blood |
| M267T827 | 267.172 | 13.78 | C12H27O4P | Tributyl phosphate | [M+H]+ | 0.9996 | None |
| M269T164 | 269.0882 | 2.73 | C10H12N4O5 | Inosine | [M+H]+ | 0.9931 | None |
| M269T215 | 269.1496 | 3.59 | C18H20O2 | Equilin | [M+H]+ | 0.9138 | None |
| M269T146 | 269.1608 | 2.44 | C12H20N4O3 | His-Ile | [M+H]+ | 0.988 | None |
| M269T436 | 269.2262 | 7.26 | C20H34O3 | 1,6,10,14-Phytatetraene-3,5,9-triol | [M+H-3H2O]+ | 0.9889 | None |
| M270T477 | 270.0886 | 7.95 | C13H22N4O3S | Ranitidine | [M+H-C2H7N]+ | 0.9639 | None |
| M270T57 | 270.1184 | 0.94 | C15H17N3O3 | 2-[(2-Butoxypyrimidin-4-yl)amino]benzoic acid | [M+H-H2O]+ | 0.9563 | None |
| M271T508 | 271.0588 | 8.47 | C15H10O5 | Emodol | [M+H]+ | 0.9945 | Into_Blood |
| M271T556 | 271.0593 | 9.26 | C15H10O5 | 3'-Hydroxydaidzein | [M+H]+ | 0.9023 | None |
| M271T341 | 271.0601 | 5.69 | C15H10O5 | 3',7-Dihydroxyflavonol | [M+H]+ | 0.8465 | Into_Blood |
| M271T398 | 271.0601 | 6.63 | C15H10O5 | Baicalein | [M+H]+ | 0.9973 | Into_Blood |
| M271T301 | 271.0602 | 5.02 | C15H10O5 | 6,7,3'-Trihydroxyflavone | [M+H]+ | 0.8513 | Into_Blood |
| M271T369 | 271.0603 | 6.14 | C15H10O5 | Aloe emodin | [M+H]+ | 0.8433 | Into_Blood |
| M271T730 | 271.2415 | 12.16 | C20H34O2 | Incensol | [M+H-2H2O]+ | 0.9632 | None |
| M271T634_2 | 271.2416 | 10.57 | C20H34O2 | Homo-.alpha.-linolenic acid | [M+H-2H2O]+ | 0.9628 | None |
| M272T179 | 272.103 | 2.98 | C12H14F3N3O2 | 1-[4-Methyl-6-(trifluoromethyl)pyrimidin-2-yl]nipecotic acid | [M+H-H2O]+ | 0.9203 | None |
| M273T296 | 273.0755 | 4.93 | C22H18O10 | (-)-Catechin gallate | [M+H-C7H6O5]+ | 0.9804 | None |
| M273T400 | 273.0758 | 6.66 | C15H12O5 | Naringenin chalcone | [M+H]+ | 0.9985 | Into_Blood |
| M273T695 | 273.1484 | 11.58 | C15H22O3 | 2-((1S,2S,4aR,8aS)-1-hydroxy-4a-methyl-8-methylenedecahydronaphthalen-2-yl)acrylic acid | [M+Na]+ | 0.9765 | None |
| M275T421 | 275.0905 | 7.02 | C15H14O5 | Phloretin | [M+H]+ | 0.9795 | Into_Blood |
| M275T391 | 275.0914 | 6.52 | C21H24O10 | Symposide | [M+H-C6H10O5]+ | 0.9392 | None |
| M275T67_2 | 275.1353 | 1.12 | C14H18N4S | N-[2-(1H-Imidazol-5-yl)ethyl]-N'-(2-phenylethyl)thiourea | [M+H]+ | 0.8833 | None |
| M275T766 | 275.2006 | 12.77 | C19H34O5 | Methyl (9Z,14Z)-12,13,16-trihydroxyoctadeca-9,14-dienoate | [M+H-CH8O3]+ | 0.9471 | None |
| M275T818 | 275.2006 | 13.64 | C18H28O3 | 9-OxoOTrE | [M+H-H2O]+ | 0.9545 | None |
| M275T785 | 275.2006 | 13.08 | C18H32O5 | 12,15-Octadecadienoic acid, 7,9,10-trihydroxy- | [M+H-3H2O]+ | 0.947 | Into_Blood |
| M276T64 | 276.1191 | 1.07 | C10H17N3O6 | SCHEMBL19374 | [M+H]+ | 0.9252 | None |
| M276T274 | 276.1344 | 4.56 | C14H17N3O3 | Ala-Trp | [M+H]+ | 0.9486 | None |
| M277T352 | 277.1071 | 5.87 | C20H24O7 | 6-Hydroxy-10a-methyl-4,7-dimethylidene-3,8-dioxododecahydrooxireno[8,9]cyclodeca[1,2-b]furan-5-yl (2Z)-2-methylbut-2-enoate | [M+H-C5H8O2]+ | 0.9087 | None |
| M277T387 | 277.1072 | 6.46 | C15H16O5 | Visamminol | [M+H]+ | 0.7281 | None |
| M277T334 | 277.1184 | 5.56 | C14H16N2O4 | PyroGlu-Phe | [M+H]+ | 0.9163 | None |
| M277T760 | 277.2149 | 12.67 | C18H30O3 | 9-Oxo-10(E),12(E)-octadecadienoic acid | [M+H-H2O]+ | 0.9707 | None |
| M279T346 | 279.0856 | 5.77 | C14H14O6 | Methyl 6,7-dimethoxycoumarin-4-acetate | [M+H]+ | 0.9103 | None |
| M279T192_2 | 279.1011 | 3.2 | C10H18N2O5S | gamma-Glutamylmethionine | [M+H]+ | 0.9527 | None |
| M279T218 | 279.134 | 3.63 | C14H18N2O4 | Tyr-Pro | [M+H]+ | 0.9631 | None |
| M279T192_3 | 279.134 | 3.2 | C14H18N2O4 | Pro-Tyr | [M+H]+ | 0.9974 | None |
| M279T872 | 279.1584 | 14.53 | C16H22O4 | Mono-2-ethylhexyl phthalate | [M+H]+ | 0.9942 | None |
| M279T656_1 | 279.1592 | 10.93 | C16H22O4 | .alpha.-CEHC | [M+H]+ | 0.9926 | None |
| M279T300 | 279.1703 | 5 | C15H22N2O3 | Ile-Phe | [M+H]+ | 0.9971 | None |
| M279T826_2 | 279.2314 | 13.77 | C18H30O2 | (9Z,12Z,15Z)-Octadecatrienoic acid | [M+H]+ | 0.9902 | None |
| M279T849 | 279.2318 | 14.15 | C18H34O4 | Oxiraneoctanoic acid, .eta.-hydroxy-3-octyl- | [M+H-2H2O]+ | 0.9262 | None |
| M280T500 | 280.152 | 8.34 | C16H17N5 | MLS000061860 | [M+H]+ | 0.9987 | None |
| M281T457 | 281.0446 | 7.61 | C16H10O6 | 3,8-Dihydroxy-1-methylanthraquinone-2-carboxylic acid | [M+H-H2O]+ | 0.9341 | Only_Into_Blood |
| M281T253 | 281.1134 | 4.22 | C13H16N2O5 | Aspartylphenylalanine | [M+H]+ | 0.9385 | None |
| M281T212 | 281.1495 | 3.53 | C14H20N2O4 | Val-Tyr | [M+H]+ | 0.985 | None |
| M282T814 | 282.1852 | 13.56 | C19H23NO | Diphenylpyraline | [M+H]+ | 0.9774 | None |
| M282T956_2 | 282.2788 | 15.94 | C18H35NO | Oleamide | [M+H]+ | 0.9662 | None |
| M283T776 | 283.0964 | 12.94 | C17H14O4 | 3-Benzyl-4-hydroxy-5-(4-hydroxyphenyl)furan-2(5H)-one | [M+H]+ | 0.939 | None |
| M283T251 | 283.1267 | 4.18 | C19H22O6 | Gibberellin A3 | [M+H-CH4O3]+ | 0.906 | None |
| M283T931_1 | 283.1706 | 15.52 | C21H26O4 | 1-Acetyl-10a,12a-dimethyl-4,4a,4b,5,6,10b,11,12-octahydro-1H-naphtho[2,1-f]isochromene-3,8-dione | [M+H-C2H4O2]+ | 0.8559 | None |
| M283T436 | 283.2418 | 7.26 | C21H36O3 | Pregnanetriol | [M+H-3H2O]+ | 0.9754 | None |
| M283T876_2 | 283.2632 | 14.6 | C18H34O2 | Oleic acid | [M+H]+ | 0.9512 | None |
| M284T410 | 284.0552 | 6.83 | C12H11ClFN3O2 | Ethyl 1-(5-chloro-3-fluoropyridin-2-yl)-5-methylpyrazole-4-carboxylate | [M+H]+ | 0.9971 | Into_Blood |
| M284T163_2 | 284.1014 | 2.72 | C10H13N5O5 | Guanosine | [M+H]+ | 0.9987 | None |
| M284T463 | 284.1281 | 7.72 | C17H17NO3 | Coumaroyl tyramine | [M+H]+ | 0.9656 | None |
| M284T392 | 284.1394 | 6.54 | C16H17N3O2 | Brevianamide F | [M+H]+ | 0.9983 | Into_Blood |
| M284T1021 | 284.2942 | 17.02 | C18H37NO | Stearamide | [M+H]+ | 0.9962 | None |
| M285T349 | 285.0393 | 5.81 | C15H8O6 | Rhein | [M+H]+ | 0.9808 | Into_Blood |
| M285T698 | 285.0743 | 11.63 | C16H12O5 | Oroxylin | [M+H]+ | 0.9961 | None |
| M285T713 | 285.0757 | 11.89 | C16H12O5 | Wogonin | [M+H]+ | 0.9829 | None |
| M285T175_1 | 285.0831 | 2.92 | C10H12N4O6 | Xanthosine | [M+H]+ | 0.9963 | None |
| M285T634 | 285.2575 | 10.57 | C21H36O2 | Pregnanediol | [M+H-2H2O]+ | 0.9521 | None |
| M285T730 | 285.2575 | 12.16 | C21H36O2 | Allopregnanediol | [M+H-2H2O]+ | 0.9484 | None |
| M286T863 | 286.2165 | 14.39 | C19H27NO | 1-Methyl-2-nonyl-4(1H)-quinolinone | [M+H]+ | 0.965 | None |
| M287T343 | 287.0544 | 5.71 | C15H10O6 | Kaempferol | [M+H]+ | 0.9994 | Into_Blood |
| M287T508 | 287.0549 | 8.46 | C15H10O6 | Citreorosein | [M+H]+ | 0.9832 | None |
| M287T316 | 287.0551 | 5.27 | C21H20O10 | Afzelin | [M+H-C6H10O4]+ | 0.9992 | None |
| M287T157 | 287.0763 | 2.62 | C19H25N8O11P | Adenylyl(3'-5')cytidine | [M+2H]2+ | 0.9965 | None |
| M287T719 | 287.0858 | 11.99 | C16H14O5 | Licochalcone B | [M+H]+ | 0.9286 | None |
| M287T742 | 287.0859 | 12.36 | C16H14O5 | Sakuranetin | [M+H]+ | 0.9063 | None |
| M287T251 | 287.2078 | 4.19 | C19H28O3 | 11.alpha.-Hydroxytestosterone | [M+H-H2O]+ | 0.8144 | None |
| M287T864 | 287.2369 | 14.39 | C23H38O4 | 1-Arachidonoylglycerol | [M+H-C3H8O3]+ | 0.9688 | None |
| M288T758 | 288.2533 | 12.64 | C16H33NO3 | Amycol LDE | [M+H]+ | 0.9772 | None |
| M289T499 | 289.0705 | 8.31 | C15H12O6 | Eriodictyol | [M+H]+ | 0.9929 | None |
| M289T82 | 289.0917 | 1.37 | C12H22O11 | Palatinose | [M+H-3H2O]+ | 0.9324 | None |
| M289T197 | 289.0918 | 3.28 | C12H16O8 | Dianthoside | [M+H]+ | 0.9971 | Into_Blood |
| M289T365 | 289.108 | 6.09 | C13H12F2N6O | Fluconazole | [M+H-H2O]+ | 0.9987 | Into_Blood |
| M291T371 | 291.0974 | 6.18 | C14H14N2O5 | N-Malonyltryptophan | [M+H]+ | 0.8336 | None |
| M291T127_1 | 291.1298 | 2.11 | C10H18N4O6 | Argininosuccinic acid | [M+H]+ | 0.8612 | None |
| M291T628 | 291.1589 | 10.46 | C17H24O5 | Inulicin | [M+H-H2O]+ | 0.9286 | None |
| M291T681 | 291.1954 | 11.35 | C20H30O5 | 4-Acetoxy-8-(3-keto-2-pent-2-enylcyclopenten-1-yl)caprylic acid | [M+H-C2H4O2]+ | 0.879 | None |
| M291T827 | 291.2317 | 13.79 | C19H30O2 | Stearidonic acid methyl ester | [M+H]+ | 0.8232 | None |
| M292T198 | 292.1181 | 3.29 | C19H27NO7 | Bruceolline F | [M+H-C4H10O2]+ | 0.8177 | None |
| M293T818 | 293.211 | 13.63 | C18H32O5 | (10E,15Z)-9,12,13-Trihydroxyoctadeca-10,15-dienoic acid | [M+H-2H2O]+ | 0.8184 | None |
| M294T275 | 294.1443 | 4.58 | C16H20FNO3 | 3-[(4-Fluoroanilino)carbonyl]-1,2,2-trimethylcyclopentanecarboxylic acid | [M+H]+ | 0.8458 | None |
| M294T160 | 294.1547 | 2.67 | C12H23NO7 | N-fructosyl isoleucine | [M+H]+ | 0.9372 | None |
| M295T202 | 295.1289 | 3.37 | C14H18N2O5 | Phe-Glu | [M+H]+ | 0.9935 | None |
| M295T250 | 295.1289 | 4.16 | C14H18N2O5 | Glu-Phe | [M+H]+ | 0.9031 | None |
| M295T267 | 295.1285 | 4.44 | C14H18N2O5 | gamma-Glutamylphenylalanine | [M+H]+ | 0.9773 | None |
| M295T892 | 295.1329 | 14.87 | C19H18O3 | Tanshinone IIA | [M+H]+ | 0.9927 | None |
| M295T746_2 | 295.2264 | 12.43 | C19H34O4 | 9,12-Octadecadienoic acid, 15-hydroxy-16-methoxy- | [M+H-CH4O]+ | 0.7572 | None |
| M295T849 | 295.2267 | 14.15 | C18H30O3 | 9-Oxo-ODE | [M+H]+ | 0.8407 | None |
| M297T391 | 297.0394 | 6.51 | C16H11ClN2O3 | 5-[(7-Chloro-4-quinolinyl)amino]-2-hydroxybenzoic acid | [M+H-H2O]+ | 0.9297 | None |
| M297T829 | 297.0583 | 13.82 | C10H20N2S4 | Disulfiram | [M+H]+ | 0.986 | None |
| M297T185 | 297.1081 | 3.08 | C13H16N2O6 | Asp-Tyr | [M+H]+ | 0.8795 | None |
| M297T756 | 297.2418 | 12.59 | C18H34O4 | 12,13-DiHOME | [M+H-H2O]+ | 0.9146 | None |
| M297T436 | 297.2575 | 7.26 | C28H46O | Brassicasterol | [M+H-C6H14O]+ | 0.9638 | None |
| M298T234 | 298.0969 | 3.9 | C11H15N5O3S | 5'-S-Methyl-5'-thioadenosine | [M+H]+ | 0.9987 | None |
| M299T448 | 299.0537 | 7.47 | C16H10O6 | 3,8-Dihydroxy-1-methylanthraquinone-2-carboxylic acid | [M+H]+ | 0.9701 | None |
| M299T413 | 299.2367 | 6.89 | C21H34O3 | Heneicosapentaenoic acid | [M+H-H2O]+ | 0.9638 | None |
| M299T436 | 299.2367 | 7.26 | C21H32O2 | 17.alpha.-Hydroxypregnanolone | [M+H-2H2O]+ | 0.9623 | None |
| M299T608 | 299.2368 | 10.14 | C21H34O3 | 21-Hydroxypregnanolone | [M+H-2H2O]+ | 0.9626 | None |
| M300T814 | 300.1957 | 13.56 | C19H25NO2 | Nylidrin | [M+H]+ | 0.9589 | None |
| M301T609 | 301.0705 | 10.15 | C16H12O6 | Chrysoeriol | [M+H]+ | 0.9291 | None |
| M301T386 | 301.0706 | 6.43 | C16H13ClN2O2 | Temazepam | [M+H]+ | 0.963 | Into_Blood |
| M301T872 | 301.141 | 14.53 | C18H20O4 | 2,3',4,5'-Tetramethoxystilbene | [M+H]+ | 0.9937 | None |
| M301T816 | 301.2162 | 13.6 | C20H30O3 | 17(18)-EpETE | [M+H-H2O]+ | 0.9842 | None |
| M301T436 | 301.2524 | 7.26 | C23H38FNO | (.+/-.)-2-Methylarachidonoyl-2'-fluoroethylamide | [M+H-C2H6FN]+ | 0.9217 | None |
| M302T66 | 302.1347 | 1.11 | C17H19NO4 | [3-[(E)-2-(1,3-Benzodioxol-5-yl)vinyl]oxiran-2-yl]-piperidinomethanone | [M+H]+ | 0.9782 | None |
| M302T301 | 302.1498 | 5.01 | C16H19N3O3 | Trp-Pro | [M+H]+ | 0.9004 | None |
| M302T237 | 302.2074 | 3.94 | C14H27N3O4 | Leu-Gly-Leu | [M+H]+ | 0.7852 | None |
| M302T849_2 | 302.2112 | 14.15 | C22H33NO4 | Tuberostemonin | [M+H-C3H6O2]+ | 0.9614 | None |
| M302T713_1 | 302.3053 | 11.88 | C18H39NO2 | Sphinganine (d18:0) | [M+H]+ | 0.9792 | None |
| M303T350 | 303.0135 | 5.83 | C14H6O8 | Ellagic acid | [M+H]+ | 0.9712 | None |
| M303T316_1 | 303.0498 | 5.26 | C15H10O7 | Morin | [M+H]+ | 0.9991 | None |
| M303T300 | 303.0499 | 5 | C21H20O12 | Isoquercitin | [M+H-C6H10O5]+ | 0.9817 | None |
| M303T411_2 | 303.0861 | 6.86 | C16H14O6 | Hesperetin | [M+H]+ | 0.9636 | None |
| M303T186 | 303.1051 | 3.1 | C20H14O3 | 2'-Methoxy-7,8-benzoflavone | [M+H]+ | 0.9733 | Into_Blood |
| M303T796 | 303.1954 | 13.26 | C19H26O3 | 7.alpha.-Hydroxyandrostenedione | [M+H]+ | 0.8208 | None |
| M303T650 | 303.232 | 10.84 | C20H34O4 | 18,19-Dihydroxy-3-cleroden-15-oic acid | [M+H-2H2O]+ | 0.8893 | None |
| M304T65 | 304.1615 | 1.09 | C11H21N5O5 | Glu-Arg | [M+H]+ | 0.9107 | None |
| M304T863 | 304.2271 | 14.39 | C19H29NO2 | Iromycin A | [M+H]+ | 0.9675 | None |
| M304T746 | 304.2998 | 12.43 | C21H38N | Pyridinium, 1-hexadecyl- | [Cat]+ | 0.7074 | None |
| M305T127 | 305.1343 | 2.12 | C15H22O5 | Lactarolide A | [M+Na]+ | 0.928 | None |
| M305T947_2 | 305.2464 | 15.78 | C20H34O3 | 14(15)-EpEDE | [M+H-H2O]+ | 0.916 | None |
| M307T417 | 307.0599 | 6.94 | C16H9F3O3 | 7-Hydroxy-2-(trifluoromethyl)isoflavone | [M+H]+ | 0.9914 | Into_Blood |
| M307T218 | 307.0814 | 3.64 | C15H14O7 | (-)-Gallocatechin | [M+H]+ | 0.9929 | None |
| M308T503 | 308.222 | 8.38 | C18H29NO3 | Gabapentin related compound D | [M+H]+ | 0.9668 | None |
| M309T281 | 309.0968 | 4.68 | C21H22O9 | Robustaside A | [M+H-C6H6O2]+ | 0.9689 | None |
| M309T292 | 309.0968 | 4.87 | C22H28O11 | 2-(3-Hydroxy-5-oxotetrahydrofuran-3-yl)propyl 6-O-((2E)-3-(4-hydroxyphenyl)prop-2-enoyl)-.beta.-D-glucopyranoside | [M+H-C7H12O4]+ | 0.9872 | None |
| M309T433 | 309.1177 | 7.22 | C12H22O10 | Rutinose | [M+H-H2O]+ | 0.8538 | None |
| M309T55 | 309.1656 | 0.91 | C12H24N2O7 | Fructoselysine | [M+H]+ | 0.785 | None |
| M310T1031_1 | 310.3104 | 17.18 | C20H39NO | Oleoyl ethylamide | [M+H]+ | 0.9235 | None |
| M311T658 | 311.2215 | 10.97 | C18H30O4 | trans-EKODE-(E)-Ib | [M+H]+ | 0.784 | None |
| M311T936 | 311.2557 | 15.61 | C19H36O4 | Monopalmitolein (9c) | [M+H-H2O]+ | 0.9852 | None |
| M312T375 | 312.1227 | 6.25 | C18H19NO5 | N-trans-Feruloyloctopamine | [M+H-H2O]+ | 0.9782 | None |
| M312T207 | 312.1298 | 3.45 | C12H17N5O5 | N,N-Dimethylguanosine | [M+H]+ | 0.9997 | None |
| M313T689 | 313.0705 | 11.48 | C17H14O7 | 3-(1,2-Dihydroxypropyl)-1,6,8-trihydroxy-9,10-anthraquinone | [M+H-H2O]+ | 0.9532 | None |
| M313T344 | 313.1547 | 5.74 | C18H20N2O3 | Phe-Phe | [M+H]+ | 0.9937 | None |
| M313T678 | 313.2373 | 11.31 | C18H32O4 | (9Z,12E)-15,16-Dihydroxyoctadeca-9,12-dienoic acid | [M+H]+ | 0.7921 | None |
| M314T479_2 | 314.1385 | 7.99 | C18H19NO4 | Moupinamide | [M+H]+ | 0.9502 | None |
| M315T391 | 315.0498 | 6.52 | C25H26O7 | 2-[3-(3,7-Dimethylocta-2,6-dienyl)-2,6-dihydroxybenzoyl]-5-formyl-3-hydroxybenzoic acid | [M+H-C9H16]+ | 0.9729 | None |
| M315T351 | 315.071 | 5.85 | C28H24O16 | 2''-Galloylhyperin | [M+H-C15H10O7]+ | 0.9888 | None |
| M315T707 | 315.0862 | 11.78 | C17H14O6 | Gnaphaliin | [M+H]+ | 0.8711 | None |
| M317T349 | 317.0655 | 5.81 | C16H12O7 | Isorhamnetol | [M+H]+ | 0.9986 | None |
| M318T326 | 318.1813 | 5.43 | C17H23N3O3 | Ile-Trp | [M+H]+ | 0.9242 | None |
| M318T824 | 318.2405 | 13.73 | C20H31NO2 | Drofenine | [M+H]+ | 0.9981 | None |
| M318T717 | 318.3003 | 11.95 | C18H39NO3 | Phytosphingosine | [M+H]+ | 0.9322 | None |
| M319T363 | 319.0813 | 6.06 | C16H14O7 | Lecanoric acid | [M+H]+ | 0.8301 | Into_Blood |
| M320T426 | 320.0915 | 7.11 | C19H14NO4+ | Coptisine | [M]+ | 0.9982 | Into_Blood |
| M321T436 | 321.2576 | 7.26 | C24H38O3 | 5-Cholenic acid-3.beta.-ol | [M+H-3H2O]+ | 0.9752 | None |
| M321T1003 | 321.3152 | 16.71 | C22H42O2 | Erucic acid | [M+H-H2O]+ | 0.9486 | None |
| M323T486 | 323.1466 | 8.1 | C18H21N5O2 | Alogliptin | [M+H-NH3]+ | 0.997 | None |
| M324T128 | 324.0592 | 2.13 | C9H14N3O8P | 3'-CMP | [M+H]+ | 0.9993 | Into_Blood |
| M324T889 | 324.2898 | 14.81 | C20H37NO2 | Linoleoyl ethanolamide | [M+H]+ | 0.9808 | None |
| M325T577_1 | 325.1128 | 9.61 | C12H22O11 | Glycan MC=h2 | [M+H-H2O]+ | 0.9809 | Into_Blood |
| M326T237 | 326.1586 | 3.95 | C16H23NO6 | Crotaline | [M+H]+ | 0.9105 | None |
| M327T823 | 327.0781 | 13.71 | C18H15O4P | Triphenyl phosphate | [M+H]+ | 0.9663 | None |
| M327T323 | 327.1592 | 5.38 | C20H26O6 | (-)-Secoisolariciresinol | [M+H-2H2O]+ | 0.9712 | None |
| M327T271 | 327.2015 | 4.52 | C20H29N3O2 | ADBICA | [M+H-NH3]+ | 0.8052 | None |
| M327T899_2 | 327.2297 | 14.98 | C22H32O3 | 7-HDoHE | [M+H-H2O]+ | 0.9829 | None |
| M327T849 | 327.234 | 14.14 | C22H32O3 | 4-HDoHE | [M+H-H2O]+ | 0.9921 | None |
| M328T198 | 328.1392 | 3.29 | C15H21NO7 | N-Fructosyl phenylalanine | [M+H]+ | 0.8105 | None |
| M329T735 | 329.1747 | 12.25 | C20H24O4 | Cembratetraene-16,2:19,6-diolide | [M+H]+ | 0.7433 | None |
| M328T731 | 328.2482 | 12.19 | C18H30O4 | Sterebin A | [M+NH4]+ | 0.7192 | None |
| M329T359 | 329.2183 | 5.98 | C21H30O4 | Corticosterone | [M+H-H2O]+ | 0.903 | None |
| M330T728 | 330.3003 | 12.14 | C19H39NO3 | Palmitoylserinol | [M+H]+ | 0.9296 | None |
| M331T462 | 331.154 | 7.7 | C19H24O6 | MEGxp0_001644 | [M-H2O+H]+ | 0.8328 | None |
| M331T681 | 331.1881 | 11.35 | C19H26FN3O2 | 5-Fluoro ABICA | [M+H-NH3]+ | 0.9978 | None |
| M333T732 | 333.2035 | 12.19 | C20H30O5 | Andrographolide | [M+H-H2O]+ | 0.999 | None |
| M334T290 | 334.1398 | 4.84 | C16H19N3O5 | Glu-Trp | [M+H]+ | 0.8732 | None |
| M336T483_2 | 336.1225 | 8.04 | C20H18NO4+ | Majarine | [M+]+ | 0.9989 | Into_Blood |
| M336T420 | 336.1229 | 7.01 | C20H18NO4+ | Epiberberine | [M]+ | 0.9967 | None |
| M336T911 | 336.3261 | 15.19 | C22H41NO | Pipericine | [M+H]+ | 0.7528 | None |
| M337T840 | 337.2737 | 14 | C21H38O4 | 2-Linoleoylglycerol | [M+H-H2O]+ | 0.9512 | None |
| M338T415 | 338.1386 | 6.92 | C20H20NO4+ | Neprotin | [M]+ | 0.9869 | Into_Blood |
| M339T265 | 339.1076 | 4.41 | C22H30O14 | 4-O-p-Coumaroylquinic acid | [M+H]+ | 0.9984 | None |
| M339T406 | 339.2682 | 6.76 | C24H40O4 | 3-Ketopetromyzonol | [M+H-3H2O]+ | 0.9458 | None |
| M339T1040 | 339.2872 | 17.34 | C21H40O4 | Monoelaidin | [M+H-H2O]+ | 0.9919 | None |
| M339T1024 | 339.2873 | 17.07 | C37H70O5 | 1-Oleoyl-2-palmitoyl-rac-glycerol | [M+H-C16H32O2]+ | 0.9674 | None |
| M341T538 | 341.1383 | 8.97 | C20H22O6 | Pinoresinol | [M+H-H2O]+ | 0.9092 | Into_Blood |
| M341T463 | 341.1384 | 7.72 | C26H32O11 | 4-((2S,3S)-3-(Hydroxymethyl)-5-((1E)-3-hydroxyprop-1-en-1-yl)-7-methoxy-2,3-dihydro-1-benzofuran-2-yl)-2-methoxyphenyl hexopyranoside | [M+H-C6H12O6]+ | 0.9445 | None |
| M341T426 | 341.1571 | 7.1 | C12H24N2O9 | Chitobiose | [M+H]+ | 0.9988 | Into_Blood |
| M341T413 | 341.2837 | 6.89 | C24H40O4 | 3-Ketopetromyzonol | [M+H-3H2O]+ | 0.9326 | None |
| M343T672 | 343.2957 | 11.19 | [C19H39N2O3]+ | Cocamidoprpylbetaine | [M]+ | 0.9959 | None |
| M344T150 | 344.1341 | 2.5 | C15H21NO8 | N-Fructosyl tyrosine | [M+H]+ | 0.8385 | None |
| M344T494 | 344.1492 | 8.23 | C19H21NO5 | Feruloyl o-methyldopamine | [M+H]+ | 0.9605 | Into_Blood |
| M347T240 | 347.1316 | 4 | C21H18N2O3 | MS-1020 | [M+H]+ | 0.971 | None |
| M348T915 | 348.2874 | 15.24 | C22H37NO2 | Anandamide | [M+H]+ | 0.9859 | None |
| M351T688 | 351.2319 | 11.47 | C24H34O4 | Bufalin | [M+H-2H2O]+ | 0.9536 | Into_Blood |
| M352T475 | 352.1543 | 7.92 | C21H22NO4+.Cl- | Palmatine | [M]+ | 0.9964 | None |
| M352T362 | 352.1656 | 6.03 | C20H21N3O3 | Phe-Trp | [M+H]+ | 0.9696 | None |
| M353T674 | 353.2476 | 11.23 | C12H16O | 2-Tetralin-1-ylethanol | [2M+H]+ | 0.8709 | None |
| M355T267_2 | 355.1025 | 4.45 | C16H18O9 | Chlorogenate | [M+H]+ | 0.9986 | Into_Blood |
| M355T658 | 355.2632 | 10.96 | C24H40O5 | Ursocholic acid | [M+H-3H2O]+ | 0.9891 | None |
| M355T956 | 355.2818 | 15.93 | C21H38O4 | 1-Monolinolein | [M+H]+ | 0.997 | None |
| M360T576 | 360.1498 | 9.6 | C12H22O11 | D-(+)-Trehalose | [M+NH4]+ | 0.9639 | Into_Blood |
| M360T552 | 360.1499 | 9.21 | C12H22O11 | Gentiobiose | [M+NH4]+ | 0.9562 | None |
| M360T618 | 360.1499 | 10.29 | C12H22O11 | .alpha.,.beta.-Trehalose | [M+NH4]+ | 0.9666 | None |
| M360T82 | 360.15 | 1.36 | C12H22O11 | Leucrose | [M+NH4]+ | 0.9571 | None |
| M361T300 | 361.0894 | 5 | C18H16O8 | Irigenin | [M+H]+ | 0.9948 | None |
| M362T259 | 362.181 | 4.32 | C16H24O8 | Dihydroconiferin | [M+NH4]+ | 0.889 | None |
| M363T340 | 363.105 | 5.66 | C16H20O8 | Linocinnamarin | [M+Na]+ | 0.9957 | None |
| M365T745 | 365.1053 | 12.41 | C12H22O11 | Sucrose | [M+Na]+ | 0.9474 | None |
| M365T1008 | 365.1056 | 16.8 | C12H22O11 | Melibiose | [M+Na]+ | 0.9988 | None |
| M366T494 | 366.1311 | 8.23 | C44H71NO13 | (3.beta.,5.Xi.,9.Xi.)-3-((2-Acetamido-2-deoxy-6-O-.beta.-D-glucopyranosyl-.beta.-D-glucopyranosyl)oxy)olean-12-en-28-oic acid | [M+H-C30H48O3]+ | 0.9906 | None |
| M367T710 | 367.2455 | 11.83 | C19H36O5 | 1,2-Dioctanoyl-sn-glycerol | [M+Na]+ | 0.9968 | None |
| M369T316_2 | 369.1179 | 5.26 | C17H20O9 | 3-O-Feruloylquinic acid | [M+H]+ | 0.9918 | None |
| M369T577_2 | 369.3151 | 9.61 | C27H42O3 | 3-Oxo-4-cholestenoic acid | [M+H-CH2O2]+ | 0.8197 | None |
| M371T284_1 | 371.2277 | 4.74 | C22H30N2O3 | Methyl (1-(cyclohexylmethyl)-1H-indole-3-carbonyl)-L-valinate | [M+H]+ | 0.8053 | None |
| M373T752 | 373.1986 | 12.53 | C16H28N4O4S | Biocytin | [M+H]+ | 0.9989 | None |
| M373T676 | 373.2739 | 11.26 | C24H40O5 | .beta.-Muricholic acid | [M+H-2H2O]+ | 0.9343 | None |
| M374T926 | 374.303 | 15.44 | C24H39NO2 | 7(Z),10(Z),13(Z),16(Z),19(Z)-N-(2-Hydroxyethyl)docosapentaenamide | [M+H]+ | 0.9965 | None |
| M375T705 | 375.1073 | 11.75 | C19H18O8 | Neobaicalein | [M+H]+ | 0.966 | None |
| M376T973 | 376.3186 | 16.21 | C24H41NO2 | Docosatetraenoyl ethanolamide | [M+H]+ | 0.999 | None |
| M377T267 | 377.0845 | 4.45 | C16H18O9 | Scopolin | [M+Na]+ | 0.9347 | None |
| M377T203 | 377.142 | 3.38 | C15H22N4O6 | Glp-Glu-Pro-NH2 | [M+Na]+ | 0.9883 | None |
| M377T288 | 377.1457 | 4.8 | C17H20N4O6 | Vitamin B2 | [M+H]+ | 0.9726 | None |
| M378T202 | 378.176 | 3.37 | C16H24O9 | Junipediol A 8-glucoside | [M+NH4]+ | 0.9779 | None |
| M381T684_2 | 381.3152 | 11.4 | C27H44O3 | 1,24-Dihydroxyvitamin D3 | [M+H-2H2O]+ | 0.876 | None |
| M382T210 | 382.1714 | 3.5 | C16H23N5O6 | Zeatin-9-glucoside | [M+H]+ | 0.8667 | None |
| M383T892 | 383.1829 | 14.87 | C22H26N2O2S | Eletriptan | [M+H]+ | 0.9938 | None |
| M386T317 | 386.1243 | 5.28 | C20H17F2N3O3 | Sarafin | [M+H]+ | 0.9966 | None |
| M387T241 | 387.2714 | 4.01 | C17H34N6O4 | Val-Leu-Arg | [M+H]+ | 0.8666 | None |
| M388T830 | 388.1058 | 13.83 | C19H18ClN3O4 | Pyraclostrobin | [M+H]+ | 0.9065 | None |
| M388T715 | 388.1308 | 11.91 | C21H22ClNO4 | Dimethomorph | [M+H]+ | 0.8671 | None |
| M391T316 | 391.1002 | 5.26 | C17H20O9 | 1,3,5-Trihydroxy-4-[(E)-3-(3-hydroxy-4-methoxyphenyl)acryloyl]oxycyclohexanecarboxylic acid | [M+Na]+ | 0.9956 | None |
| M393T601 | 393.3513 | 10.01 | C29H46O | (3.beta.,5.alpha.)-4,4-Dimethylcholesta-8,14,24-trien-3-ol | [M+H-H2O]+ | 0.8965 | None |
| M397T251 | 397.1131 | 4.18 | C16H22O10 | Geniposidic acid | [M+Na]+ | 0.7374 | Into_Blood |
| M397T788 | 397.3101 | 13.14 | C27H42O3 | Nitogenin | [M+H-H2O]+ | 0.9311 | Into_Blood |
| M398T746 | 398.2414 | 12.43 | C24H31NO4 | 2-[4-(Diamylamino)-2-hydroxybenzoyl]benzoic acid | [M+H]+ | 0.9979 | None |
| M399T855_1 | 399.2506 | 14.25 | C18H39O7P | Tris(2-butoxyethyl) phosphate | [M+H]+ | 0.9597 | None |
| M401T516 | 401.1594 | 8.6 | C22H26O8 | Syringaresinol | [M+H-H2O]+ | 0.8414 | None |
| M403T464_1 | 403.0926 | 7.73 | C21H19ClO6 | (5-Benzoyloxy-3-chloro-4,6-dihydroxycyclohexen-1-yl)methyl benzoate | [M+H]+ | 0.9415 | None |
| M403T274_1 | 403.1577 | 4.57 | C25H31ClO6 | Ilicicolin F | [M+H-C2H4O2]+ | 0.9913 | None |
| M403T597 | 403.2156 | 9.95 | C16H30N6O4S | Pro-Met-Arg | [M+H]+ | 0.9428 | None |
| M404T964 | 404.3135 | 16.07 | C25H41NO3 | N-(14-Methylpentadecanoyl)phenylalanine | [M+H]+ | 0.9203 | None |
| M409T287 | 409.1832 | 4.78 | C20H28N2O5S | (-)-Tamsulosin | [M+H]+ | 0.9976 | None |
| M409T242 | 409.187 | 4.04 | C11H12N2O2 | Tryptophan | [2M+H]+ | 0.8428 | None |
| M409T453 | 409.3464 | 7.54 | C30H48O4 | Hederagenol | [M+H-CH4O3]+ | 0.9234 | None |
| M409T557 | 409.3828 | 9.29 | C32H52O2 | Handianol, acetate | [M+H-C2H4O2]+ | 0.8893 | None |
| M411T601 | 411.362 | 10.01 | C31H50O3 | Methyl ursolate | [M+H-C2H4O2]+ | 0.9489 | None |
| M412T409 | 412.2229 | 6.82 | C21H33NO7 | Lasiocarpine | [M+H]+ | 0.8589 | Into_Blood |
| M413T245 | 413.1416 | 4.09 | C15H20N6O8 | N6-threonylcarbamoyladenosine | [M+H]+ | 0.7701 | None |
| M413T524 | 413.1782 | 8.74 | C25H26O4 | Erysubin F | [M+Na]+ | 0.9918 | None |
| M415T373_1 | 415.1363 | 6.21 | C20H24O8 | Vernodalol | [M+Na]+ | 0.9222 | None |
| M415T755_2 | 415.2115 | 12.59 | C24H32O7 | Gelomulide N | [M-H2O+H]+ | 0.9844 | None |
| M416T463 | 416.3522 | 7.72 | C27H45NO2 | Tomatidine | [M+H]+ | 0.9966 | None |
| M417T708 | 417.1883 | 11.81 | C23H28O7 | Magnolin | [M+H]+ | 0.9882 | None |
| M419T293 | 419.1334 | 4.88 | C21H22O9 | Neoliquiritin | [M+H]+ | 0.9897 | Into_Blood |
| M419T363_1 | 419.1335 | 6.04 | C21H22O9 | Gaylussacin | [M+H]+ | 0.9985 | None |
| M419T331 | 419.3308 | 5.52 | C32H50O5 | Alisol B acetate | [M+H-C2H8O4]+ | 0.9295 | None |
| M420T285 | 420.1864 | 4.76 | C18H26O10 | Benzyl alcohol + hex-pen | [M+NH4]+ | 0.7602 | None |
| M420T599 | 420.2078 | 9.98 | C19H31N3O6 | CA-074, methyl ester | [M+Na]+ | 0.9522 | None |
| M421T207 | 421.1343 | 3.45 | C17H24O12 | CHEMBL2335272 | [M+H]+ | 0.8874 | None |
| M421T323 | 421.1469 | 5.39 | C21H24O9 | Rhapontin | [M+H]+ | 0.9544 | None |
| M421T419 | 421.3462 | 6.98 | C30H50O4 | (23E)-16,20,25-Trihydroxylanost-23-en-3-one | [M+H-3H2O]+ | 0.9053 | Into_Blood |
| M423T341 | 423.3255 | 5.68 | C29H46O4 | Platanic acid | [M+H-2H2O]+ | 0.7847 | None |
| M423T436_3 | 423.3617 | 7.26 | C36H62O9 | Ginsenoside F1 | [M+H-C6H16O8]+ | 0.9693 | Into_Blood |
| M425T287 | 425.142 | 4.79 | C18H26O10 | 4-(.beta.-D-Glucopyranosyloxy)benzyl 2,3-dihydroxy-3-methylbutanoate | [M+Na]+ | 0.998 | None |
| M425T894 | 425.2897 | 14.9 | C26H36N2O3 | Arachidonoyl p-nitroaniline | [M+H]+ | 0.7232 | None |
| M425T577 | 425.3775 | 9.61 | C30H48O | 17(21)-Hopen-6-one | [M+H]+ | 0.93 | Into_Blood |
| M431T392 | 431.0972 | 6.53 | C21H20O11 | 8-C-Galactosylluteolin | [M+H-H2O-CH4O2]+ | 0.8709 | None |
| M432T755 | 432.2381 | 12.59 | C22H30O6 | 7b,9-Dihydroxy-3-(hydroxymethyl)-1,1,6,8-tetramethyl-5-oxo-1,1a,1b,4,4a,5,7a,7b,8,9-decahydro-9aH-cyclopropa[3,4]benzo[1,2-e]azulen-9a-yl acetate | [M+ACN+H]+ | 0.9469 | None |
| M433T250 | 433.1131 | 4.17 | C21H20O10 | Sophoricoside | [M+H]+ | 0.9074 | Into_Blood |
| M434T323 | 434.2021 | 5.38 | C19H28O10 | 2-Phenylethyl 2-O-.beta.-D-xylopyranosyl-.beta.-D-glucopyranoside | [M+NH4]+ | 0.9673 | None |
| M435T399 | 435.1285 | 6.66 | C21H22O10 | Naringenin 7-O-beta-D-glucoside | [M+H]+ | 0.9969 | None |
| M437T962_1 | 437.1935 | 16.04 | C24H30O6 | MCULE-9532757463 | [M+Na]+ | 0.9916 | None |
| M437T352 | 437.3415 | 5.86 | C42H68O13 | Saikosaponin D | [M+H-C12H24O11]+ | 0.9332 | None |
| M439T524 | 439.0948 | 8.74 | C21H20O9 | Chrysophanol 8-O-beta-D-glucoside | [M+Na]+ | 0.9984 | None |
| M439T283_2 | 439.2163 | 4.71 | C25H30N2O5 | Quinapril | [M+H]+ | 0.8961 | None |
| M439T601_2 | 439.3568 | 10.01 | C30H48O3 | Ursolic acid | [M+H-H2O]+ | 0.9155 | Into_Blood |
| M439T419 | 439.357 | 6.98 | C52H86O22 | Anagallisin C | [M+H-C22H40O20]+ | 0.8244 | Into_Blood |
| M439T383 | 439.357 | 6.39 | C32H50O4 | Acetyl lupeolic acid | [M+H-C2H4O2]+ | 0.845 | None |
| M441T436_2 | 441.3725 | 7.26 | C42H70O12 | (3.beta.,6.alpha.,12.beta.,20Z)-3,12-Dihydroxydammara-20(22),24-dien-6-yl 2-O-(6-deoxy-.alpha.-L-mannopyranosyl)-.beta.-D-glucopyranoside | [M+H-C12H22O10]+ | 0.9622 | Into_Blood |
| M441T908 | 441.3728 | 15.14 | C30H48O2 | 22-Hydoxy-2-hopen-1-one | [M+H]+ | 0.9106 | Into_Blood |
| M442T747 | 442.3531 | 12.45 | C25H47NO5 | 3-Hydroxyoleylcarnitine | [M+H]+ | 0.9729 | None |
| M443T354 | 443.0974 | 5.91 | C22H18O10 | Epicatechin gallate | [M+H]+ | 0.9777 | None |
| M443T592 | 443.3882 | 9.86 | C30H50O2 | 22-hydroxy-6-hopanone | [M+H]+ | 0.9461 | Into_Blood |
| M445T473 | 445.1129 | 7.88 | C22H22O11 | .beta.-D-Glucopyranose, 6-[(2E)-3-phenyl-2-propenoate] 1-(3,4,5-trihydroxybenzoate) | [M+H-H2O]+ | 0.989 | None |
| M445T783 | 445.2198 | 13.05 | C27H28N2O4 | Asperglaucide | [M+H]+ | 0.991 | None |
| M447T445 | 447.0922 | 7.42 | C21H18O11 | Baicalin | [M+H]+ | 0.9994 | Into_Blood |
| M447T418 | 447.22 | 6.97 | C19H36O10 | Rhodiooctanoside | [M+Na]+ | 0.9986 | None |
| M449T394 | 449.1078 | 6.57 | C21H20O11 | 3,5-dihydroxy-2-(4-hydroxyphenyl)-7-{[3,4,5-trihydroxy-6-(hydroxymethyl)oxan-2-yl]oxy}-4H-chromen-4-one | [M+H]+ | 0.9993 | None |
| M449T343 | 449.108 | 5.72 | C21H20O11 | Trifolin | [M+H]+ | 0.9861 | Into_Blood |
| M453T250 | 453.1393 | 4.17 | C21H24O11 | 2-(3,4-Dihydroxyphenyl)-5,7-dihydroxy-3,4-dihydro-2H-chromen-3-yl .beta.-D-glucopyranoside | [M+H]+ | 0.9839 | None |
| M453T755 | 453.1675 | 12.59 | C22H28O10 | 5-O-Methylvisammioside | [M+H]+ | 0.998 | None |
| M453T159 | 453.2093 | 2.65 | C19H28N6O7 | Asp-Tyr-Arg | [M+H]+ | 0.8523 | None |
| M453T686_2 | 453.3361 | 11.43 | C30H46O4 | 11-Oxo-.beta.-boswellic acid | [M+H-H2O]+ | 0.8786 | None |
| M453T328 | 453.3437 | 5.46 | C35H56O9 | (24E)-12,15-Dihydroxy-3-(pentopyranosyloxy)-9,19-cyclolanost-24-en-26-oic acid | [M+H-C5H12O6]+ | 0.7699 | None |
| M455T509 | 455.0948 | 8.49 | C21H20O10 | Emodin 8-glucoside | [M+Na]+ | 0.9818 | None |
| M455T426 | 455.0949 | 7.1 | C21H20O10 | Cosmosiin | [M+Na]+ | 0.9907 | None |
| M455T352 | 455.3521 | 5.87 | C30H52O6 | 6-(2-(3-Hydroxy-2,2,5a-trimethyl-7-methylidenedecahydro-1-benzoxepin-6-yl)ethyl)-2,2,5a,7-tetramethyldecahydro-1-benzoxepine-3,4,6-triol | [M+H-3H2O]+ | 0.8834 | None |
| M457T996 | 457.2327 | 16.6 | C25H32N2O6 | Vindolin | [M+H]+ | 0.9829 | None |
| M457T271 | 457.3677 | 4.52 | C30H48O3 | 3-Hydroxylanosta-7,24-dien-21-oic acid | [M+H]+ | 0.7249 | None |
| M461T340 | 461.1079 | 5.66 | C22H20O11 | Oroxindin | [M+H]+ | 0.9949 | None |
| M461T901 | 461.2875 | 15.02 | C23H36N6O4 | Lys-Trp-Lys | [M+H]+ | 0.9974 | Into_Blood |
| M463T348 | 463.1235 | 5.8 | C22H22O11 | Tectoridin | [M+H]+ | 0.9911 | None |
| M463T270 | 463.2152 | 4.49 | C19H36O11 | 3-Ethyl-4-hydroxy-4-methylpentyl 6-O-((2S,3R,4R)-3,4-dihydroxy-4-(hydroxymethyl)tetrahydrofuran-2-yl)-.beta.-D-glucopyranoside | [M+Na]+ | 0.9923 | None |
| M465T316 | 465.1029 | 5.26 | C21H20O12 | Hyperin | [M+H]+ | 0.9976 | None |
| M469T348 | 469.0746 | 5.8 | C19H18N4O8 | Bispyribac | [M+K]+ | 0.9163 | Into_Blood |
| M471T503 | 471.2202 | 8.39 | C21H36O10 | 2-(4-Methylcyclohex-3-en-1-yl)propan-2-yl 6-O-pentofuranosylhexopyranoside | [M+Na]+ | 0.9985 | None |
| M473T628 | 473.142 | 10.47 | C22H26O10 | 4-Methoxyphlorizin | [M+Na]+ | 0.9897 | None |
| M473T905 | 473.3627 | 15.09 | C30H48O4 | Echinocystic acid | [M+H]+ | 0.8132 | None |
| M477T301 | 477.3939 | 5.02 | C30H52O4 | Panaxatriol | [M+H]+ | 0.966 | None |
| M478T787_2 | 478.2928 | 13.12 | C20H36N4O8 | Desferrioxamine H | [M+NH4]+ | 0.8485 | None |
| M479T349 | 479.1186 | 5.82 | C22H22O12 | Isorhamnetin 3-O-glucoside | [M+H]+ | 0.9885 | None |
| M479T983 | 479.2147 | 16.38 | C24H32N4O5 | TAPI-0 | [M+Na]+ | 0.9384 | None |
| M481T402 | 481.168 | 6.69 | C21H30O11 | 4-(Prop-2-en-1-yl)phenyl 6-O-hexopyranosylhexopyranoside | [M+Na]+ | 0.9977 | None |
| M483T307 | 483.1843 | 5.12 | C21H32O11 | Apiosylrhododendrin | [M+Na]+ | 0.9944 | None |
| M484T270 | 484.3244 | 4.51 | C24H47NO7 | Psychosine | [M+Na]+ | 0.8324 | None |
| M484T880_2 | 484.4725 | 14.66 | C30H61NO3 | C12 Dihydroceramide (d18:0/12:0) | [M+H]+ | 0.8247 | None |
| M487T577 | 487.1657 | 9.61 | C18H32O16 | Maltotriose | [M+H-H2O]+ | 0.9741 | Into_Blood |
| M487T431 | 487.3489 | 7.18 | C30H48O6 | Terminolic acid | [M+H-H2O]+ | 0.893 | None |
| M495T365 | 495.1473 | 6.08 | C23H28O13 | Picroside II | [M+H-H2O]+ | 0.9307 | None |
| M495T381 | 495.1473 | 6.36 | C21H28O12 | 4-O-((2E)-3-Phenylprop-2-enoyl)-.beta.-D-fructofuranosyl .alpha.-D-glucopyranoside | [M+Na]+ | 0.9664 | None |
| M495T905 | 495.3445 | 15.09 | C30H48O4 | Glucosol | [M+Na]+ | 0.9925 | None |
| M496T904 | 496.3402 | 15.07 | C24H50NO7P | Lyso-PC(16:0) | [M+H]+ | 0.9382 | None |
| M499T986 | 499.3759 | 16.43 | C32H50O4 | 3-(Acetyloxy)lanosta-8,24-dien-21-oic acid | [M+H]+ | 0.9846 | Into_Blood |
| M501T392 | 501.1004 | 6.53 | C22H22O12 | Nepetin 7-glucoside | [M+Na]+ | 0.995 | None |
| M513T902 | 512.5039 | 15.04 | C32H65NO3 | C14 Dihydroceramide | [M+H]+ | 0.8688 | None |
| M513T292 | 513.1944 | 4.87 | C26H28N2O9 | Lyalosidic acid | [M+H]+ | 0.9982 | None |
| M514T524_1 | 514.2797 | 8.74 | C26H43NO7S | 2-((4R)-4-((3R,5R,6S,7R,9S,10R,13R,14S,17R)-3,6,7-trihydroxy-10,13-dimethylhexadecahydro-1H-cyclopenta[a]phenanthren-17-yl)pent-2-enamido)ethane-1-sulfonic acid | [M+H]+ | 0.7602 | None |
| M521T739 | 521.3625 | 12.32 | C32H50O4 | 3-(Acetyloxy)urs-12-en-23-oic acid | [M+Na]+ | 0.7788 | None |
| M525T296 | 525.1578 | 4.93 | C24H28O13 | Verminoside | [M+H]+ | 0.9809 | None |
| M527T548_1 | 527.1581 | 9.13 | C18H32O16 | Raffinose | [M+Na]+ | 0.9977 | None |
| M539T319 | 539.1734 | 5.32 | C25H30O13 | Picroside III | [M+H]+ | 0.9938 | None |
| M539T840 | 539.3191 | 14 | C27H48O9 | 3-(.beta.-D-Galactopyranosyloxy)-2-hydroxypropyl (9Z,12Z)-octadeca-9,12-dienoate | [M+Na]+ | 0.993 | None |
| M539T592 | 539.4093 | 9.86 | C28H58O9 | Octapropylene glycol monobutyl ether | [M+H]+ | 0.7403 | None |
| M542T790 | 542.3218 | 13.17 | C26H50NO7P | .beta.-Linoleoyl-.alpha.-glycerophosphorylcholine | [M+Na]+ | 0.9598 | None |
| M543T66 | 543.132 | 1.1 | C18H32O16 | Melezitose | [M+K]+ | 0.9991 | None |
| M545T671 | 545.3835 | 11.19 | C17H24N2O | N-Cyclohexyl-N'-(1,2,3,4-tetrahydro-1-naphthalenyl)urea | [2M+H]+ | 0.8383 | None |
| M546T886 | 546.353 | 14.76 | C26H54NO7P | 18:0 Lyso PC | [M+Na]+ | 0.9702 | None |
| M561T922 | 561.355 | 15.36 | C26H55N2O7P | Methylcarbamyl PAF C-16 | [M+Na]+ | 0.9964 | None |
| M563T314 | 563.2098 | 5.24 | C30H36O9 | Nimbin | [M+Na]+ | 0.9964 | None |
| M569T577 | 569.4198 | 9.61 | C35H62O3 | Tinogard TS | [M+K]+ | 0.735 | Into_Blood |
| M571T249 | 571.3564 | 4.15 | C31H46N4O6 | 6-Benzyl-3-butan-2-yl-9-(7-hydroxy-6-oxooctyl)-1,4,7,10-tetrazabicyclo[10.4.0]hexadecane-2,5,8,11-tetrone | [M+H]+ | 0.835 | None |
| M576T451 | 576.3895 | 7.52 | C30H49N5O6 | C-6 NBD ceramide | [M+H]+ | 0.9742 | None |
| M578T463 | 578.405 | 7.72 | C30H51N5O6 | C-6 NBD-dihydroceramide | [M+H]+ | 0.9684 | None |
| M579T247 | 579.1497 | 4.12 | C30H26O12 | Procyanidin B2 | [M+H]+ | 0.9958 | None |
| M581T343 | 581.15 | 5.72 | C26H28O15 | Leucoside | [M+H]+ | 0.9966 | None |
| M591T339 | 591.2047 | 5.64 | C27H36O13 | Gallomyrtucommulone C | [M+Na]+ | 0.9838 | None |
| M591T478 | 591.4253 | 7.97 | C30H58N2O9 | Descladinoseazithromycin | [M+H]+ | 0.8051 | None |
| M593T342 | 593.2204 | 5.7 | C22H42N4O8S2 | Lipodel | [M+K]+ | 0.9595 | None |
| M597T316 | 597.1447 | 5.26 | C26H28O16 | Quercetin-3-O-.beta.-D-xylopyranosyl (1->6)-.beta.-D-glucopyranoside | [M+H]+ | 0.9948 | None |
| M601T371 | 601.1528 | 6.19 | C27H30O14 | 1,5-Anhydro-1-(7-((6-deoxyhexopyranosyl)oxy)-5-hydroxy-2-(4-hydroxyphenyl)-4-oxo-4H-chromen-8-yl)hexitol | [M+Na]+ | 0.9711 | None |
| M601T321 | 601.2684 | 5.35 | C29H42N2O10 | 4,5-Dihydro-19-hydroxygeldanamycin | [M+Na]+ | 0.8965 | None |
| M601T475 | 601.4097 | 7.92 | C36H60O9 | Fasciculic acid B | [M+H-2H2O]+ | 0.7236 | None |
| M603T383 | 603.2046 | 6.39 | C28H36O13 | (-)-Syringaresinol-4-O-.beta.-D-glucopyranoside | [M+Na]+ | 0.9941 | None |
| M605T577 | 605.4406 | 9.61 | C36H60O7 | (2R,3S,4S,5R,6R)-2-(hydroxymethyl)-6-[[(8R,10R,12S,13R,14S,17S)-12-hydroxy-4,4,8,10,14-pentamethyl-17-[(2Z)-6-methylhepta-2,5-dien-2-yl]-2,3,5,6,7,9,11,12,13,15,16,17-dodecahydro-1H-cyclopenta[a]phenanthren-3-yl]oxy]oxane-3,4,5-triol | [M+H]+ | 0.7558 | Into_Blood |
| M605T650 | 605.4408 | 10.84 | C36H60O7 | Ginsenoside Rh3 | [M+H]+ | 0.9241 | None |
| M611T349_1 | 611.1606 | 5.81 | C27H30O16 | 2-(3,4-Dihydroxyphenyl)-5-hydroxy-7-methoxy-4-oxo-4H-chromen-3-yl 2-O-.beta.-D-xylopyranosyl-.beta.-D-glucopyranoside | [M+H]+ | 0.9871 | None |
| M611T320 | 611.1606 | 5.34 | C27H30O16 | Sophoraflavonoloside | [M+H]+ | 0.9868 | None |
| M619T316 | 619.1266 | 5.26 | C26H28O16 | Peltatoside | [M+Na]+ | 0.987 | None |
| M619T462 | 619.4202 | 7.69 | C48H78O18 | 1-O-(3-((6-O-(6-Deoxyhexopyranosyl)hexopyranosyl)oxy)-23-hydroxy-28-oxoolean-12-en-28-yl)hexopyranose | [M+H-C12H20O10]+ | 0.8447 | None |
| M621T332 | 621.2154 | 5.53 | C32H38O11 | MCULE-1175390561 | [M+Na]+ | 0.9698 | None |
| M637T419 | 637.4303 | 6.98 | C20H30O3 | Isosteviol | [2M+H]+ | 0.703 | None |
| M645T827 | 645.4335 | 13.78 | C36H62O8 | Ginsenoside C-K | [M+Na]+ | 0.9913 | None |
| M649T577_2 | 649.2181 | 9.61 | C24H42O21 | Maltotetraose | [M+H-H2O]+ | 0.8987 | Into_Blood |
| M685T775 | 685.4285 | 12.92 | C36H60O12 | 1-O-(1,3,23,24,25-Pentahydroxy-28-oxo-9,19-cyclolanostan-28-yl)hexopyranose | [M+H]+ | 0.9412 | None |
| M689T74 | 689.2111 | 1.23 | C24H42O21 | Glycan MC=h4 | [M+Na]+ | 0.9667 | Into_Blood |
| M699T750 | 699.3563 | 12.5 | C33H56O14 | Gingerglycolipid A | [M+Na]+ | 0.9831 | None |
| M708T482 | 708.3714 | 8.03 | C36H55N5O7 | MCULE-8260133435 | [M+K]+ | 0.8477 | None |
| M747T671 | 747.4287 | 11.19 | C37H64O16 | 1-O-(2,5,8,11,14-Penta(butan-2-yl)-4,7,10,13,16-pentaoxo-3,6,9,12,15-pentaoxaheptadecan-1-oyl)pentitol | [M+H-H2O]+ | 0.9267 | None |
| M786T576 | 785.5032 | 9.61 | C42H72O13 | (2S,3R,4S,5S,6R)-2-[(2R,3R,4S,5S,6R)-4,5-dihydroxy-2-[[(3S,5R,8R,10R,12R,13R,14R,17S)-12-hydroxy-17-(2-hydroxy-6-methylhept-5-en-2-yl)-4,4,8,10,14-pentamethyl-2,3,5,6,7,9,11,12,13,15,16,17-dodecahydro-1H-cyclopenta[a]phenanthren-3-yl]oxy]-6-(hydroxymethyl)oxan-3-yl]oxy-6-(hydroxymethyl)oxane-3,4,5-triol | [M+H]+ | 0.8017 | Into_Blood |
| M786T472 | 785.504 | 7.87 | C42H72O13 | NCGC00381107-01 | [M+H]+ | 0.8065 | None |
| M791T755 | 791.4915 | 12.58 | C40H75O10P | PG(16:0/18:2(9Z,12Z)) | [M+2Na-H]+ | 0.9826 | None |
| M805T597 | 805.4235 | 9.95 | C41H66O14 | 1-O-((2.beta.,3.beta.,5.Xi.,9.Xi.,18.Xi.)-2,23-Dihydroxy-28-oxo-3-(.beta.-D-xylopyranosyloxy)olean-12-en-28-yl)-.beta.-D-glucopyranose | [M+Na]+ | 0.7762 | None |
| M812T707 | 812.4785 | 11.79 | C42H66O14 | NCGC00385211-01 | [M+NH4]+ | 0.9157 | None |
| M817T708 | 817.4339 | 11.79 | C42H66O14 | 1-O-(3-((6-Deoxyhexopyranosyl)oxy)-27-hydroxy-27,28-dioxours-12-en-28-yl)hexopyranose | [M+Na]+ | 0.952 | None |
| M821T517 | 821.4654 | 8.62 | C42H70O14 | ACon1_001975 | [M+Na]+ | 0.9878 | None |
| M839T599 | 839.4078 | 9.98 | C42H62O17 | Licoricesaponin g2 | [M+H]+ | 0.7369 | None |
| M865T499 | 865.4914 | 8.31 | C42H66N8O10 | Cyclo(alanylisoleucylprolylleucylleucylserylphenylalanylthreonyl) | [M+Na]+ | 0.8961 | None |
| M909T455 | 909.4811 | 7.59 | C45H74O17 | Asparanin B | [M+Na]+ | 0.7731 | None |
| M922T765 | 921.5178 | 12.76 | C50H74O14 | Pesticide4_doramectin_c50h74o14_dectomax | [M+Na]+ | 0.93 | None |
| M945T624 | 944.5206 | 10.4 | C47H74O18 | Chikusetsusaponin IV | [M+NH4]+ | 0.9964 | None |
| M948T650 | 947.5564 | 10.84 | C48H82O18 | MEGxp0_001460 | [M+H]+ | 0.8658 | None |
| M956T414 | 955.5225 | 6.89 | C47H80O18 | MCULE-9853404806 | [M+Na]+ | 0.9075 | Into_Blood |
| M970T634 | 969.5384 | 10.57 | C48H82O18 | NCGC00385113-01 | [M+Na]+ | 0.9122 | Into_Blood |
| M975T601_1 | 974.5312 | 10.02 | C48H76O19 | 1-O-((3.beta.,5.Xi.,9.Xi.,18.Xi.)-3-((3-O-Hexopyranosylhexopyranuronosyl)oxy)-28-oxoolean-12-en-28-yl)hexopyranose | [M+NH4]+ | 0.8979 | None |
| M986T405 | 985.5335 | 6.76 | C48H82O19 | (2s,3r,4s,5s,6r)-2-[(2r,3r,4s,5s,6r)-2-[[(3s,6s,8r,10r,12r,13r,14r,17s)-3,12-dihydroxy-4,4,8,10,14-pentamethyl-17-[(2s)-6-methyl-2-[(2s,3r,4s,5s,6r)-3,4,5-trihydroxy-6-(hydroxymethyl)oxan-2-yl]oxyhept-5-en-2-yl]-2,3,5,6,7,9,11,12,13,15,16,17-dodecahydro-1h-cyclopenta[a]phenanthren-6-yl]oxy]-4,5-dihydroxy-6-(hydroxymethyl)oxan-3-yl]oxy-6-(hydroxymethyl)oxane-3,4,5-triol | [M+Na]+ | 0.8972 | None |
| M1092T577 | 1091.5991 | 9.62 | C54H92O23 | MCULE-6182244684 | [M-H2O+H]+ | 0.8517 | Into_Blood |
| M1102T592 | 1101.581 | 9.86 | C53H90O22 | MEGxp0_001459 | [M+Na]+ | 0.9029 | Into_Blood |

# Supplementary Table 4. Chemical components identified in YHP aqueous extract and YHP-medicated serum using UHPLC-HRMS in anion analysis.

| No. | m/z | RT min | Formula | compound name | adduct | score | Into Blood or Only In Blood or None |
| --- | --- | --- | --- | --- | --- | --- | --- |
| M355T282_2 | 355.0668 | 4.69 | C15H16O10 | Coumaroyl + c6h9o8 (isomer of 844, 845, 846) | [M-H]- | 0.7998 | Into_Blood |
| M577T310 | 577.1348 | 5.16 | C30H26O12 | Procyanidin B2 | [M-H]- | 0.9919 | None |
| M289T326_4 | 289.0715 | 5.43 | C15H14O6.H2O | (+)-catechin hydrate | [M-H]- | 0.9835 | None |
| M381T379_2 | 381.1763 | 6.31 | C16H30O10 | Foliachinenoside I | [M-H]- | 0.937 | None |
| M206T459_3 | 206.0815 | 7.66 | C11H13NO3 | N-Acetyl-D-phenylalanine | [M-H]- | 0.981 | Into_Blood |
| M477T485_2 | 477.14 | 8.09 | C23H26O11 | Calceolarioside A | [M-H]- | 0.9212 | None |
| M932T618 | 931.5264 | 10.3 | C47H80O18 | Sanchinoside R1 | [M-H]- | 0.7183 | None |
| M845T664 | 845.4896 | 11.07 | C42H72O14 | Ginsenoside Rg1 | [M+HCOO]- | 0.8131 | None |
| M845T727 | 845.4895 | 12.12 | C42H72O14 | Ginsenoside Rf | [M+HCOO]- | 0.8773 | None |
| M769T738_3 | 769.4733 | 12.3 | C41H70O13 | 20(r)-notoginsenoside R2 | [M-H]- | 0.9391 | None |
| M946T752 | 945.5322 | 12.54 | C48H82O18 | Ginsenoside Rd | [M-H]- | 0.999 | None |
| M765T823 | 765.4788 | 13.72 | C42H70O12 | Ginsenoside Rg6 | [M-H]- | 0.8625 | None |
| M269T876 | 269.0453 | 14.6 | C21H20O9 | Frangulin A | [M-H-C6H10O4]- | 0.999 | Into_Blood |
| M169T257 | 169.0131 | 4.28 | C22H18O11 | (-)-Gallocatechin 3-gallate | [M-H-C15H12O6]- | 0.9981 | None |
| M93T331 | 93.0334 | 5.52 | C6H6O | Phenol | [M-H]- | 0.999 | None |
| M101T670 | 101.0232 | 11.16 | C6H10O7 | Galacturonic acid | [M-H-C2H4O4]- | 0.955 | None |
| M102T58 | 102.0548 | 0.97 | C4H9NO2 | 3-Aminobutanoic acid | [M-H]- | 0.9856 | None |
| M104T56 | 104.0341 | 0.93 | C3H7NO3 | Serine | [M-H]- | 0.9113 | None |
| M107T698 | 107.049 | 11.63 | C8H8O3 | 3-Hydroxyphenylacetic acid | [M-H-CO2]- | 0.9951 | Into_Blood |
| M109T1183 | 109.0284 | 19.71 | C6H6O2 | Hydroquinone | [M-H]- | 0.9979 | None |
| M109T352 | 109.0282 | 5.86 | C6H6O2 | Pyrocatechol | [M-H]- | 0.9981 | None |
| M110T223 | 110.0236 | 3.71 | C15H17NO4 | Actiphenol | [M-H-C10H12O2]- | 0.9461 | None |
| M111T153_2 | 111.0076 | 2.55 | C6H6O6 | cis-Aconitic acid | [M-H-CH2O3]- | 0.9986 | None |
| M111T135 | 111.0076 | 2.25 | C6H8O7 | Isocitric acid | [M-H-CH4O4]- | 0.9989 | None |
| M113T1127 | 113.0232 | 18.78 | C14H17NO5 | Diethyl 2-[(2-hydroxyanilino)methylene]malonate | [M-H-C9H11NO2]- | 0.9988 | None |
| M114T58 | 114.0185 | 0.97 | C4H5NO3 | Maleamate | [M-H]- | 0.7431 | None |
| M114T240 | 114.0547 | 4 | C5H9NO2 | D-Proline | [M-H]- | 0.991 | None |
| M115T251 | 115.0024 | 4.18 | C4H4O4 | Maleic acid | [M-H]- | 0.9574 | None |
| M115T161 | 115.0025 | 2.68 | C4H4O4 | Fumaric acid | [M-H]- | 0.9943 | None |
| M115T178 | 115.0389 | 2.97 | C5H8O3 | Levulinic acid | [M-H]- | 0.7786 | None |
| M116T449 | 116.0494 | 7.49 | C8H7N | Indole | [M-H]- | 0.9676 | None |
| M119T314 | 119.049 | 5.23 | C8H8O | 4-Vinylphenol | [M-H]- | 0.9773 | None |
| M120T266_2 | 120.0442 | 4.43 | C8H9NO3S | 3-(Methylsulfonamido)benzaldehyde | [M-H-CH2SO2]- | 0.9791 | Into_Blood |
| M121T357 | 121.0283 | 5.96 | C7H6O5S | 1-Phenol-2-sulfonic acid, 4-formyl- | [M-H-SO3]- | 0.8429 | None |
| M123T1125_1 | 123.0076 | 18.75 | C19H22FNO3 | (-)-trans-4-[4-(4'-Fluorophenyl)-3-piperidinylmethoxy]-2-methoxyphenol | [M-H-C13H18FN]- | 0.8394 | None |
| M124T360 | 124.0061 | 6 | C2H7NO3S | Taurine | [M-H]- | 0.9959 | None |
| M125T57 | 124.9902 | 0.95 | C2H6O4S | Ethyl sulfate | [M-H]- | 0.8116 | None |
| M127T217 | 127.0025 | 3.62 | C6H6O7 | Epoxytricarballylic acid | [M-H-CH2O3]- | 0.8966 | Into_Blood |
| M128T194 | 128.0338 | 3.23 | C5H7NO3 | Pidolic acid | [M-H]- | 0.9974 | None |
| M130T708 | 130.0651 | 11.8 | C15H19N3O | 5-Methyl-2-[(4-methyl-1-piperazinyl)carbonyl]-1H-indole | [M-H-C6H10N2O]- | 0.984 | None |
| M130T642 | 130.0861 | 10.71 | C6H13NO2 | L-Norleucine | [M-H]- | 0.844 | None |
| M130T788 | 130.0863 | 13.13 | C6H13NO2 | L-Isoleucine | [M-H]- | 0.8321 | None |
| M131T235 | 131.0337 | 3.92 | C5H8O4 | Methylsuccinic acid | [M-H]- | 0.9959 | None |
| M131T794 | 131.0336 | 13.24 | C5H10O5 | .alpha.-D-Xylopyranose | [M-H-H2O]- | 0.8487 | None |
| M131T377 | 131.0702 | 6.28 | C6H12O3 | L-Leucic acid | [M-H]- | 0.9514 | None |
| M134T271 | 134.0462 | 4.51 | C8H9N5O2 | 3-Adenin-9-ylpropionic acid | [M-H-C3H4O2]- | 0.9947 | None |
| M135T618 | 135.0438 | 10.3 | C16H14O6 | 7-O-Methyleriodictyol | [M-H-C8H6O4]- | 0.9989 | None |
| M135T271 | 135.0444 | 4.52 | C9H8O4 | 5-Formyl-2-hydroxy-3-methylbenzoic acid | [M-H-CO2]- | 0.997 | Into_Blood |
| M136T432 | 136.0393 | 7.2 | C8H9NO4S | 2-[(Methylsulfonamido]benzoic acid | [M-H-CH2SO2]- | 0.8196 | None |
| M138T312 | 138.0184 | 5.21 | C6H5NO3 | 3-Hydroxypicolinic acid | [M-H]- | 0.9712 | None |
| M139T158 | 139.0137 | 2.64 | C5H4N2O3 | 5-Hydroxypyrazine-2-carboxylic acid | [M-H]- | 0.9683 | None |
| M139T112 | 139.0502 | 1.87 | C6H8N2O2 | 3,5-Dimethyl-1H-pyrazole-4-carboxylic acid | [M-H]- | 0.894 | None |
| M142T818 | 142.0496 | 13.64 | C10H19NO4 | 2-(tert-Butoxycarbonylamino)valeric acid | [M-H-C4H10O]- | 0.7419 | None |
| M144T570 | 144.0441 | 9.49 | C10H7NO3 | 4-(5-Oxazolyl)benzoic acid | [M-H-CO2]- | 0.9972 | None |
| M144T305 | 144.0442 | 5.08 | C11H11NO2 | N-Methylindoleacetic acid | [M-H-C2H4O]- | 0.9981 | None |
| M145T284 | 145.0496 | 4.73 | C6H10O4 | 2-Methylglutaric acid | [M-H]- | 0.9868 | None |
| M145T54 | 145.0966 | 0.9 | C6H14N2O2 | Lysine | [M-H]- | 0.9512 | None |
| M147T297 | 147.0286 | 4.96 | C5H8O5 | 3-Hydroxyglutaric acid | [M-H]- | 0.8225 | None |
| M147T325 | 147.0288 | 5.41 | C5H6O4 | trans-Glutaconic acid | [M+OH]- | 0.8217 | None |
| M147T117 | 147.0288 | 1.96 | C5H8O5 | L-2-Hydroxyglutaric acid | [M-H]- | 0.9768 | None |
| M147T502_2 | 147.044 | 8.36 | C9H8O2 | 4-Hydroxycinnamyl aldehyde | [M-H]- | 0.9814 | None |
| M147T525 | 147.0441 | 8.75 | C10H8O3 | Hymecromone | [M-H-CO]- | 0.9846 | None |
| M147T542 | 147.0441 | 9.03 | C9H10O3 | DL-3-Phenyllactic acid | [M-H-H2O]- | 0.9755 | None |
| M148T122_3 | 148.0427 | 2.03 | C9H7NO4 | 2,3-Dihydro-3-methyl-2-oxo-5-benzoxazolecarboxylic acid | [M-H-CO2]- | 0.9811 | None |
| M149T64 | 149.0082 | 1.07 | C4H6O6 | (S,S)-Tartaric acid | [M-H]- | 0.9406 | None |
| M151T619 | 151.0388 | 10.32 | C8H8O3 | 3-Hydroxy-4-methoxybenzaldehyde | [M-H]- | 0.9073 | None |
| M151T745 | 151.039 | 12.42 | C8H8O3 | 2,4-Cresotic acid | [M-H]- | 0.9735 | None |
| M151T698 | 151.039 | 11.63 | C8H8O3 | 2-Hydroxyphenylacetic acid | [M-H]- | 0.9982 | Into_Blood |
| M151T61 | 151.0601 | 1.01 | C5H12O5 | L-Lyxitol | [M-H]- | 0.9821 | None |
| M152T340 | 152.0343 | 5.66 | C7H7NO3 | 3-Hydroxyanthranilic acid | [M-H]- | 0.9312 | None |
| M152T309 | 152.0342 | 5.15 | C7H7NO3 | 4-Aminosalicylic acid | [M-H]- | 0.9529 | None |
| M153T404 | 153.0183 | 6.74 | C7H6O4 | .beta.-Resorcylic acid | [M-H]- | 0.9729 | Into_Blood |
| M153T386 | 153.0177 | 6.43 | C20H22O11 | Odontoside | [M-H-C13H16O7]- | 0.9967 | None |
| M154T496 | 154.0135 | 8.27 | C6H5NO4 | 4-Nitrocatechol | [M-H]- | 0.9966 | None |
| M154T287 | 154.0135 | 4.79 | C6H5NO4 | Pyrrole-2,5-dicarboxylic acid | [M-H]- | 0.9926 | Into_Blood |
| M155T211 | 154.9975 | 3.51 | C6H4O5 | 2,5-Furandicarboxylic acid | [M-H]- | 0.9937 | None |
| M157T389 | 157.0496 | 6.48 | C7H12O5 | 3-Isopropylmalic acid | [M-H-H2O]- | 0.9961 | None |
| M157T645 | 157.0858 | 10.75 | C8H14O3 | 2-((2R)-2-Hydroxycyclohexyl)acetic acid | [M-H]- | 0.784 | None |
| M158T522 | 158.0596 | 8.71 | C10H9NO | Indole-3-acetaldehyde | [M-H]- | 0.8708 | None |
| M158T336 | 158.0812 | 5.59 | C7H13NO3 | N-Isovaleroylglycine | [M-H]- | 0.9878 | None |
| M158T288 | 158.0814 | 4.79 | C7H13NO3 | N-Acetylvaline | [M-H]- | 0.9983 | None |
| M159T425 | 159.0653 | 7.09 | C7H12O4 | 3,3-Dimethylglutaric acid | [M-H]- | 0.9374 | None |
| M159T470 | 159.1017 | 7.83 | C8H16O3 | 5-Hydroxyvalproic acid | [M-H]- | 0.9475 | None |
| M161T178_1 | 161.0446 | 2.97 | C6H10O5 | 3-Hydroxy-3-methylglutaric acid | [M-H]- | 0.9838 | None |
| M161T1121 | 161.0446 | 18.68 | C12H22O11 | Glycan MC=h2 | [M-2H-H2O]2- | 0.8902 | None |
| M161T65 | 161.0447 | 1.08 | C12H22O11 | Lactulose | [M-H-C6H12O6]- | 0.9207 | None |
| M161T523 | 161.0447 | 8.72 | C6H12O6 | myo-Inositol | [M-H-H2O]- | 0.8704 | None |
| M161T541_2 | 161.0598 | 9.02 | C23H28O11 | Methyl (3R,4R,5R)-3,4-dihydroxy-5-((6-O-((2E)-3-phenylprop-2-enoyl)-.beta.-D-glucopyranosyl)oxy)cyclohex-1-ene-1-carboxylate | [M-H-C13H18O9]- | 0.9833 | None |
| M162T200 | 161.9856 | 3.34 | C4H5NO4S | 6-Methyl-1,2,3-oxathiazin-4(3H)-one 2,2-dioxide | [M-H]- | 0.9249 | None |
| M163T327 | 163.0391 | 5.46 | C24H30O12 | 8-O-4-Hydroxycinnamoylharpagide | [M-H-C15H22O9]- | 0.9983 | None |
| M164T317 | 164.0345 | 5.28 | C8H7NO3 | 2-Formamidobenzoic acid | [M-H]- | 0.9552 | None |
| M165T59 | 165.0395 | 0.98 | C5H10O6 | D-Arabinonic acid | [M-H]- | 0.9827 | None |
| M165T755 | 165.0547 | 12.58 | C9H10O3 | m-Anisylacetic acid | [M-H]- | 0.9469 | None |
| M166T217_1 | 166.0136 | 3.62 | C7H5NO4 | Dipicolinic acid | [M-H]- | 0.9298 | None |
| M167T181 | 167.0201 | 3.02 | C5H4N4O3 | Uric acid | [M-H]- | 0.9979 | None |
| M167T236 | 167.0334 | 3.94 | C8H8O4 | Vanillic acid | [M-H]- | 0.9829 | None |
| M168T703 | 168.0293 | 11.71 | C7H7NO4 | 3-Methylpyrrole-2,4-dicarboxylic Acid | [M-H]- | 0.9834 | None |
| M169T425 | 169.0499 | 7.09 | C8H10O4 | Penicillic acid | [M-H]- | 0.823 | None |
| M171T754 | 171.1017 | 12.57 | C9H16O3 | (2E)-4-Hydroxynon-2-enoic acid | [M-H]- | 0.939 | None |
| M171T881 | 171.1016 | 14.68 | C9H16O3 | 8-Oxononanoic acid | [M-H]- | 0.9806 | None |
| M172T393 | 172.097 | 6.54 | C8H15NO3 | N-Acetyl-D-norleucine | [M-H]- | 0.9966 | None |
| M173T176 | 173.0082 | 2.94 | C6H8O7 | Citric acid | [M-H-H2O]- | 0.9646 | Into_Blood |
| M173T388 | 173.0446 | 6.46 | C16H18O9 | (1r,3R,4s,5S)-4-(((2E)-3-(3,4-Dihydroxyphenyl)prop-2-enoyl)oxy)-1,3,5-trihydroxycyclohexanecarboxylic acid | [M-H-C9H8O4]- | 0.9854 | None |
| M173T550 | 173.081 | 9.16 | C8H14O4 | 2-Propylglutaric acid | [M-H]- | 0.9142 | None |
| M174T59 | 174.0874 | 0.99 | C6H13N3O3 | L-Citrulline | [M-H]- | 0.9994 | None |
| M175T448 | 175.0239 | 7.46 | C6H8O6 | D-Glucurono-3,6-lactone | [M-H]- | 0.7497 | Into_Blood |
| M175T258_1 | 175.0241 | 4.29 | C6H8O6 | Araboascorbic acid | [M-H]- | 0.9347 | None |
| M177T434 | 177.0186 | 7.23 | C15H12FNO4 | 3-[2-(4-Fluoroanilino)-2-oxoethoxy]benzoic acid | [M-H-C6H6FN]- | 0.9876 | None |
| M178T340_2 | 178.0864 | 5.67 | C10H13NO2 | N-Acetyltyramine | [M-H]- | 0.9388 | None |
| M179T82 | 179.055 | 1.36 | C6H12O6 | D-Allose | [M-H]- | 0.8581 | None |
| M179T218_2 | 179.055 | 3.63 | C6H12O6 | Levulose | [M-H]- | 0.9478 | None |
| M179T470 | 179.055 | 7.84 | C6H12O6 | D-(-)-Tagatose | [M-H]- | 0.9476 | None |
| M179T733 | 179.0704 | 12.22 | C10H12O3 | 2-Methoxydihydrocinnamic acid | [M-H]- | 0.7754 | None |
| M180T265 | 180.0657 | 4.41 | C9H11NO3 | DL-o-Tyrosine | [M-H]- | 0.8183 | None |
| M181T311 | 181.0134 | 5.18 | C8H6O5 | 4-Hydroxyisophthalic acid | [M-H]- | 0.9821 | None |
| M181T59 | 181.0709 | 0.99 | C6H14O6 | Galactitol | [M-H]- | 0.9858 | None |
| M183T337 | 183.029 | 5.61 | C8H8O5 | Methyl gallate | [M-H]- | 0.9883 | None |
| M183T286 | 183.029 | 4.76 | C8H8O5 | 4-O-Methylgallic acid | [M-H]- | 0.9959 | None |
| M183T443 | 183.0654 | 7.39 | C9H12O4 | Spiro[3.3]heptane-2,2-dicarboxylic acid | [M-H]- | 0.9279 | None |
| M185T717 | 185.081 | 11.94 | C9H14O4 | Gabapentin related compound E | [M-H]- | 0.976 | None |
| M185T844 | 185.1172 | 14.06 | C18H30O3 | 9-Oxo-10(E),12(E)-octadecadienoic acid | [M-H-C8H12]- | 0.7686 | None |
| M187T830 | 187.0392 | 13.83 | C19H20O7 | 2,3a,7b,12-Tetrahydroxy-5-methyl-3a,4,5,6,7a,7b-hexahydro-2H-tetrapheno[6a,7-b]oxirene-7,8(3H,12bH)-dione | [M-H-C8H12O4]- | 0.9958 | Into_Blood |
| M187T638 | 187.0967 | 10.64 | C12H22O6 | 9-Glyceryloxy-9-ketopelargonic acid | [M-H-C3H6O2]- | 0.9137 | None |
| M187T547 | 187.0967 | 9.11 | C9H16O4 | Azelaic acid | [M-H]- | 0.9965 | None |
| M187T242 | 187.108 | 4.03 | C8H16N2O3 | Glycyl-L-norleucine | [M-H]- | 0.9792 | None |
| M187T227 | 187.108 | 3.78 | C8H16N2O3 | Glycyl-Isoleucine | [M-H]- | 0.9704 | None |
| M187T839 | 187.1331 | 13.98 | C10H20O3 | 10-Hydroxydecanoic acid | [M-H]- | 0.9897 | None |
| M189T69_1 | 189.0033 | 1.15 | C6H6O7 | Garcinia lactone | [M-H]- | 0.9364 | None |
| M189T298 | 189.0549 | 4.97 | C11H10O3 | 7-Hydroxy-4,8-dimethylcoumarin | [M-H]- | 0.9977 | Into_Blood |
| M189T587 | 189.0546 | 9.78 | C11H10O3 | 6-Ethyl-4-hydroxycoumarin | [M-H]- | 0.9959 | None |
| M190T422 | 190.0501 | 7.03 | C10H9NO3 | 2-Oxoindole-3-acetate | [M-H]- | 0.9907 | None |
| M191T321_2 | 191.0552 | 5.35 | C16H18O9 | 4-O-Caffeoylquinic acid | [M-H-C9H6O3]- | 0.9966 | None |
| M191T66_3 | 191.0553 | 1.09 | C7H12O6 | Quinate | [M-H]- | 0.9994 | Into_Blood |
| M191T82 | 191.0553 | 1.36 | C16H18O9 | Neochlorogenic acid | [M-H-C9H6O3]- | 0.8153 | None |
| M191T790_2 | 191.0705 | 13.16 | C11H12O3 | Ethyl p-coumarate | [M-H]- | 0.8265 | None |
| M192T415_2 | 192.0654 | 6.92 | C10H11NO3 | Phenaceturic acid | [M-H]- | 0.9998 | Into_Blood |
| M193T60 | 193.0346 | 1 | C6H10O7 | 2-Keto-D-gluconic acid | [M-H]- | 0.9934 | None |
| M193T730 | 193.0497 | 12.16 | C10H10O4 | Ferulic acid | [M-H]- | 0.9403 | None |
| M195T80 | 195.0502 | 1.33 | C6H12O7 | Dextronic acid | [M-H]- | 0.9818 | None |
| M195T552 | 195.0656 | 9.2 | C24H32O7 | 5-Hydroxy-2,2,6,6-tetramethyl-4-[2-methyl-1-(2,4,6-trihydroxy-3-isobutyrylphenyl)propyl]cyclohex-4-ene-1,3-quinone | [M-H-C14H20O3]- | 0.7478 | None |
| M195T759 | 195.1018 | 12.66 | C12H20O4 | 9-Carbomethoxydec-9-enoic acid | [M-H-CH4O]- | 0.7403 | None |
| M195T542 | 195.1019 | 9.03 | C12H16O5 | Propylurofuranic acid | [M-H-CO2]- | 0.8411 | None |
| M197T462 | 197.0448 | 7.7 | C9H10O5 | Ethyl gallate | [M-H]- | 0.9802 | None |
| M197T603 | 197.0812 | 10.05 | C10H14O4 | (+)-Camphanic acid | [M-H]- | 0.7036 | None |
| M197T873 | 197.1175 | 14.55 | C11H20O4 | 2-Hexylglutaric acid | [M-H-H2O]- | 0.9613 | None |
| M199T830 | 199.0389 | 13.83 | C13H8O4 | Purrenone | [M-H-CO]- | 0.9804 | None |
| M199T853 | 199.1331 | 14.21 | C11H20O3 | (2E)-5-Hydroxyundec-2-enoic acid | [M-H]- | 0.7831 | None |
| M201T537 | 201.1123 | 8.95 | C18H32O5 | 12,15-Octadecadienoic acid, 7,9,10-trihydroxy- | [M-H-C8H14O]- | 0.997 | None |
| M201T703 | 201.1124 | 11.71 | C10H18O4 | Sebacic acid | [M-H]- | 0.9857 | None |
| M201T221 | 201.1237 | 3.68 | C9H18N2O3 | Ala-Ile | [M-H]- | 0.9913 | None |
| M201T240 | 201.1237 | 4 | C9H18N2O3 | Alanyl-Leucine | [M-H]- | 0.9917 | None |
| M202T281 | 202.0825 | 4.69 | C12H13NO2 | Indolebutyric acid | [M-H]- | 0.7002 | Into_Blood |
| M203T330 | 203.0818 | 5.49 | C11H12N2O2 | Tryptophan | [M-H]- | 0.8423 | None |
| M203T506 | 203.0818 | 8.43 | C11H12N2O2 | L-Tryptophan | [M-H]- | 0.9951 | None |
| M203T685 | 203.1281 | 11.41 | C10H20O4 | 3,5-Dihydroxycapric acid | [M-H]- | 0.8941 | None |
| M205T70 | 205.0346 | 1.17 | C7H10O7 | (R)-2-Hydroxybutane-1,2,4-tricarboxylate | [M-H]- | 0.7674 | None |
| M205T371 | 205.0499 | 6.18 | C17H20O9 | Cnidioside A | [M-H-C6H10O5]- | 0.7041 | None |
| M207T868 | 207.1383 | 14.46 | C13H20O2 | 4,4,7a-Trimethyl-3a,5,6,7-tetrahydro-3H-indene-1-carboxylic acid | [M-H]- | 0.9273 | None |
| M208T432 | 208.0244 | 7.2 | C9H7NO5 | 2-Nitrophenylpyruvic acid | [M-H]- | 0.7707 | None |
| M208T204 | 208.0608 | 3.4 | C10H11NO4 | 4-(1-Amino-2-carboxyethyl)benzoic acid | [M-H]- | 0.7816 | None |
| M209T59 | 209.0298 | 0.98 | C6H10O8 | D-Galactarate | [M-H]- | 0.9784 | None |
| M209T817 | 209.1177 | 13.62 | C12H18O3 | Jasmonic acid | [M-H]- | 0.9814 | None |
| M210T422 | 210.0764 | 7.04 | C10H13NO4 | 3-Methoxytyrosine | [M-H]- | 0.9416 | None |
| M211T830 | 211.0394 | 13.83 | C15H12O6 | Violaceic acid | [M-H-C2H4O3]- | 0.9876 | None |
| M211T193_2 | 211.0717 | 3.22 | C9H14N2O5 | Pro-Asp | [M-H-H2O]- | 0.9582 | None |
| M211T770 | 211.1332 | 12.83 | C18H32O5 | (10E,15Z)-9,12,13-Trihydroxyoctadeca-10,15-dienoic acid | [M-H-C6H12O2]- | 0.9952 | None |
| M214T55 | 214.0484 | 0.92 | C5H14NO6P | sn-Glycero-3-phosphoethanolamine | [M-H]- | 0.9237 | None |
| M215T111 | 215.1142 | 1.85 | C8H16N4O3 | L-Arginine, N2-acetyl- | [M-H]- | 0.9178 | None |
| M217T456 | 217.1075 | 7.6 | C10H18O5 | 3-Hydroxysebacic acid | [M-H]- | 0.9131 | None |
| M221T408 | 221.0449 | 6.81 | C11H10O5 | 5-Carboxymellein | [M-H]- | 0.913 | Into_Blood |
| M221T65 | 221.0659 | 1.09 | C12H22O11 | Isomaltose | [M-H-C4H8O4]- | 0.9707 | None |
| M223T322_2 | 223.0606 | 5.36 | C11H12O5 | Sinapic acid | [M-H]- | 0.9905 | Into_Blood |
| M224T385 | 224.0195 | 6.41 | C9H7NO6 | 2-Monomethyl 3-nitrophthalate | [M-H]- | 0.7484 | None |
| M224T245 | 224.1035 | 4.08 | C16H15NO3 | 2-(4'-Dimethylaminobenzoyl)benzoic acid | [M-H-CO2]- | 0.7081 | Into_Blood |
| M227T328 | 227.0555 | 5.46 | C11H8N4O2 | 10-Methylisoalloxazine | [M-H]- | 0.9754 | None |
| M227T770 | 227.1283 | 12.83 | C12H20O4 | trans-Traumatic acid | [M-H]- | 0.9755 | None |
| M227T265 | 227.1396 | 4.41 | C11H20N2O3 | Pro-Leu | [M-H]- | 0.9224 | None |
| M229T361 | 229.0977 | 6.01 | C13H14N2O2 | Tetrahydroharman-3-carboxylic acid | [M-H]- | 0.9803 | None |
| M229T661 | 229.1076 | 11.01 | C11H18O5 | 2-(6-Hydroxyhexyl)-3-methylenesuccinic acid | [M-H]- | 0.9929 | None |
| M229T292 | 229.1547 | 4.87 | C11H22N2O3 | Val-Leu | [M-H]- | 0.9963 | None |
| M229T269 | 229.1552 | 4.48 | C11H22N2O3 | DL-Leu-DL-Val | [M-H]- | 0.9939 | None |
| M231T612 | 231.0768 | 10.2 | C12H12N2O3 | 1-Formyl-L-tryptophan | [M-H]- | 0.9215 | Into_Blood |
| M231T241 | 231.1345 | 4.01 | C10H20N2O4 | Thr-Leu | [M-H]- | 0.9697 | None |
| M233T764 | 233.0813 | 12.73 | C13H14O4 | 7-Acetyl-3,6-dihydroxy-8-methyltetralin-1-one | [M-H]- | 0.9621 | None |
| M233T291_2 | 233.0814 | 4.85 | C13H14O4 | 7-Hydroxy-3-(2-hydroxypropyl)-5-methylisocoumarin | [M-H]- | 0.9617 | None |
| M233T310 | 233.0814 | 5.16 | C8H14N2O6 | Ser-Glu | [M-H]- | 0.8869 | None |
| M236T256 | 236.0923 | 4.27 | C12H15NO4 | 2-((3aR,4S,7R,7aS)-1,3-Dioxohexahydro-1H-4,7-methanoisoindol-2(3H)-yl)propanoic acid | [M-H]- | 0.9281 | None |
| M239T830_2 | 239.0344 | 13.84 | C14H8O4 | Danthron | [M-H]- | 0.921 | Into_Blood |
| M243T619 | 243.1233 | 10.32 | C12H20O5 | 4-Oxododecanedioic acid | [M-H]- | 0.9388 | None |
| M243T350 | 243.171 | 5.83 | C12H24N2O3 | Leu-Leu | [M-H]- | 0.9899 | None |
| M243T319 | 243.171 | 5.32 | C12H24N2O3 | Ile-Leu | [M-H]- | 0.9916 | None |
| M245T776 | 245.045 | 12.93 | C13H12O6 | 3,7,8-Trihydroxy-3-methyl-1,4-dihydropyrano[4,3-b]chromen-10-one | [M-H-H2O]- | 0.9801 | Into_Blood |
| M245T506_2 | 245.0922 | 8.43 | C13H14N2O3 | N-Acetyl-D-tryptophan | [M-H]- | 0.9548 | None |
| M249T775 | 249.1492 | 12.92 | C15H22O3 | 7-Hydroxycostic acid | [M-H]- | 0.9892 | None |
| M249T974 | 249.1492 | 16.23 | C15H22O3 | 2-((1S,2S,4aR,8aS)-1-hydroxy-4a-methyl-8-methylenedecahydronaphthalen-2-yl)acrylic acid | [M-H]- | 0.9883 | None |
| M253T736 | 253.0501 | 12.27 | C14H10N2OS | 3H-spiro[1,3-benzothiazole-2,3'-indol]-2'(1'H)-one | [M-H]- | 0.9974 | None |
| M253T530 | 253.0505 | 8.84 | C15H10O4 | Chrysophanol | [M-H]- | 0.9983 | None |
| M253T836 | 253.0516 | 13.93 | C15H10O4 | Chrysin | [M-H]- | 0.9974 | None |
| M253T186 | 253.0926 | 3.1 | C16H14O3 | Ketoprofen | [M-H]- | 0.8511 | Into_Blood |
| M253T429 | 253.1078 | 7.14 | C12H18N2O2S | N-(2,5-Dimethoxyphenyl)-N'-propylthiourea | [M-H]- | 0.8364 | None |
| M253T794_4 | 253.1442 | 13.23 | C14H22O4 | trans-C 75 | [M-H]- | 0.994 | Into_Blood |
| M255T858 | 255.0296 | 14.29 | C14H8O5 | Purpurin | [M-H]- | 0.9283 | None |
| M258T223 | 258.0616 | 3.72 | C14H13NO2S | ML-099 | [M-H]- | 0.951 | None |
| M259T249 | 259.1297 | 4.16 | C11H20N2O5 | Glu-Leu | [M-H]- | 0.9832 | None |
| M262T470 | 262.1194 | 7.83 | C14H15F2N3 | 4-[5-(3,4-Difluorophenyl)-1H-imidazol-2-yl]piperidine | [M-H]- | 0.977 | Into_Blood |
| M263T529_2 | 263.019 | 8.82 | C12H8O7 | PURPUROGALLIN-4-CARBOXYLIC ACID | [M-H]- | 0.8916 | Into_Blood |
| M263T308 | 263.1398 | 5.14 | C14H20N2O3 | Phe-Val | [M-H]- | 0.9758 | None |
| M263T339_2 | 263.1399 | 5.66 | C14H20N2O3 | Valylphenylalanine | [M-H]- | 0.9964 | None |
| M265T281 | 265.1191 | 4.68 | C13H18N2O4 | Thr-Phe | [M-H]- | 0.9151 | None |
| M267T487 | 267.0296 | 8.12 | C15H10O6 | Datiscetin | [M-H-H2O]- | 0.7619 | None |
| M267T75 | 267.0355 | 1.25 | C4H6O5 | DL-Malic acid | [2M-H]- | 0.9799 | None |
| M267T97 | 267.0719 | 1.61 | C9H16O9 | 3-Deoxy-D-glycero-D-galacto-2-nonulosonic acid | [M-H]- | 0.7945 | None |
| M271T565 | 271.0595 | 9.42 | C15H12O5 | Naringenin | [M-H]- | 0.9936 | None |
| M271T66 | 271.1045 | 1.1 | C10H18N4O6 | Argininosuccinic acid | [M-H-H2O]- | 0.8467 | None |
| M272T459 | 272.0538 | 7.66 | C13H11N3O2S | 1H-Pyrazolo[3,4-b]pyridine-4-carboxylic acid, 1-ethyl-6-(2-thienyl)- | [M-H]- | 0.7004 | None |
| M273T525_1 | 273.0766 | 8.76 | C15H14O5 | 2,2'-Dihydroxy-4,4'-dimethoxybenzophenone | [M-H]- | 0.8231 | None |
| M277T428 | 277.0714 | 7.14 | C14H14O6 | 2,3,7,9-Tetrahydroxy-4a-methyl-3,4-dihydro-2H-benzo[c]chromen-6-one | [M-H]- | 0.8784 | None |
| M277T237 | 277.1195 | 3.95 | C14H18N2O4 | Tyr-Pro | [M-H]- | 0.9822 | None |
| M277T388 | 277.1555 | 6.47 | C15H22N2O3 | Phe-Leu | [M-H]- | 0.9921 | None |
| M277T409 | 277.1555 | 6.82 | C15H22N2O3 | Leu-Phe | [M-H]- | 0.9549 | None |
| M279T289 | 279.0985 | 4.81 | C13H16N2O5 | Aspartylphenylalanine | [M-H]- | 0.924 | None |
| M279T236 | 279.1348 | 3.94 | C14H20N2O4 | Tyr-Val | [M-H]- | 0.9537 | None |
| M281T403 | 281.0663 | 6.72 | C13H14O7 | Feruloyl Lactate | [M-H]- | 0.9621 | None |
| M281T394 | 281.1392 | 6.57 | C15H22O5 | Dihydrophaseic acid | [M-H]- | 0.9698 | None |
| M283T586 | 283.0247 | 9.76 | C15H8O6 | Rhein | [M-H]- | 0.8371 | Into_Blood |
| M283T923 | 283.0609 | 15.39 | C16H12O5 | 6a,12a-Dihydro-6H-[1,3]dioxolo[5,6][1]benzofuro[3,2-c]chromen-3-ol | [M-H]- | 0.9901 | None |
| M283T768 | 283.0609 | 12.81 | C16H12O5 | Parietin | [M-H]- | 0.9706 | None |
| M283T935 | 283.1701 | 15.59 | C14H24N2O4 | Oseltamivir acid | [M-H]- | 0.9495 | None |
| M284T588 | 284.0324 | 9.8 | C16H12O6 | Isokaempferide | [M-H-CH3]- | 0.9889 | Into_Blood |
| M285T814 | 285.0402 | 13.57 | C16H10O8 | 3,3',4,4'-Biphenyltetracarboxylic acid | [M-H-CO2]- | 0.9955 | Into_Blood |
| M285T757 | 285.0402 | 12.61 | C15H10O6 | Citreorosein | [M-H]- | 0.9978 | Into_Blood |
| M285T845 | 285.0401 | 14.09 | C21H19ClN2O8 | Oxazepam glucuronide | [M-H-C6H8O6]- | 0.9758 | None |
| M287T692_3 | 287.0558 | 11.53 | C15H12O6 | Eriodictyol | [M-H]- | 0.9961 | None |
| M288T139 | 288.0835 | 2.32 | C9H13N3O5 | Cytarabine | [M+HCOO]- | 0.991 | None |
| M288T160 | 288.1199 | 2.67 | C11H19N3O6 | Ophthalmate | [M-H]- | 0.9707 | None |
| M289T347_1 | 289.0717 | 5.78 | C15H14O6 | Epicatechin | [M-H]- | 0.965 | None |
| M290T271 | 290.088 | 4.51 | C17H13N3O2 | N-(4-Phenoxyphenyl)-2-pyrazinecarboxamide | [M-H]- | 0.8294 | None |
| M291T290_2 | 291.0985 | 4.83 | C19H16O3 | Coumatetralyl | [M-H]- | 0.7299 | None |
| M291T900 | 291.1963 | 15 | C18H30O4 | 9-HpOTrE | [M-H-H2O]- | 0.7997 | None |
| M293T224 | 293.1142 | 3.74 | C14H18N2O5 | Phe-Glu | [M-H]- | 0.9587 | None |
| M291T858_5 | 291.1963 | 14.3 | C18H28O3 | 9-OxoOTrE | [M-H]- | 0.9158 | None |
| M293T275 | 293.1505 | 4.59 | C15H22N2O4 | Ile-Tyr | [M-H]- | 0.9869 | None |
| M293T253 | 293.124 | 4.22 | C14H18N2O5 | H-gamma-glu-phe-oh | [M-H]- | 0.8886 | Into_Blood |
| M295T857 | 295.0244 | 14.29 | C12H12N2O3S2 | [(6-Methyl-benzothiazol-2-ylcarbomyl)methylsulfanyl]acetic acid | [M-H]- | 0.7937 | None |
| M295T197 | 295.1031 | 3.28 | C24H36O15 | 4-(.beta.-D-Glucopyranosyloxy)benzyl 3-(.beta.-D-glucopyranosyloxy)-2-hydroxy-3-methylbutanoate | [M-H-C13H16O6]- | 0.9467 | None |
| M295T421 | 295.1184 | 7.02 | C15H20O6 | (2E,4E)-5-(3,8-Dihydroxy-1,5-dimethyl-7-oxo-6-oxabicyclo[3.2.1]octan-8-yl)-3-methylpenta-2,4-dienoic acid | [M-H]- | 0.9737 | None |
| M297T861 | 297.0402 | 14.34 | C16H11ClN2O2 | 7-Chloro-8-methyl-2-(4-pyridinyl)-4-quinolinecarboxylic acid | [M-H]- | 0.9594 | Into_Blood |
| M297T975 | 297.2432 | 16.25 | C18H34O3 | Lactarinic acid | [M-H]- | 0.9502 | Into_Blood |
| M299T207_1 | 299.077 | 3.44 | C13H16O8 | Salicylic acid beta-D-glucoside | [M-H]- | 0.8394 | None |
| M300T64 | 300.0392 | 1.07 | C8H15NO9S | N-Acetyl-D-galactosamine 4-sulfate | [M-H]- | 0.9746 | None |
| M300T177 | 300.1198 | 2.95 | C12H21N3O6S | Glutathione ethyl ester | [M-H-H2S]- | 0.763 | None |
| M300T395_2 | 300.135 | 6.58 | C16H19N3O3 | Trp-Pro | [M-H]- | 0.9518 | None |
| M300T425 | 300.1926 | 7.08 | C14H27N3O4 | Leu-Gly-Leu | [M-H]- | 0.8876 | None |
| M300T273_3 | 300.1926 | 4.55 | C14H27N3O4 | Ile-Gly-Ile | [M-H]- | 0.7523 | None |
| M301T482 | 300.9987 | 8.03 | C14H6O8 | Ellagic acid | [M-H]- | 0.998 | None |
| M301T701 | 301.035 | 11.69 | C15H10O7 | Quercetin | [M-H]- | 0.9925 | None |
| M301T586 | 301.0714 | 9.76 | C16H14O6 | Hesperetin | [M-H]- | 0.9574 | None |
| M302T255 | 302.1146 | 4.25 | C15H17N3O4 | Glutamine, N2-(indol-3-ylacetyl)- | [M-H]- | 0.969 | None |
| M303T517 | 303.0507 | 8.61 | C15H12O7 | Taxifolin | [M-H]- | 0.9916 | None |
| M305T246 | 305.0664 | 4.1 | C15H14O7 | (-)-Epigallocatechin | [M-H]- | 0.9967 | Into_Blood |
| M305T333 | 305.1141 | 5.55 | C15H18N2O5 | IDE1 | [M-H]- | 0.8906 | None |
| M307T817 | 307.1911 | 13.61 | C18H28O4 | 16-hydroxy-9-oxooctadeca-10,12,14-trienoic acid | [M-H]- | 0.8425 | None |
| M309T213 | 309.1189 | 3.54 | C18H18N2O3 | Orantinib | [M-H]- | 0.7421 | Into_Blood |
| M309T817 | 309.2067 | 13.61 | C18H30O4 | FA 18:3+2o | [M-H]- | 0.9377 | Into_Blood |
| M310T234 | 310.1153 | 3.9 | C12H17N5O5 | N,N-Dimethylguanosine | [M-H]- | 0.9597 | None |
| M311T57_2 | 311.1093 | 0.95 | C17H16N4 | 1,3-Bis(2-benzimidazolyl)propane | [M+Cl]- | 0.9102 | None |
| M311T900 | 311.2224 | 15.01 | C18H32O4 | 9(S)-HPODE | [M-H]- | 0.9325 | None |
| M311T871_6 | 311.2225 | 14.51 | C18H34O5 | (Z)-6,9,10-Trihydroxyoctadec-7-enoic acid | [M-H-H2O]- | 0.9651 | None |
| M312T674_3 | 312.1238 | 11.24 | C18H19NO4 | Moupinamide | [M-H]- | 0.942 | None |
| M313T745 | 313.035 | 12.42 | C16H10O7 | Endocrocin | [M-H]- | 0.9888 | None |
| M313T898 | 313.2382 | 14.96 | C18H34O4 | 9,10-DiHOME | [M-H]- | 0.9747 | None |
| M313T966 | 313.2382 | 16.1 | C18H34O4 | Octadecanedioic acid | [M-H]- | 0.993 | None |
| M315T200 | 315.0721 | 3.34 | C13H16O9 | Gentisic acid 5-O-glucoside | [M-H]- | 0.7951 | Into_Blood |
| M317T604 | 317.0297 | 10.07 | C15H10O8 | Myricetin | [M-H]- | 0.9776 | None |
| M319T352 | 319.1396 | 5.87 | C14H24O8 | (5Z)-3-(.beta.-D-Glucopyranosyloxy)oct-5-enoic acid | [M-H]- | 0.8165 | None |
| M321T518 | 321.1552 | 8.63 | C23H42O11 | 3-Hydroxy-5-((3-hydroxy-6-methyl-5-(pentofuranosyloxy)octanoyl)oxy)-6-methyloctanoic acid | [M-H-C9H16O3]- | 0.8336 | None |
| M323T278_1 | 323.1344 | 4.64 | C20H20O4 | Corylifolinin | [M-H]- | 0.8176 | Into_Blood |
| M325T349_2 | 325.0927 | 5.82 | C21H28O13 | 2-O-Hexopyranosyl-6-O-((2E)-3-(4-hydroxyphenyl)prop-2-enoyl)hexopyranose | [M-H-C6H10O5]- | 0.9025 | Into_Blood |
| M325T781_1 | 325.1841 | 13.02 | C18H30O3S | 4-Laurylbesylic acid | [M-H]- | 0.9962 | None |
| M325T980_2 | 325.2171 | 16.33 | C22H32O3 | (.+/-.)20-HDoHE | [M-H-H2O]- | 0.89 | None |
| M327T67 | 327.093 | 1.12 | C17H16N2O5 | CAY10397 | [M-H]- | 0.8091 | None |
| M327T853 | 327.2175 | 14.21 | C18H32O5 | FA 18:2+3o | [M-H]- | 0.9582 | Into_Blood |
| M328T520 | 328.1189 | 8.67 | C18H19NO5 | N-trans-Feruloyloctopamine | [M-H]- | 0.9318 | None |
| M329T236 | 329.0876 | 3.94 | C14H18O9 | 4-(Hexopyranosyloxy)-3-methoxybenzoic acid | [M-H]- | 0.963 | Into_Blood |
| M329T862 | 329.2332 | 14.36 | C18H34O5 | FA 18:1+3o | [M-H]- | 0.9853 | None |
| M329T809 | 329.2332 | 13.49 | C18H34O5 | (Z)-9,10,11-Trihydroxy-12-octadecenoic acid | [M-H]- | 0.9936 | Into_Blood |
| M330T398 | 330.2033 | 6.64 | C15H29N3O5 | Thr-Val-Leu | [M-H]- | 0.8079 | None |
| M331T367_2 | 331.0822 | 6.11 | C17H16O7 | Evernic acid | [M-H]- | 0.9043 | None |
| M335T326 | 335.0771 | 5.43 | C15H14O6 | Catechin | [M+HCOO]- | 0.946 | None |
| M337T386_3 | 337.0927 | 6.43 | C16H18O8 | DS-017250 | [M-H]- | 0.9858 | None |
| M337T314_4 | 337.0927 | 5.24 | C16H18O8 | 3-p-coumaroylquinic acid | [M-H]- | 0.9938 | None |
| M339T1014 | 339.1998 | 16.91 | C22H28O3 | AKOS015960729 | [M-H]- | 0.9451 | None |
| M343T395 | 343.1397 | 6.58 | C16H26O9 | 2-(2-((Hexopyranosyloxy)methyl)-3-methylcyclopent-2-en-1-yl)-3-hydroxypropanoic acid | [M-H-H2O]- | 0.9245 | None |
| M344T170 | 344.0399 | 2.83 | C10H12N5O7P | Cyclic GMP | [M-H]- | 0.9661 | None |
| M345T530 | 345.0744 | 8.84 | C13H18N2O7S | Propylthiouracil glucuronide | [M-H]- | 0.838 | None |
| M345T682 | 345.228 | 11.37 | C18H32O5 | (11E,15Z)-9,10,13-Trihydroxy-11,15-octadecadienoic acid | [M+OH]- | 0.8957 | None |
| M346T143 | 346.0557 | 2.38 | C10H14N5O7P | 3'-AMP | [M-H]- | 0.9941 | None |
| M347T349 | 347.077 | 5.81 | C17H16O8 | Asterric acid | [M-H]- | 0.7989 | None |
| M347T58 | 347.2158 | 0.97 | C6H14N4O2 | Arginine | [2M-H]- | 0.9968 | None |
| M350T320 | 350.1721 | 5.33 | C22H25NO3 | AM 580 | [M-H]- | 0.8399 | Into_Blood |
| M351T51 | 350.9208 | 0.85 | CH6O6P2 | Medronic acid | [2M-H]- | 0.9742 | None |
| M353T271 | 353.0876 | 4.52 | C16H18O9 | Caffeoyl quinic acid | [M-H]- | 0.987 | None |
| M355T692 | 355.0434 | 11.53 | C22H16O12 | Fumarprotocetraric acid | [M-H-C4H4O4]- | 0.939 | None |
| M355T501_2 | 355.1032 | 8.35 | C15H18O7 | 1-O-Cinnamoylglucose | [M+HCOO]- | 0.7109 | None |
| M355T353 | 355.1033 | 5.89 | C16H20O9 | (Z)-2-.beta.-D-Glucopyranosyloxy-4-methoxycinnamic acid | [M-H]- | 0.8209 | Into_Blood |
| M357T461 | 357.0614 | 7.68 | C18H14O8 | Parellic acid | [M-H]- | 0.8488 | None |
| M357T664 | 357.0979 | 11.07 | C19H18O7 | 3-Hydroxy-7,8,2',3'-tetramethoxyflavone | [M-H]- | 0.7683 | None |
| M357T620 | 357.1005 | 10.33 | C19H18O7 | Corymbosin | [M-H]- | 0.8167 | None |
| M357T64 | 357.1036 | 1.07 | C12H22O12 | Lactobionic acid | [M-H]- | 0.9835 | None |
| M357T535 | 357.1341 | 8.92 | C20H22O6 | Pinoresinol | [M-H]- | 0.9719 | None |
| M359T386 | 359.0749 | 6.43 | C18H16O8 | Rosmarinic acid | [M-H]- | 0.8272 | None |
| M359T225 | 359.1347 | 3.75 | C16H24O9 | Junipediol A 8-glucoside | [M-H]- | 0.9666 | Into_Blood |
| M361T443_2 | 361.1655 | 7.39 | C20H26O6 | Secoisolariciresinol | [M-H]- | 0.9766 | None |
| M365T515 | 365.0875 | 8.59 | C17H18O9 | Rubinaphthin A | [M-H]- | 0.9827 | None |
| M366T441 | 366.149 | 7.35 | C20H21N3O4 | Trp-Tyr | [M-H]- | 0.8458 | None |
| M367T401 | 367.103 | 6.68 | C17H20O9 | 1,3,5-Trihydroxy-4-[(E)-3-(3-hydroxy-4-methoxyphenyl)acryloyl]oxycyclohexanecarboxylic acid | [M-H]- | 0.9952 | None |
| M367T416_2 | 367.1031 | 6.93 | C17H20O9 | 3-O-Feruloylquinic acid | [M-H]- | 0.9829 | None |
| M367T340_2 | 367.1031 | 5.66 | C17H20O9 | 5-Feruloylquinic acid | [M-H]- | 0.9902 | None |
| M367T457 | 367.1031 | 7.61 | C17H20O9 | 4-O-Feruloylquinic acid | [M-H]- | 0.9887 | None |
| M371T423 | 371.0981 | 7.06 | C16H20O10 | 3-(Benzoyloxy)-2-hydroxypropyl .beta.-D-glucopyranosiduronic acid | [M-H]- | 0.8852 | None |
| M371T502 | 371.1709 | 8.37 | C16H28N4O4S | Biocytin | [M-H]- | 0.953 | None |
| M373T840 | 373.0925 | 14 | C19H18O8 | Neobaicalein | [M-H]- | 0.9029 | None |
| M374T199 | 374.1567 | 3.32 | C24H22FNO2 | AM2201 2-hydroxyindole metabolite | [M-H]- | 0.7624 | None |
| M378T281 | 378.1668 | 4.68 | C23H25NO4 | Atalaphylline | [M-H]- | 0.745 | None |
| M380T238 | 380.156 | 3.96 | C22H21NO4 | CAY10581 | [M+OH]- | 0.8212 | None |
| M385T779 | 385.1865 | 12.98 | C19H30O8 | (3E)-4-((1S)-1-Hydroxy-2,6,6-trimethyl-4-oxocyclohex-2-en-1-yl)but-3-en-2-yl .beta.-D-glucopyranoside | [M-H]- | 0.9284 | None |
| M387T343_1 | 387.1295 | 5.71 | C17H24O10 | Geniposide | [M-H]- | 0.8438 | None |
| M393T595_2 | 393.1188 | 9.92 | C19H22O9 | 7-Acetyl-3,8-dihydroxy-6-methylnaphthalen-1-yl .beta.-D-glucopyranoside | [M-H]- | 0.9968 | None |
| M395T568 | 395.08 | 9.46 | C30H22O10 | Rhusflavanone | [M-H-C9H6O2]- | 0.8204 | None |
| M397T277 | 397.1136 | 4.62 | C27H28O13 | 3-O-Caffeoyl-4-O-sinapoylquinic acid | [M-H-C9H6O3]- | 0.9711 | None |
| M400T379 | 400.1509 | 6.31 | C25H23NO4 | JWH 081 N-pentanoic acid metabolite | [M-H]- | 0.7395 | None |
| M401T300 | 401.1086 | 5 | C16H20O9 | 2-Glucosyloxy-4-methoxycinnamic acid | [M+FA-H]- | 0.8621 | None |
| M403T628 | 403.1031 | 10.47 | C20H20O9 | Resveratrol 4'-O-D-glucuronide | [M-H]- | 0.8324 | None |
| M403T441 | 403.1606 | 7.35 | C18H28O10 | 1-O-((2E,4E)-9-Carboxy-8-hydroxy-2,7-dimethylnona-2,4-dienoyl)-.beta.-D-glucopyranose | [M-H]- | 0.7126 | None |
| M407T697 | 407.1343 | 11.62 | C20H24O9 | Torachrysone 8-glucoside | [M-H]- | 0.9951 | None |
| M411T766 | 411.0715 | 12.76 | C27H28O14 | 8-C-(2-Rhamnosyl-6-deoxyhexopyranosulyl)luteolin | [M-H-C6H12O5]- | 0.874 | None |
| M415T698 | 415.1031 | 11.64 | C21H20O9 | Chrysophanol 8-O-beta-D-glucoside | [M-H]- | 0.998 | None |
| M415T432 | 415.1607 | 7.21 | C19H28O10 | 2-Phenylethyl 2-O-.beta.-D-xylopyranosyl-.beta.-D-glucopyranoside | [M-H]- | 0.9674 | None |
| M417T485_2 | 417.1187 | 8.08 | C21H22O9 | Neoliquiritin | [M-H]- | 0.9399 | None |
| M417T379 | 417.153 | 6.31 | C28H36O13 | (-)-Syringaresinol-4-O-.beta.-D-glucopyranoside | [M-H-C6H10O5]- | 0.7662 | None |
| M419T50_2 | 418.9549 | 0.83 | C6H15O15P3 | D-myo-Inositol-1,2,6-triphosphate | [M-H]- | 0.8989 | None |
| M421T62 | 421.075 | 1.03 | C12H23O14P | Trehalose, 6-(dihydrogen phosphate) | [M-H]- | 0.9429 | None |
| M425T334 | 425.1662 | 5.56 | C16H28O10 | Prenyl arabinosyl-(1-&gt | [M+HCOO]- | 0.7865 | None |
| M431T379_1 | 431.0978 | 6.32 | C21H20O10 | Sophoricoside | [M-H]- | 0.9955 | None |
| M431T611_3 | 431.0978 | 10.19 | C21H20O10 | Emodin 8-glucoside | [M-H]- | 0.9722 | None |
| M431T669 | 431.0979 | 11.15 | C21H20O10 | Vitexin | [M-H]- | 0.955 | None |
| M431T326 | 431.1554 | 5.44 | C19H28O11 | Osmanthuside H | [M-H]- | 0.7153 | None |
| M431T364 | 431.1918 | 6.07 | C19H30O8 | 3-Hydroxy-3,5,5-trimethyl-4-(3-oxobut-1-en-1-ylidene)cyclohexyl .beta.-D-glucopyranoside | [M+HCOO]- | 0.8185 | None |
| M433T565_1 | 433.1135 | 9.42 | C21H22O10 | Naringenin 7-O-beta-D-glucoside | [M-H]- | 0.9952 | Into_Blood |
| M433T341 | 433.1137 | 5.68 | C21H22O10 | [(2r,3s,4s,5r,6s)-3,4,5-trihydroxy-6-(4-hydroxyphenoxy)oxan-2-yl]methyl (e)-3-(3,4-dihydroxyphenyl)prop-2-enoate | [M-H]- | 0.9349 | None |
| M435T603 | 435.1289 | 10.06 | C21H24O10 | Phlorizin | [M-H]- | 0.9964 | None |
| M435T413 | 435.1292 | 6.88 | C20H22O8 | Resveratroloside | [M+HCOO]- | 0.9956 | None |
| M435T247 | 435.1505 | 4.11 | C17H26O10 | Loganin | [M+HCOO]- | 0.7019 | None |
| M439T532 | 439.0667 | 8.86 | C19H12F4N4O2 | VX-702 | [M+Cl]- | 0.7207 | None |
| M441T526 | 441.1974 | 8.76 | C17H32O10 | Hexyl 6-O-pentopyranosylhexopyranoside | [M+HCOO]- | 0.9656 | None |
| M443T298 | 443.192 | 4.97 | C21H32O10 | 1'R,3'S,5'R,8'S,2E,4E)-Dihydrophaseic acid 3'-O-.beta.-D-glucopyranoside | [M-H]- | 0.9752 | None |
| M445T787 | 445.2077 | 13.12 | C22H30N4O6 | CAY10603 | [M-H]- | 0.7651 | None |
| M445T258 | 445.2302 | 4.29 | C25H34O7 | [7'-Formyl-3,4'-dihydroxy-6'-(hydroxymethyl)-4,4,7,8a-tetramethylspiro[2,3,4a,5,6,7-hexahydro-1H-naphthalene-8,2'-3H-1-benzofuran]-2-yl] acetate | [M-H]- | 0.7589 | None |
| M447T655 | 447.093 | 10.92 | C21H21O11 | Cyanidin-3-o-glucoside | [M-2H]- | 0.9434 | None |
| M447T463 | 447.093 | 7.72 | C21H20O11 | Luteolin 4'-glucoside | [M-H]- | 0.9882 | None |
| M447T545 | 447.093 | 9.08 | C21H20O11 | Kaempferol-3-o-glucoside | [M-H]- | 0.966 | None |
| M447T362_2 | 447.1505 | 6.03 | C18H26O10 | Benzyl alcohol + hex-pen | [M+HCOO]- | 0.7803 | None |
| M449T583 | 449.1087 | 9.71 | C21H22O11 | Eriodictyol-7-O-glucoside | [M-H]- | 0.9759 | Into_Blood |
| M451T232_2 | 451.1244 | 3.86 | C21H24O11 | Coatline B | [M-H]- | 0.9491 | None |
| M457T305_1 | 457.1714 | 5.09 | C21H26N6O4S | Mmv595321 | [M-H]- | 0.8948 | None |
| M457T570 | 457.1715 | 9.5 | C21H30O11 | 4-(Prop-2-en-1-yl)phenyl 6-O-hexopyranosylhexopyranoside | [M-H]- | 0.7134 | None |
| M461T674_2 | 461.1087 | 11.23 | C22H22O11 | .beta.-D-Glucopyranose, 6-[(2E)-3-phenyl-2-propenoate] 1-(3,4,5-trihydroxybenzoate) | [M-H]- | 0.9711 | None |
| M461T278 | 461.13 | 4.63 | C19H26O13 | Sibiricose A3 | [M-H]- | 0.9194 | None |
| M461T432 | 461.1663 | 7.21 | C19H28O10 | Icariside D1 | [M+HCOO]- | 0.9467 | Into_Blood |
| M463T484 | 463.0881 | 8.07 | C21H20O12 | Quercetin-3-o-glucoside | [M-H]- | 0.97 | Into_Blood |
| M467T745_1 | 467.0593 | 12.41 | C23H16O11 | Cromolyn | [M-H]- | 0.7314 | Only_Into_Blood |
| M469T600_2 | 469.2286 | 10.01 | C19H36O10 | Rhodiooctanoside | [M+HCOO]- | 0.9051 | None |
| M471T507 | 471.1504 | 8.45 | C21H28O12 | 4-O-((2E)-3-Phenylprop-2-enoyl)-.beta.-D-fructofuranosyl .alpha.-D-glucopyranoside | [M-H]- | 0.9638 | None |
| M473T555 | 473.1662 | 9.24 | C20H28O10 | Rosarin | [M+HCOO]- | 0.7545 | None |
| M473T420 | 473.1662 | 7 | C21H30O12 | 2-Hydroxy-4-(prop-2-en-1-yl)phenyl 6-O-.beta.-D-glucopyranosyl-.beta.-D-glucopyranoside | [M-H]- | 0.7899 | None |
| M475T100 | 475.13 | 1.67 | C21H28O10 | Grandidentoside | [M+Cl]- | 0.8455 | None |
| M475T450 | 475.182 | 7.49 | C20H30O10 | Phenethyl rutinoside | [M+HCOO]- | 0.9317 | None |
| M477T580 | 477.0992 | 9.66 | C22H22O12 | Nepetin 7-glucoside | [M-H]- | 0.9088 | None |
| M477T549 | 477.1036 | 9.15 | C22H23O12 | Petunidin 3-galactoside | [M-2H]- | 0.9788 | None |
| M477T559_4 | 477.1036 | 9.32 | C22H22O12 | Isorhamnetin-3-o-glucoside | [M-H]- | 0.9861 | None |
| M477T415 | 477.1036 | 6.92 | C22H22O12 | 6-O-((2E)-3-(4-Hydroxyphenyl)prop-2-enoyl)-1-O-(3,4,5-trihydroxybenzoyl)hexopyranose | [M-H]- | 0.7622 | None |
| M477T469 | 477.1036 | 7.82 | C22H22O12 | 2-(3,4-Dihydroxyphenyl)-5-hydroxy-7-methoxy-4-oxo-4H-chromen-3-yl hexopyranoside | [M-H]- | 0.7124 | None |
| M477T537 | 477.1765 | 8.95 | C24H30O10 | 4-(7-Hydroxy-3-(hydroxymethyl)-5-(3-hydroxypropyl)-2,3-dihydro-1-benzofuran-2-yl)-2-methoxyphenyl .beta.-D-xylopyranoside | [M-H]- | 0.7892 | None |
| M483T313 | 483.0778 | 5.21 | C27H24O18 | 1,3,6-Trigalloylglucose | [M-H-C7H4O4]- | 0.846 | None |
| M487T349 | 487.1455 | 5.82 | C21H28O13 | MCULE-9096711794 | [M-H]- | 0.9196 | None |
| M487T397 | 487.1819 | 6.62 | C21H30O10 | Lusitanicoside | [M+HCOO]- | 0.7478 | None |
| M489T322 | 489.1612 | 5.37 | C21H30O13 | 6'-O--D-Apiofuranosylsweroside | [M-H]- | 0.8315 | None |
| M491T745_1 | 491.1192 | 12.42 | C22H22O10 | Sissotrin | [M+HCOO]- | 0.9133 | None |
| M493T570 | 493.1482 | 9.5 | C19H28O12 | 3,4-Dimethoxyphenyl 6-O-pentopyranosylhexopyranoside | [M+HCOO]- | 0.7775 | None |
| M493T492 | 493.2289 | 8.2 | C21H36O10 | (3R)-3,7-Dimethylocta-1,6-dien-3-yl 6-O-.alpha.-L-arabinopyranosyl-.beta.-D-glucopyranoside | [M+HCOO]- | 0.8208 | None |
| M493T665 | 493.229 | 11.08 | C21H36O10 | (6,6-Dimethylbicyclo[3.1.1]heptan-2-yl)methyl 6-O-((2R,3R,4R)-3,4-dihydroxy-4-(hydroxymethyl)tetrahydrofuran-2-yl)-.beta.-D-glucopyranoside | [M+FA-H]- | 0.9628 | None |
| M493T694 | 493.229 | 11.57 | C21H36O10 | Atractyloside A | [M+HCO2]- | 0.9036 | None |
| M499T47_2 | 498.9213 | 0.79 | C6H16O18P4 | D-myo-Inositol-1,3,4,6-tetraphosphate | [M-H]- | 0.8351 | None |
| M499T472 | 499.0857 | 7.87 | C24H20O12 | 6,7'-Dihydroxy-2,2'-dioxo-2H,2'H-[8,8'-bichromen]-7-yl hexopyranoside | [M-H]- | 0.7493 | None |
| M503T72 | 503.1615 | 1.2 | C18H32O16 | 1-Kestose | [M-H]- | 0.8756 | None |
| M507T537 | 507.1128 | 8.95 | C23H24O13 | Syringetin-3-o-glucoside | [M-H]- | 0.8173 | None |
| M507T492 | 507.1501 | 8.2 | C24H28O12 | Specioside | [M-H]- | 0.7897 | None |
| M507T581 | 507.1637 | 9.68 | C22H32O11 | 4-Hydroxy-3-(3-methylbut-2-en-1-yl)phenyl 6-O-((2R,3R,4R)-3,4-dihydroxy-4-(hydroxymethyl)tetrahydrofuran-2-yl)-.beta.-D-glucopyranoside | [M+Cl]- | 0.7574 | None |
| M507T401 | 507.1866 | 6.68 | C25H32O11 | Ncgc00385759-01!(5s)-1,7-bis(3,4-dihydroxyphenyl)-5-[(2r,3r,4s,5s,6r)-3,4,5-trihydroxy-6-(hydroxymethyl)oxan-2-yl]oxyheptan-3-one | [M-H]- | 0.7923 | None |
| M509T385 | 509.2236 | 6.42 | C21H36O11 | 5-Hydroxy-1,7,7-trimethylbicyclo[2.2.1]heptan-2-yl 6-O-(3,4-dihydroxy-4-(hydroxymethyl)tetrahydrofuran-2-yl)hexopyranoside | [M+HCOO]- | 0.9366 | None |
| M509T468 | 509.2237 | 7.79 | C21H36O11 | (2E)-6-Hydroxy-2,6-dimethylocta-2,7-dien-1-yl 6-O-.alpha.-L-arabinopyranosyl-.beta.-D-glucopyranoside | [M+HCOO]- | 0.7642 | None |
| M517T723 | 517.0982 | 12.04 | C24H22O13 | Malonylgenistin | [M-H]- | 0.8234 | None |
| M517T380 | 517.1559 | 6.34 | C22H30O14 | 6-O-((2E)-3-(4-Hydroxy-3-methoxyphenyl)prop-2-enoyl)hex-2-ulofuranosyl hexopyranoside | [M-H]- | 0.8433 | None |
| M521T538 | 521.2027 | 8.97 | C26H34O11 | .beta.-D-Glucopyranoside, 3-[2,3-dihydro-2-(4-hydroxy-3-methoxyphenyl)-3-(hydroxymethyl)-7-methoxy-5-benzofuranyl]propyl | [M-H]- | 0.9376 | None |
| M523T520 | 523.2183 | 8.66 | C26H36O11 | 4-(2,6,6-Trimethyl-4-oxocyclohex-2-en-1-yl)butan-2-yl 6-O-(3,4,5-trihydroxybenzoyl)hexopyranoside | [M-H]- | 0.8057 | None |
| M533T381 | 533.1873 | 6.35 | C22H32O12 | Eugenol gentiobioside | [M+HCOO]- | 0.8075 | None |
| M539T892 | 539.0978 | 14.86 | C30H22O11 | (2S,3S)-8-[(2R,3S)-5,7-Dihydroxy-2-(4-hydroxyphenyl)-4-ketochroman-3-yl]-3,5,7-trihydroxy-2-(4-hydroxyphenyl)chroman-4-one | [M-H2O-H]- | 0.9928 | Into_Blood |
| M539T428 | 539.2131 | 7.14 | C26H36O12 | 3-(4-((1,3-Dihydroxy-1-(4-hydroxy-3-methoxyphenyl)propan-2-yl)oxy)-3-methoxyphenyl)propyl hexopyranoside | [M-H]- | 0.7826 | None |
| M539T415 | 539.2132 | 6.92 | C26H36O12 | Sonchifolignan A | [M-H]- | 0.8844 | None |
| M543T355 | 543.2291 | 5.92 | C33H33FO6 | Etalocib | [M-H]- | 0.7899 | None |
| M549T71 | 549.1668 | 1.19 | C18H32O16 | Raffinose | [M+HCO2]- | 0.9686 | None |
| M549T617 | 549.2914 | 10.29 | C26H46O12 | (2S,3R)-2,3,4-Trihydroxybutyl 2,6-di-O-acetyl-3-O-dodecanoyl-.beta.-D-mannopyranoside | [M-H]- | 0.8289 | None |
| M553T592 | 553.2937 | 9.87 | C32H44O9 | Ganoderic acid H | [M-H-H2O]- | 0.8638 | None |
| M559T494 | 559.1663 | 8.23 | C24H32O15 | 6-O-((2Z)-3-(4-Hydroxy-3-methoxyphenyl)prop-2-enoyl)-.beta.-D-fructofuranosyl 6-O-acetyl-.alpha.-D-glucopyranoside | [M-H]- | 0.9022 | Into_Blood |
| M561T944 | 561.3275 | 15.73 | C27H48O9 | MGMG 18:2 | [M+HCOO]- | 0.9156 | None |
| M565T478 | 565.1925 | 7.96 | C26H32O11 | 4-((2S,3S)-3-(Hydroxymethyl)-5-((1E)-3-hydroxyprop-1-en-1-yl)-7-methoxy-2,3-dihydro-1-benzofuran-2-yl)-2-methoxyphenyl hexopyranoside | [M+HCOO]- | 0.7389 | None |
| M567T481 | 567.208 | 8.02 | C26H34O11 | Indigoticoside A | [M+HCOO]- | 0.919 | None |
| M569T300 | 569.3414 | 5 | C34H50O7 | Carbenoxolone | [M-H]- | 0.9276 | None |
| M579T480_2 | 579.1352 | 8 | C26H28O15 | Kaempferol 3-O-vicianoside | [M-H]- | 0.9769 | Into_Blood |
| M579T326 | 579.1503 | 5.43 | C15H14O6 | (-)-Catechin | [2M-H]- | 0.9691 | None |
| M581T445 | 581.2236 | 7.41 | C28H38O13 | Lyoniresinol 9'-.beta.-D-glucopyranoside | [M-H]- | 0.9412 | None |
| M593T637 | 593.1497 | 10.61 | C27H30O15 | Oroxin b | [M-H]- | 0.8282 | None |
| M593T353 | 593.1509 | 5.88 | C27H30O15 | Safflor yellow A | [M-H]- | 0.7747 | None |
| M593T627_1 | 593.1509 | 10.46 | C27H30O15 | Apigenin 7-O-glucosylglucoside | [M-H]- | 0.9524 | None |
| M595T491 | 595.1666 | 8.19 | C27H32O15 | Cassiaside C | [M-H]- | 0.7177 | None |
| M607T810 | 607.1818 | 13.51 | C28H32O15 | Diosmetin-7-o-neohesperidoside | [M-H]- | 0.8924 | None |
| M609T492 | 609.1457 | 8.19 | C27H30O16 | 2-(3,4-Dihydroxyphenyl)-5-hydroxy-7-methoxy-4-oxo-4H-chromen-3-yl 2-O-.beta.-D-xylopyranosyl-.beta.-D-glucopyranoside | [M-H]- | 0.879 | None |
| M609T440 | 609.1459 | 7.34 | C27H30O16 | Kaempferol 3-o-sophoroside | [M-H]- | 0.9109 | None |
| M617T521 | 617.2658 | 8.69 | C31H40O10 | NCGC00381227-01 | [M+FA-H]- | 0.8404 | None |
| M621T386 | 621.1093 | 6.43 | C27H26O17 | Q63398889 | [M-H]- | 0.9703 | Only_Into_Blood |
| M623T530 | 623.1615 | 8.84 | C27H30O14 | Damnacanthol 3-O-.beta.-primeveroside | [M+HCOO]- | 0.8254 | None |
| M623T516 | 623.1615 | 8.6 | C28H32O16 | Isorhamnetin-3-o-rutinoside | [M-H]- | 0.8019 | None |
| M625T401 | 625.1407 | 6.68 | C27H30O17 | 2-(3,4-Dihydroxyphenyl)-5,7-dihydroxy-4-oxo-4H-chromen-3-yl 4-O-hexopyranosylhexopyranoside | [M-H]- | 0.9451 | None |
| M627T532 | 627.0988 | 8.86 | C16H11ClN2O3 | 5-[(7-Chloro-4-quinolinyl)amino]-2-hydroxybenzoic acid | [2M-H]- | 0.8135 | None |
| M627T436 | 627.2291 | 7.27 | C28H38O13 | (.+/-.)-Lyoniresinol 2a-O-.beta.-D-glucopyranoside | [M+FA-H]- | 0.9476 | None |
| M629T527 | 629.1145 | 8.78 | C29H26O16 | 6-O-p-Coumaroyl-1,2-digalloylglucose | [M-H]- | 0.8886 | None |
| M631T432 | 631.1068 | 7.2 | C16H12O7 | 3-Methylquercetin | [2M-H]- | 0.7286 | None |
| M631T881 | 631.3848 | 14.68 | C36H56O9 | (3.beta.,5.Xi.,9.Xi.,18.Xi.)-28-Hydroxy-28-oxoolean-12-en-3-yl .beta.-D-glucopyranosiduronic acid | [M-H]- | 0.9704 | None |
| M639T452 | 639.1565 | 7.53 | C28H32O17 | 2-(3,4-Dihydroxyphenyl)-5-hydroxy-7-methoxy-4-oxo-4H-chromen-3-yl 2-O-.beta.-D-glucopyranosyl-.beta.-D-glucopyranoside | [M-H]- | 0.8524 | None |
| M639T413 | 639.1927 | 6.88 | C29H36O16 | Purpureaside A | [M-H]- | 0.7614 | None |
| M653T497 | 653.2448 | 8.28 | C31H42O15 | 6-(2-Hydroxy-4-methyl-6-((.beta.-D-xylopyranosyloxy)methyl)phenoxy)-2-methoxy-3-(3-methyl-1-(.beta.-D-xylopyranosyloxy)butyl)benzoic acid | [M-H]- | 0.7614 | None |
| M665T72 | 665.214 | 1.2 | C24H42O21 | Stachyose | [M-H]- | 0.8774 | None |
| M683T97 | 683.2247 | 1.61 | C12H22O11 | D-(+)-Trehalose | [2M-H]- | 0.9472 | None |
| M685T698 | 685.3803 | 11.64 | C36H58O10 | Arjunglucoside II | [M+Cl]- | 0.9073 | None |
| M707T321 | 707.183 | 5.36 | C16H18O9 | Heriguard | [2M-H]- | 0.996 | None |
| M735T692 | 735.2141 | 11.53 | C34H40O18 | Helonioside B | [M-H]- | 0.9766 | None |
| M739T626 | 739.3394 | 10.43 | C36H54O13 | Apobioside | [M+HCOO]- | 0.8786 | None |
| M762T835 | 762.4084 | 13.91 | C36H61NO16 | Fumonisin A1 | [M-H]- | 0.7897 | None |
| M763T861 | 763.4273 | 14.35 | C41H64O13 | Momordin Ic | [M-H]- | 0.8102 | None |
| M765T778 | 765.443 | 12.97 | C41H66O13 | Soyasaponin IV | [M-H]- | 0.7585 | None |
| M793T828 | 793.4374 | 13.81 | C42H66O14 | Spinasaponin A | [M-H]- | 0.9163 | None |
| M793T770 | 793.4375 | 12.84 | C42H66O14 | MEGxp0_000202 | [M-H]- | 0.9544 | None |
| M805T747 | 805.4506 | 12.46 | C41H70O13 | Notoginsenoside R2 | [M+Cl]- | 0.8587 | None |
| M811T911 | 811.4842 | 15.19 | C42H70O12 | Ginsenoside Rg5 | [M+FA-H]- | 0.8314 | None |
| M815T553_2 | 815.4794 | 9.21 | C41H70O13 | Ginsenoside F3 | [M+FA-H]- | 0.7956 | None |
| M829T749 | 829.4945 | 12.48 | C42H72O13 | Panaxoside Rg2 | [M+HCOO]- | 0.9952 | None |
| M829T695 | 829.4953 | 11.59 | C42H72O13 | Ginsenoside F2 | [M+HCO2]- | 0.9674 | None |
| M861T531 | 861.1879 | 8.85 | C42H38O20 | Sennoside B | [M-H]- | 0.9496 | None |
| M863T700 | 863.2032 | 11.66 | C21H20O10 | Apigenin-4'-glucoside | [2M-H]- | 0.9635 | None |
| M885T655_2 | 885.4844 | 10.92 | C45H74O17 | Asparanin B | [M-H]- | 0.8197 | None |
| M925T754 | 925.4785 | 12.57 | C47H74O18 | Chikusetsusaponin IV | [M-H]- | 0.7197 | None |
| M946T632 | 945.5413 | 10.53 | C48H82O18 | MEGxp0_001460 | [M-H]- | 0.823 | None |
| M955T742_2 | 955.4893 | 12.36 | C48H76O19 | Chikusetsusaponin V | [M-H]- | 0.9253 | None |
| M962T587_1 | 961.5363 | 9.78 | C48H82O19 | (2s,3r,4s,5s,6r)-2-[(2r,3r,4s,5s,6r)-2-[[(3s,6s,8r,10r,12r,13r,14r,17s)-3,12-dihydroxy-4,4,8,10,14-pentamethyl-17-[(2s)-6-methyl-2-[(2s,3r,4s,5s,6r)-3,4,5-trihydroxy-6-(hydroxymethyl)oxan-2-yl]oxyhept-5-en-2-yl]-2,3,5,6,7,9,11,12,13,15,16,17-dodecahydro-1h-cyclopenta[a]phenanthren-6-yl]oxy]-4,5-dihydroxy-6-(hydroxymethyl)oxan-3-yl]oxy-6-(hydroxymethyl)oxane-3,4,5-triol | [M-H]- | 0.9061 | None |
| M962T826 | 961.5366 | 13.77 | C48H82O19 | Gypenoside xlvi | [M-H]- | 0.765 | None |
| M992T654 | 991.5473 | 10.89 | C48H82O18 | Ginsenoside Re | [M+HCOO]- | 0.9767 | None |
| M1088T738 | 1087.5319 | 12.3 | C52H82O21 | 1-O-((10-((6-Deoxyhexopyranosyl-(1->4)-[pentopyranosyl-(1->3)]hexopyranosyl)oxy)-6a,6b,9,9,12a-pentamethyl-2-methylidene-1,3,4,5,6,6a,6b,7,8,8a,9,10,11,12,12a,12b,13,14b-octadecahydropicen-4a(2H)-yl)carbonyl)hexopyranose | [M+HCOO]- | 0.8713 | None |
| M1106T716_1 | 1105.5786 | 11.93 | C54H88O22 | (3.beta.,5.Xi.,9.Xi.)-23,28-Dihydroxyoleana-11,13(18)-dien-3-yl hexopyranosyl-(1->4)-6-deoxyhexopyranosyl-(1->4)hexopyranosyl-(1->3)hexopyranoside | [M+OH]- | 0.9049 | None |
| M1118T698 | 1117.5422 | 11.63 | C54H86O24 | 1-O-((3.beta.,5.Xi.,9.Xi.,18.Xi.)-3-((.beta.-D-Glucopyranosyl-(1->2)-[.beta.-D-glucopyranosyl-(1->4)]-.beta.-D-glucopyranuronosyl)oxy)-28-oxoolean-12-en-28-yl)-.beta.-D-glucopyranose | [M-H]- | 0.9711 | None |
| M1122T614_1 | 1121.5738 | 10.23 | C53H88O22 | (3.beta.,5.Xi.,9.Xi.,16.beta.)-28-(.beta.-D-Glucopyranosyloxy)-16-hydroxyolean-12-en-3-yl .beta.-D-xylopyranosyl-(1->6)-.beta.-D-glucopyranosyl-(1->6)-.beta.-D-glucopyranoside | [M+HCOO]- | 0.8526 | None |
| M1124T595 | 1123.5894 | 9.91 | C53H90O22 | MEGxp0_001459 | [M+FA-H]- | 0.9147 | None |
| M1124T649_1 | 1123.5896 | 10.81 | C54H92O24 | Siamenoside i | [M-H]- | 0.9851 | None |
| M1124T580 | 1123.5899 | 9.66 | C53H90O22 | Ginsenoside Rb3 | [M+FA-H]- | 0.8877 | None |
| M1154T553 | 1153.6008 | 9.22 | C54H92O23 | Ginsenoside Rb1 | [M+FA-H]- | 0.9095 | None |
